# Head and Neck Clinical Signs Associated With Diseases: A Scoping Review

**DOI:** 10.1111/scd.70185

**Published:** 2026-05-14

**Authors:** Helena Miguel Cotter, Mateus José Dutra, Gustavo Davi Rabelo, Ana Carolina Prado Ribeiro, Marcio Ajudarte Lopes, Márcio Diniz‐Freitas, Luiz Paulo Kowalski, Alan Roger Santos‐Silva, Caique Mariano Pedroso

**Affiliations:** ^1^ Department of Dentistry Federal University of Santa Catarina Florianópolis Santa Catarina Brazil; ^2^ Oral Diagnosis Department, Piracicaba Dental School University of Campinas Piracicaba São Paulo Brazil; ^3^ São Paulo State Cancer Institute São Paulo University Medical School São Paulo Brazil; ^4^ Medical‐Surgical Dentistry Research Group (OMEQUI), Health Research Institute of Santiago de Compostela (IDIS) University of Santiago de Compostela (USC) Santiago de Compostela Spain; ^5^ AC Camargo Cancer Center São Paulo São Paulo Brazil; ^6^ Department of Head and Neck Surgery, School of Medicine, Hospital Das Clínicas University of São Paulo (HC‐FMUSP) São Paulo São Paulo Brazil

**Keywords:** head and neck, physical examination, rapid diagnostic test, scoping review

## Abstract

**Objective:**

Clinical signs observed during head and neck examination offer important diagnostic clues. This scoping review aimed to identify key clinical signs in this region associated with diseases and syndromes and to map them according to their location.

**Methods:**

An electronic literature search was performed in five databases (Embase, LILACS, PubMed, Scopus, and Web of Science) and in grey literature (Google Scholar and ProQuest). Studies were excluded if they did not involve humans, addressed non‐specific or non–head and neck signs, used auxiliary equipment during examination, were conducted outside clinical settings, lacked original data, or had no accessible full text. Clinical signs were categorized as ocular, cutaneous, intraoral, auricular, or other.

**Results:**

Overall, 54 studies met the criteria, identifying 43 distinct clinical signs. Intraoral signs were the most frequent (*n* = 15), with “strawberry tongue” being the most common. Cutaneous signs (10) included café‐au‐lait lesions, facial trichilemmomas, and Pemberton's sign. Ocular manifestations appeared in nine cases, with Horner's syndrome being the most reported. Other signs accounted for six reports, and auricular signs (three) were the least frequent, with Frank's sign most cited.

**Conclusion:**

This review underscores the diagnostic relevance of head and neck clinical signs as indicators of systemic diseases, including intraoral ulcers, café‐au‐lait macules, Kayser‐Fleischer rings, Frank's sign, malar rash, and port‐wine stains.

## 1 Introduction

The physical examination of the head and neck region offers valuable insights into a wide range of medical diseases. Visual signs observed in this anatomical area, such as mucosal lesions, skin manifestations, bruising, twitching, abnormal movements, or facial asymmetries, can provide important diagnostic clues. It is therefore essential that healthcare professionals be trained to recognize and interpret these signs accurately and promptly to ensure early diagnosis and appropriate clinical management [1, 2, 3, 4, 5].

These findings can serve as critical indicators of underlying diseases, ranging from infectious diseases and metabolic disorders to malignancies and systemic diseases [1, 2, 3, 4, 5]. In many cases, signs in the head and neck region represent some of the earliest external manifestations of systemic diseases. Their presence not only assists in localizing disease processes but also contributes to assessing disease severity and progression [6, 7]. Despite their clinical importance, many of these clinical signs remain underrecognized in routine practice, particularly those that are subtle, uncommon, or that overlap with other conditions. A comprehensive understanding of these signs can enhance diagnostic accuracy, support clinical reasoning, and promote a more comprehensive, patient‐centered approach to care [6, 7].

Although the literature includes studies focusing on individual clinical signs, the broader diagnostic implications of head and neck findings have not been thoroughly synthesized. These signs often manifest as visible or palpable changes and may reflect either systemic or localized diseases [8, 9, 10]. Although individual clinical signs have been described in isolated reports and specialty‐specific literature, a synthesis mapping these signs according to anatomical location and systemic associations has not previously been performed. This review addresses that gap by providing an integrated framework to support clinical recognition. This scoping review aims to systematically identify and map the clinical signs observable in the head and neck region that are associated with diseases and syndromes, providing an overview of their diagnostic utility and relevance in both medical and dental practice.

To evaluate the existing evidence concerning clinical signs present in the head and neck region associated with systemic diseases and syndromes, the following review question was formulated: “What are the key clinical signs in the head and neck region observed during physical examination that are associated with diseases?”

## 2 Methods

This scoping review was conducted in accordance with the JBI methodology recommendations [11], and followed the reporting guidelines outlined in the Preferred Reporting Items for Systematic Reviews and Meta‐Analyses extension for Scoping Reviews (PRISMA‐ScR) [12, 13]. A research protocol was preregistered in the Open Science Framework (OSF) database (https://osf.io/5pwnx/).

### 2.1 Eligibility Criteria

The research question was formulated using the PCC framework (Population, Concept, and Context): (P): patients undergoing physical examination, including both adults and children in different healthcare settings; (C): clinical signs specific to the head and neck region observed during physical examination; and (C): clinical or healthcare settings (hospitals, clinics, or other practice environments).

This review included both primary and secondary research studies, such as case reports, observational studies, and reviews, provided they focused on clinical signs identified during physical examination. For this review, a clinical sign was defined as any observed indicator of disease detected solely through physical examination, without the aid of auxiliary diagnostic tools. Only signs directly attributable to the disease itself were included, whereas signs representing indirect consequences or complications of the disease were excluded, such as lymphadenopathies, facial asymmetries due to mass effect, or signs resulting from secondary infections or treatment‐related changes. No restriction on the language or publication date was applied.

The following exclusion criteria were applied: (1) Studies not involving human participants; (2) Clinical signs observed in areas other than the head and neck region or not specific to the disease; (3) Studies that did not involve physical examination or physical examination performed with auxiliary equipment; (4) Studies conducted outside clinical or medical examination settings, such as community health surveys; (5) Books, conference abstracts, opinion articles, technique articles, posters, guidelines, and reviews that did not specifically address clinical signs in the head and neck region as a primary focus; (6) Full‐text access unavailable, despite efforts to contact the corresponding authors.

### 2.2 Information Sources and Search Strategy

An electronic literature search strategy was performed on January sixth, 2025, using the following five databases: Embase, LILACS (in Spanish: Literatura Latinoamericana y del Caribe en Ciencias de la Salud), PubMed, Scopus, and Web of Science. In addition, gray literature was searched using Google Scholar and ProQuest Dissertations and Theses Global (ProQuest). The complete search strategies and terms used are detailed in Supporting Information 1.

### 2.3 Selection of Source of Evidence

All references were managed using EndNote X9 (Thomson Reuters, Philadelphia, PA, USA) to identify and remove duplicates. The selection process was then performed according to the eligibility criteria using online software (Rayyan, Qatar Computing Research Institute, Qatar) [14].

Two independent reviewers conducted a two‐phase selection process. In phase‐1, the two reviewers independently evaluated the titles and abstracts according to the eligibility criteria. In phase‐2, the same two reviewers applied the eligibility criteria after reading the remaining full‐text studies. Any discrepancies were resolved by a consensus discussion with a third reviewer before reaching a final decision in both phases.

### 2.4 Data Charting Process and Data Items

Two independent reviewers used a previously designed extraction form to collect data from the included studies and cross‐checked the data in a consensus discussion to ensure the integrity of the collected data. The following information was extracted from the included studies: authors, year of publication, country, study design (sample size/patients with sign), clinical sign, main characteristics, and associated disease (Supporting Information 2) and summarized in Tables 1, 2, 3, 4, 5.

**TABLE 1:** Synthesis of ocular clinical signs.

Clinical sign	Main characteristics	Associated diseases	Number of studies
**Blue sclera**	Sclera takes on a bluish color, due to a thinning of the sclera, allowing the color of the underlying tissue (choroid) to be visible.	Ehlers‐Danlos syndrome	2
**Coloboma**	Lack of tissue during fetal development results in visible defects in the eye structures, such as the iris, eyelids, or optic nerve.	Joubert syndrome and Treacher Collins syndrome	3
**One‐and‐a‐half syndrome**	Combination of symptoms: 1) conjugate horizontal gaze palsy (one‐and‐a‐half syndrome; inability to move both eyes horizontally in one direction); 2) ipsilateral internuclear ophthalmoplegia inability of the eye on the same side of the lesion to adduct move towards the nose), and 3) ipsilateral lower motor neuron‐like facial palsy (weakness of the facial muscles on the same side as the lesion).	Multiple sclerosis	1
**Heliotropic erythema / sign**	Purplish rash of the upper eyelids, with symmetrical distribution, and may be accompanied by edema.	Dermatomyosis	2
**Horner's syndrome**	Triad of miosis, partial ptosis and loss of hemifacial sweating.	Metastatic squamous cell carcinoma in the tonsil; neuroblastoma, ganglioneuroblastoma or esthesioneuroblastoma; internal carotid artery dissection; stenosis of the distal part of the right vertebral artery; metastatic breast cancer	5
**Kayser‐Fleischer ring**	Golden‐brown ring that appears around the edge of the cornea, that reflects copper deposition on the brain.	Wilson's disease	2
**Megalocornea**	Corneal diameter larger than normal (>13 mm)	Marfan syndrome	1
**Sclerocornea**	The cornea is opaque and resembles the sclera.	HCCS mutations with MLS syndrome	1
**Supranuclear gaze palsy**	Difficulty or inability to perform voluntary eye movements, while reflex eye movements remain normal.	Nieman‐Pic disease type C	1

**TABLE 2:** Synthesis of cutaneous clinical signs.

Clinical sign	Main characteristics	Associated diseases	Number of studies
**Acantose nigricans**	Skin condition characterized by dark, thick, velvety patches, usually in the folds of the body, such as the neck	Cushing's syndrome	1
**Café au lait lesions / macules**	Uniformly hyperpigmented macules (Neurofibromatosis‐1) and irregular bordes, also called “Coast of Maine” borders (McCune‐Albright syndrome).	Neurofibromatosis‐1 and McCune‐Albright syndrome	2
**Harlequin syndrome**	Asymmetric sweating and flushing on the neck and face.	Disruption of vasomotor and sudomotor sympathetic activity after thoracic epidurals	1
**Lupus pernio**	Chronic, reddish to violet, hardened plaques that can be found mainly on the nose, cheeks, and ears.	Sarcoidosis	1
**Malar rash**	Rash that appears on the face, covering the cheeks and the tip of the nose, and has a butterfly appearance due to its shape	Lupus erythematous	1
**Multiple facial trichilemmomas**	Flesh‐colored papules on the face.	Cowden syndrome	2
**Nasal tip necrosis**	Nasal tip necrosis	Hansen's disease (lepromatous leprosy)	1
**Pemberton's sign**	Facial erythema and edema when arms are elevated above the head.	Superior vena cava syndrome	2
**Port wine sign**	Well‐defined macular lesion initially pink, with a smooth surface that, unlike hemangiomas, partially blanches with pressure. The lesion develops proportionally with the child and usually gets darker in color. The skin over the port wine sign can present nodularity or hypertrophy.	Sturge–Weber Syndrome and Klippel–Trenaunay Syndrome	1
**Raspberry‐like papilomas**	Soft, pedunculated growths with numerous finger‐like projections, often resembling a raspberry, typically appear at junctions between skin and mucous membranes, such as the lips, perioral region, and around the eyes.	Goltz syndrome	1

**TABLE 3:** Synthesis of intraoral clinical signs.

Clinical sign	Main characteristics	Associated diseases	Number of studies
**Capillary malformation of the lower lip**	Erythematous‐violaceous macule of the lower Lip	CLAPO syndrome (Capillary vascular malformation of the lower lip, Lymphatic malformations of the head and neck, Asymmetry, and Partial or generalized Overgrowth)	1
**Cocaine‐induced midline destructive lesions**	Ranges from intranasal crusting, foul exudate, epistaxis, nasal scabs, saddle nose deformities, nasocutaneous fistulas, necrotizing ulcerative lesions, and septal perforation. In more severe cases, the destruction extends to the middle and superior turbinates, the lateral wall of the nose, and the hard palate.	Cocaine addiction	1
**False cheilitis**	Unilateral, slightly elevated pattern and present as a fissured papule of the commissure.	Secondary syphilis	1
**Multiple neurofibroma**	Benign tumors, which grow in peripheral nerves.	Neurofibromatois type 1 or Von Recklinghausen's neurofibromatosis	2
**Multiple osteomas**	Numerous benign bone tumors.	Gardner's syndrome	1
**Multiple papillomatous nodules / oral papillomas**	Wart‐like bumps on the tongue, gums, back of the throat, and tonsils.	Cowden syndrome	2
**Palatal defect**	Defect on hard and/or soft palate, causing buconasal fistula	Granulomatosis with Polyangiitis	1
**Perioral frecklin**	Brown to blue–gray macules primarily affect the vermilion zone, the labial and buccal mucosa, and the tongue.	Peutz–Jeghers syndrome	1
**Recurrent oral ulceration**	Multiple ulcers, of variable size, occur extensively on the buccal membrane, tongue, palate and in the oropharynx, classically painful, surrounded by erythema and the larger ones heal with scarring.	Behçet's disease	1
**Strawberry tongue**	Red, swollen tongue with prominent bumps	Kawasaki disease	4
**Tongue hamartoma, multiple frenula, cleft lip/palate, upper lip notch**	Tongue hamartoma (abnormal arrangement of normal tissues native to the tongue, sometimes forming a nodule or polyp); multiple frenula (variations or double frenulum); cleft lip/palate (defects where the tissues in the lip and/or palate don't fully fuse together during early pregnancy, resulting in an opening or gap); upper lip notch (minor or incomplete cleft lip).	Oral‐Facial‐Digital Syndrome Type VI	1
**Unilateral cyanosis of the tongue**	Discoloration of half of the tongue.	Vasculitis or occlusion of lingual artery	1

**TABLE 4:** Synthesis of auricular clinical signs.

Clinical sign	Main characteristics	Associated diseases	Number of studies
**Erythematous lesion of the retroauricular skin**	Protruding, mildly bulging and progressive erythematous lesion of the retroauricular skin and the auricula, painful when palpated.	Sweet's syndrome (acute febrile neutrophilic dermatosis)	1
**Frank sign**	Diagonal earlobe creases.	Atherosclerosis; cardiovascular disease; polycystic ovary syndrome	3
**Milian ear sign**	Well‐demarcated tender indurated, erythematous, and edematous swelling of both ears and face.	Erysipela	1

**TABLE 5:** Synthesis of other clinical signs.

Clinical sign	Main characteristics	Associated diseases	Number of studies
**Erythematous lesion of the skin**	Elevated, tender plaques and nodules in the head and neck area.	Sweet's syndrome (acute febrile neutrophilic dermatosis)	1
**Congenital infiltrating lipomatosis of the face**	Thickened buccal subcutaneous and palatal submucosa fat	Congenital infiltrating lipomatosis	1
**Moon face**	Swollen, round, or puffy face.	Cushing's syndrome	1
**Septal perforation**	Hole in the cartilage or bone that separates the two nostrils.	Granulomatosis with polyangiitis	1
**Tullio's phenomenon**	Sound‐induced dizziness, vertigo, or nystagmus (rapid eye movements).	Superior canal dehiscence syndrome	1
**Unilateral facial swelling and vesicles**	Facial swelling centered in the right submandibular area extending to the cheek and parotid regions with multiple erythematous, crusted vesicular eruptions, extending to the ipsilateral tragus, helix and pinna of the ear.	Ramsey Hunt syndrome	1

### 2.5 Synthesis of Results

A narrative synthesis was conducted. Clinical signs were categorized into five anatomical locations within the head and neck region: (1) ocular; (2) cutaneous; (3) intraoral; (4) auricular; and (5) other.

## 3 Results

### 3.1 Study Selection

**FIGURE 1:**
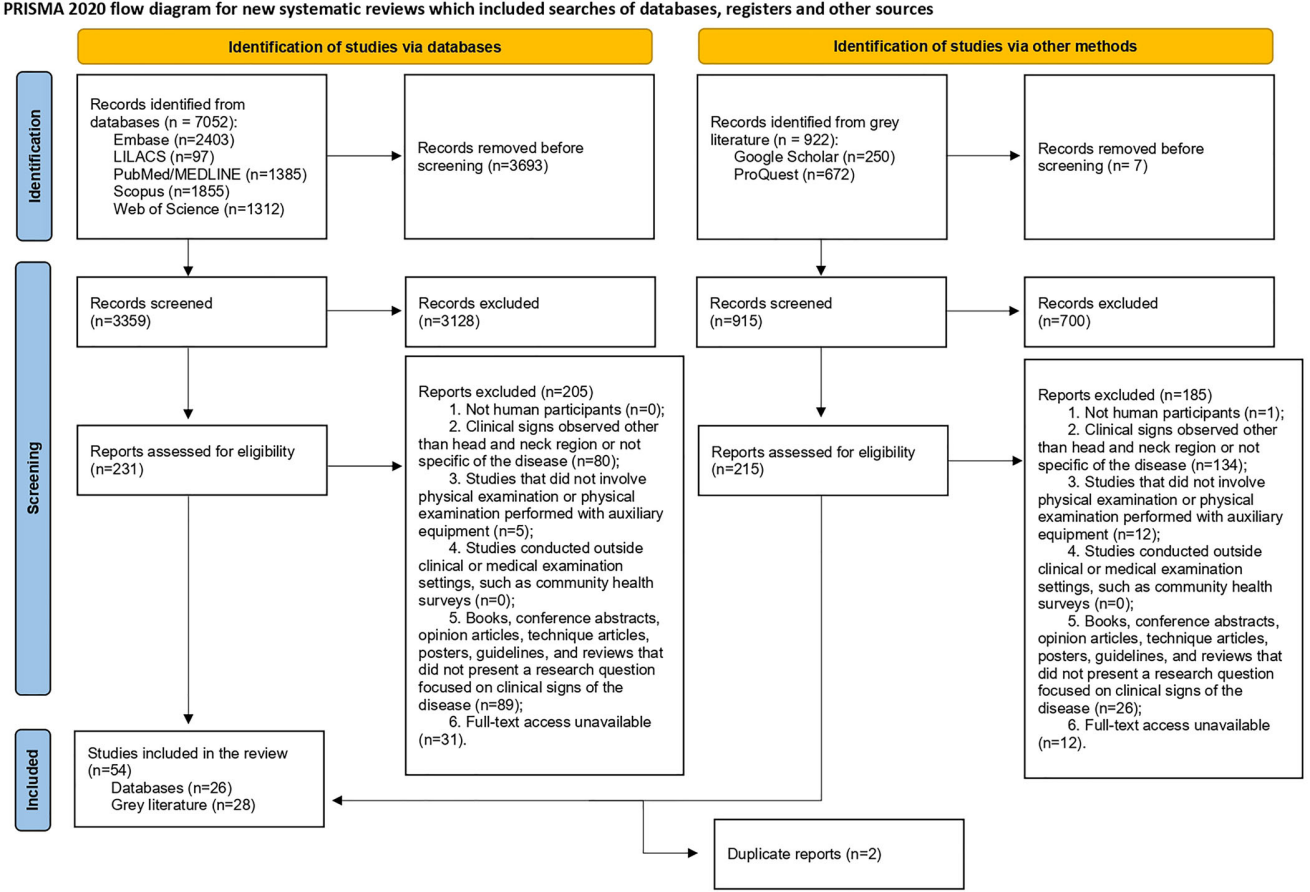
Flow diagram of the literature search and selection criteria adapted from the Preferred Reporting Items for Systematic Reviews and Meta‐Analyses.

A total of 7052 references were initially identified through databases. After removing duplicates (*n* = 3693), 3359 records were screened by titles and abstracts. Of these, 231 full‐text articles were assessed for eligibility, leading to the exclusion of 205 studies for different reasons (Supporting Information 3). Consequently, 26 studies met the inclusion criteria and were included in this review. The search of gray literature retrieved 922 references. After removing duplicates (*n* = 7), 915 records were screened by title and abstract. Of these, 215 articles underwent full‐text review, with 185 subsequently excluded for various reasons (Supporting Information 4). Two additional articles were excluded as they duplicated studies already identified in the main databases. In total, 28 articles from the grey literature were included. Therefore, 54 studies were included in this review. The selection process is shown in Figure 1.

### 3.2 Study Characteristics

**FIGURE 2:**
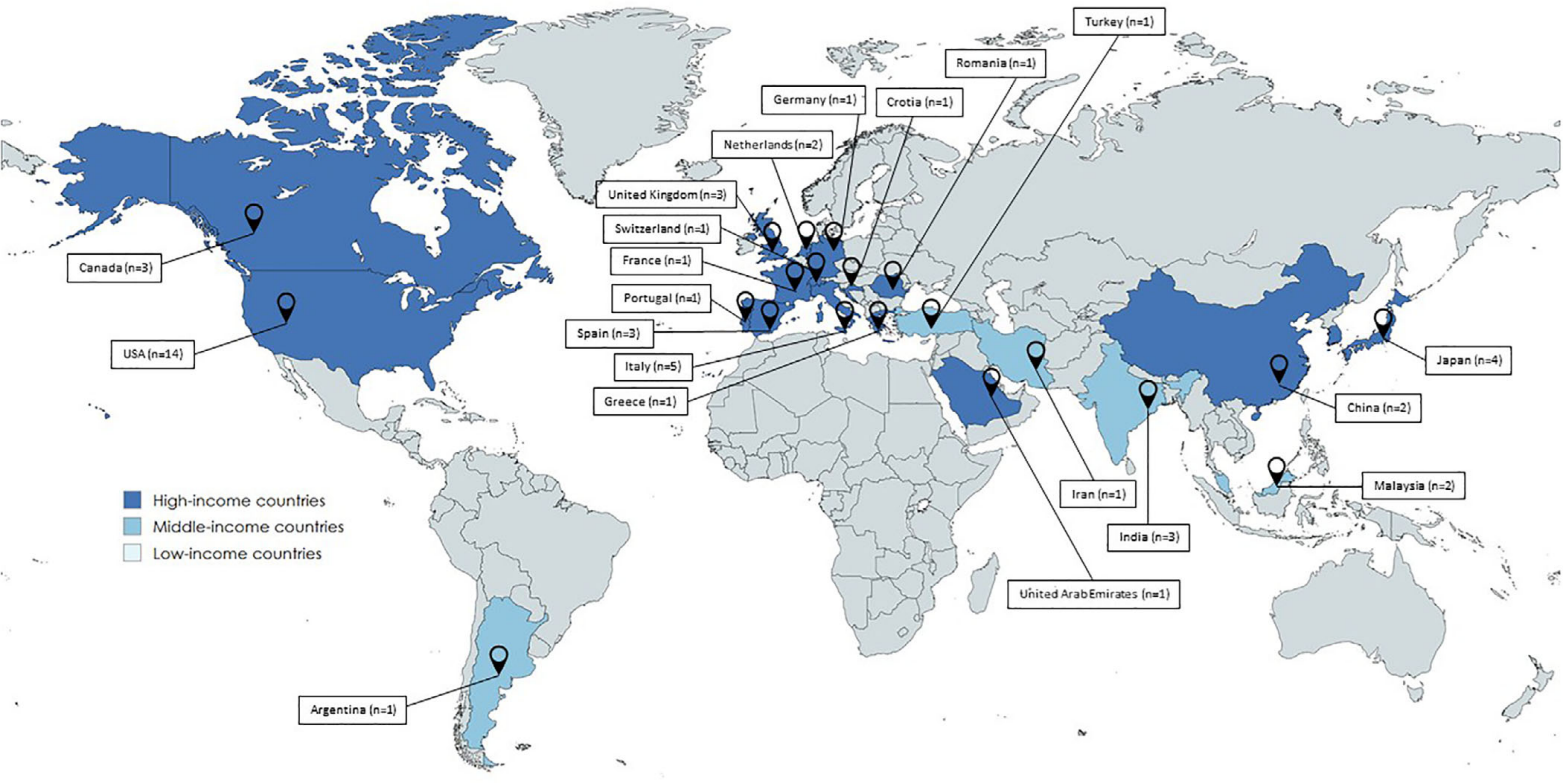
Geographic distribution of the studies included according to the World Bank country income classification.

The included studies were published between 1968 and 2024 and conducted across several countries: United States (*n* = 14 [15, 16, 17, 18, 19, 20, 21, 22, 23, 24, 25, 26, 27, 28]), Italy (*n* = 5 [29, 30, 31, 32, 33], Japan (*n* = 4 [34, 35, 36, 37]), Canada (*n* = 3 [38, 39, 40]), India (*n* = 3 [41, 42, 43]), Spain (*n* = 3 [44, 45, 46]), United Kingdom (*n* = 3 [47, 48, 49]), Malaysia (*n* = 2 [50, 51]), China (*n* = 2 [52, 53]), the Netherlands (*n* = 2 [54, 55]), South Korea (*n* = 2 [56, 57]), Argentina (*n* = 1 [58]), Croatia (*n* = 1 [59]), France (*n* = 1 [60]), Germany (*n* = 1 [61]), Greece (*n* = 1 [62]), Iran (*n* = 1 [63]), Portugal (*n* = 1 [64]), Romania (*n* = 1 [65]), Switzerland (*n* = 1 [66]), Turkey (*n* = 1 [67]), and the United Arab Emirates (*n* = 1 [68]) (Figure 2).

Among the 54 included studies, 28 were case reports and case series [15, 18, 19, 20, 24, 27, 30, 32, 34, 36, 37, 38, 39, 41, 45, 46, 48, 49, 50, 52, 53, 54, 56, 59, 61, 63, 65, 68], 14 were reviews of the literature [16, 25, 26, 29, 35, 40, 42, 44, 47, 51, 57, 60, 62, 64], 7 were cross‐sectional studies [17, 21, 31, 33, 43, 55, 58], and 5 were cohort studies [22, 23, 28, 66, 67].

A total of 43 clinical signs located in the head and neck region were identified. Intraoral signs were the most prevalent (15 signs), followed by cutaneous (10), ocular (9), and auricular signs (3). The remaining signs (*n* = 6) were categorized as “other” due to their unique presentation.

### 3.3 Results of Individual Signs and Studies

#### 3.3.1 Ocular Signs

##### 3.3.1.1 Horner's Syndrome (HS)

Five studies reported Horner's syndrome [28, 48, 49, 56, 59], characterized by the triad of miosis, partial ptosis, and anhidrosis. Alam et al. [48] described a case of metastatic squamous cell carcinoma presenting with Horner's syndrome; Kalantzis et al. [49] attributed it to internal carotid artery dissection, and Kim [56] linked it to stenosis of the distal right vertebral artery. Kovavic et al. [59] reported a case of pleural metastatic breast cancer presenting with HS. Alvi et al. [28] conducted a cohort study with 118 patients with neuroblastoma, ganglioneuroblastoma or esthesioneuroblastoma; among seven with head and neck tumours, three (43%) presented with HS.

##### 3.3.1.2 Coloboma

Three studies reported coloboma [30, 55, 68], a congenital defect involving the absence of ocular tissue such as the iris, eyelids, or optic nerve. This sign was associated with two different diseases, according to three authors: Joubert syndrome [30, 68], with two case reports, and Treacher Collins syndrome [55], with a cross‐sectional study, where 69% (*n* = 41) of the individuals included in the sample presented with this sign.

##### 3.3.1.3 Blue Sclera

Two studies reported blue sclera [31, 33], caused by scleral thinning and choroidal visibility. Colombi et al. [33] and Ritelli et al. [31] linked this sign to Ehlers–Danlos syndrome. Ritelli et al. [31] observed this feature in 31 of 75 patients, while Colombi et al. [33] found it in 52 of 62 patients.

##### 3.3.1.4 Heliotropic Erythema

Two [36, 64] studies discussed heliotropic erythema, characterized as a purplish rash on the upper eyelids, often symmetrical and accompanied by edema. Amorin and Vieira [64] reviewed its association with dermatomyositis, while Ueda‐Hayakawa et al. [36] observed the sign in 3 of 7 patients.

##### 3.3.1.5 Kayser–Fleischer Ring

Two [23, 42] studies identified this sign, a golden‐brown corneal ring reflecting copper deposition, as a sign of Wilson's disease. Das and Ray [42] emphasized its diagnostic significance in their review, and Sternlieb and Scheinberg [23] found the sign in 5 of 17 patients with hepatic copper accumulation confirmed via biopsy.

##### 3.3.1.6 One‐and‐a‐Half Syndrome

Lim et al. [50] reported a case describing this complex neuro‐ophthalmologic sign, characterized by (1) conjugate horizontal gaze palsy (one‐and‐a‐half syndrome; inability to move both eyes horizontally in one direction); (2) ipsilateral internuclear ophthalmoplegia (inability of the eye on the same side of the lesion to adduct move toward the nose), and (3) ipsilateral lower motor neuron‐like facial palsy (weakness of the facial muscles on the same side as the lesion). It is associated with multiple sclerosis.

##### 3.3.1.7 Megalocornea

Megalocornea is an abnormally large cornea (>13 mm), associated with Marfan syndrome in Maumenne's [21] cross‐sectional study, in which 160 patients diagnosed with this syndrome were evaluated. Unfortunately, in this study, the precise measurement of corneal diameter was not performed, owing to its low reproducibility.

##### 3.3.1.8 Sclerocornea

Sclerocornea, is characterized by an opaque cornea resembling the sclera, was associated with HCCS mutations in MLS syndrome, as reported by Rahden et al. [61] in a case‐series, where 5 of the 6 included patients presented with this sign.

##### 3.3.1.9 Supranuclear Gaze Palsy

Difficulty or inability to perform voluntary eye movement impairment with preserved reflexive movements, linked to Niemann‐Pick disease type C, was reviewed by Vanier [60].

#### 3.3.2 Cutaneous Signs

##### 3.3.2.1 Café au Lait Lesions/Macules

These hyperpigmented skin patches were discussed in two reviews. Kanaka‐Gantenbein et al. [62] and Singh et al. [51] reviewed their association with McCune‐Albright syndrome (irregular “coast of Maine” borders) and Neurofibromatosis‐1 (uniform macules). Singh et al. [51] emphasized these macules as a hallmark of McCune‐Albright syndrome.

##### 3.3.2.2 Multiple Facial Trichilemmomas

Two studies identified multiple facial trichilemmomas (flesh‐colored papules) as a marker of Cowden syndrome. Bhanot et al. [15] presented a case report, while Nosé [26] reviewed its diagnostic significance.

##### 3.3.2.3 Pemberton's Sign

Giulea et al. [65] and Keshvani et al. [18] described Pemberton's sign (facial erythema/edema upon arm elevation) in cases of superior vena cava obstruction or syndrome, emphasizing its role in diagnosing vascular compression.

##### 3.3.2.4 Acanthosis Nigricans

Stratakis [25] reviewed its presentation as dark, velvety neck patches associated with Cushing's syndrome.

##### 3.3.2.5 Harlequin Syndrome

Rovner et al. [27] described 15 patients with this syndrome, characterized by asymmetric sweating and flushing on the face and neck. This sign was linked to disrupted sympathetic activity following thoracic epidurals.

##### 3.3.2.6 Lupus Pernio

Lupus pernio, a chronic violet plaque on the nose/cheeks, was associated with sarcoidosis in Aydoğan et al.’s [67].

##### 3.3.2.7 Malar Rash

Shu et al. [34] documented malar rash (butterfly‐shaped facial rash) in 3 siblings with lupus erythematosus.

##### 3.3.2.8 Nasal Tip Necrosis

Stevens and Nielsen [24] reported this rare presentation in lepromatous leprosy (Hansen's disease).

##### 3.3.2.9 Port Wine Sign

Abdolrahimzadeh et al. [29] reviewed the port wine sign, a persistent macular lesion that darkens over time, considered a hallmark of Sturge‐Weber and Klippel‐Trenaunay syndromes. Nodularity or hypertrophy may develop in affected skin.

##### 3.3.2.10 Raspberry‐Like Papillomas

Ghosh et al. [43] reported these mucosal lesions in 6 of 8 patients with Goltz syndrome.

#### 3.3.3 Intraoral Signs

##### 3.3.3.1 Strawberry Tongue

Four studies described strawberry tongue, characterized by a red, swollen tongue with prominent papillae, as a hallmark of Kawasaki disease. Seicshnaydre and Frable [22] reported this sign in 17 of 42 patients, and Su et al. [52] and Yoskovitch et al. [40] emphasized its diagnostic relevance in case reports and reviews. Oh et al. [57] further corroborated its association with the disease.

##### 3.3.3.2 Multiple Neurofibromas

Two studies linked multiple neurofibromas (benign peripheral nerve tumors) to neurofibromatosis type 1 (Von Recklinghausen's disease). D'Ambrosio et al. [17] documented this finding in 27 of 38 patients, and Singh et al. [51] reinforced this association in a review.

##### 3.3.3.3 Multiple Oral Papillomas

Singh et al. [51] and Nosé [26] identified oral papillomas (wart‐like growths on the tongue, gums, and throat) as a key feature of Cowden syndrome.

##### 3.3.3.4 Capillary Malformation of the Lower Lip

González‐Hermosa et al. [46] reported a case of capillary malformation (erythematous‐violaceous macule on the lower lip) associated with CLAPO syndrome (an acronym for Capillary vascular malformation of the lower lip, Lymphatic malformations of the head and neck, facial Asymmetry and Partial or generalized Overgrowth), a rare disorder involving vascular and lymphatic malformations with asymmetric overgrowth.

##### 3.3.3.5 Cocaine‐Induced Midline Destructive Lesions

Cosola et al. [32] described severe intraoral and nasal destruction (palatal perforation, necrotizing ulcers) in 8 patients with cocaine addiction, highlighting the devastating effects of chronic use.

##### 3.3.3.6 False Cheilitis

Gilligan et al. [58] observed fissured commissural papules in 8 of 58 patients with secondary syphilis.

##### 3.3.3.7 Multiple Osteomas

Singh et al. [51] reviewed their association with Gardner's syndrome.

##### 3.3.3.8 Palatal Defect

Knopp et al. [19] linked an orosinusal fistula to granulomatosis with polyangiitis.

##### 3.3.3.9 Perioral Freckling

Singh et al. [51] reviewed perioral freckling (brown‐to‐blue macules on lips/mucosa) as pathognomonic for Peutz–Jeghers syndrome.

##### 3.3.3.10 Recurrent Oral Ulceration

Webb et al. [47] associated painful, scarring oral ulcers with Behçet's disease, noting their widespread distribution on the buccal mucosa, tongue, and palate.

##### 3.3.3.11 Tongue Hamartoma, Multiple Frenula, Cleft Lip/Palate, Upper Lip Notch

Poretti et al. [66] found these signs in 16 patients with oral‐facial‐digital syndrome type VI. Tongue hamartoma, defined as an abnormal proliferation of normal tissues native to the tongue, sometimes forming a nodule or polyp, was found in 13 patients; multiple frenula, variations or duplicated frenulum were present in four patients; cleft lip/palate—defects in which the tissues of the lip and/or palate don't fully fuse during early fetal development, resulting in an opening or gap occurred in five patients; and upper lip notch, a minor or incomplete cleft lip, was present in three patients.

##### 3.3.3.12 Unilateral Cyanosis of the Tongue

Habibzadeh et al. [63] reported this rare sign in a case of lingual artery vasculitis/occlusion.

#### 3.3.4 Auricular Signs

##### 3.3.4.1 Frank's Sign

Three studies identified Frank's sign, a diagonal earlobe crease, clearly visible to the naked eye that extends from the tragus up to the posterior edge of the pinna without continuity. This sign serves as a potential marker of systemic disease. Friedlander et al. [44] reviewed its association with atherosclerosis, while Lin et al. [20] linked it to cardiovascular disease. Notably, Abrahim [38] reported this sign in a patient with polycystic ovary syndrome, suggesting its relevance beyond cardiovascular conditions.

##### 3.3.4.2 Erythematous Lesion of the Retroauricular Skin

Berger et al. [54] documented a painful, progressive erythematous lesion of the retroauricular skin and auricle in a case of Sweet's syndrome, a rare neutrophilic dermatosis often associated with systemic inflammation or malignancy.

##### 3.3.4.3 Milian's Ear Sign

Chakraborty et al. [41] described Milian´s ear sign (tender, erythematous swelling of both ears and face) in a case of erysipelas, emphasizing its diagnostic value for this bacterial skin infection.

#### 3.3.5 Other Signs

##### 3.3.5.1 Erythematous Lesion of the Skin

Cohen and Kurzrock [16] reviewed erythematous, tender plaques/nodules in the head and neck region as a hallmark of Sweet's syndrome, a neutrophilic dermatosis often linked to systemic inflammation or malignancy. This complements Berger et al.’s earlier report of retroauricular lesions in the same condition.

##### 3.3.5.2 Congenital Infiltrating Lipomatosis of the Face

Xu et al. [53] described thickened facial and palatal fat in 18 of 20 patients, underscoring its localized yet progressive nature.

##### 3.3.5.3 Moon Facies

Yamamoto et al. [37] linked facial puffiness edema to Cushing's syndrome in a case report.

##### 3.3.5.4 Septal Perforation

Morales‐Ángulo et al. [45] identified this nasal septal defect in 1 of 25 patients with granulomatosis with polyangiitis, reinforcing its diagnostic value.

##### 3.3.5.5 Tullio's Phenomenon

Suzuki et al. [35] reviewed sound‐induced vertigo/nystagmus as pathognomonic for superior semicircular canal dehiscence syndrome.

##### 3.3.5.6 Unilateral Facial Swelling and Vesicles

Jan et al. [39] documented unilateral facial swelling with vesicles extending to the ear, a classic presentation of herpes zoster (Ramsay Hunt Syndrome).

### 3.4 Qualitative Synthesis

**FIGURE 3:**
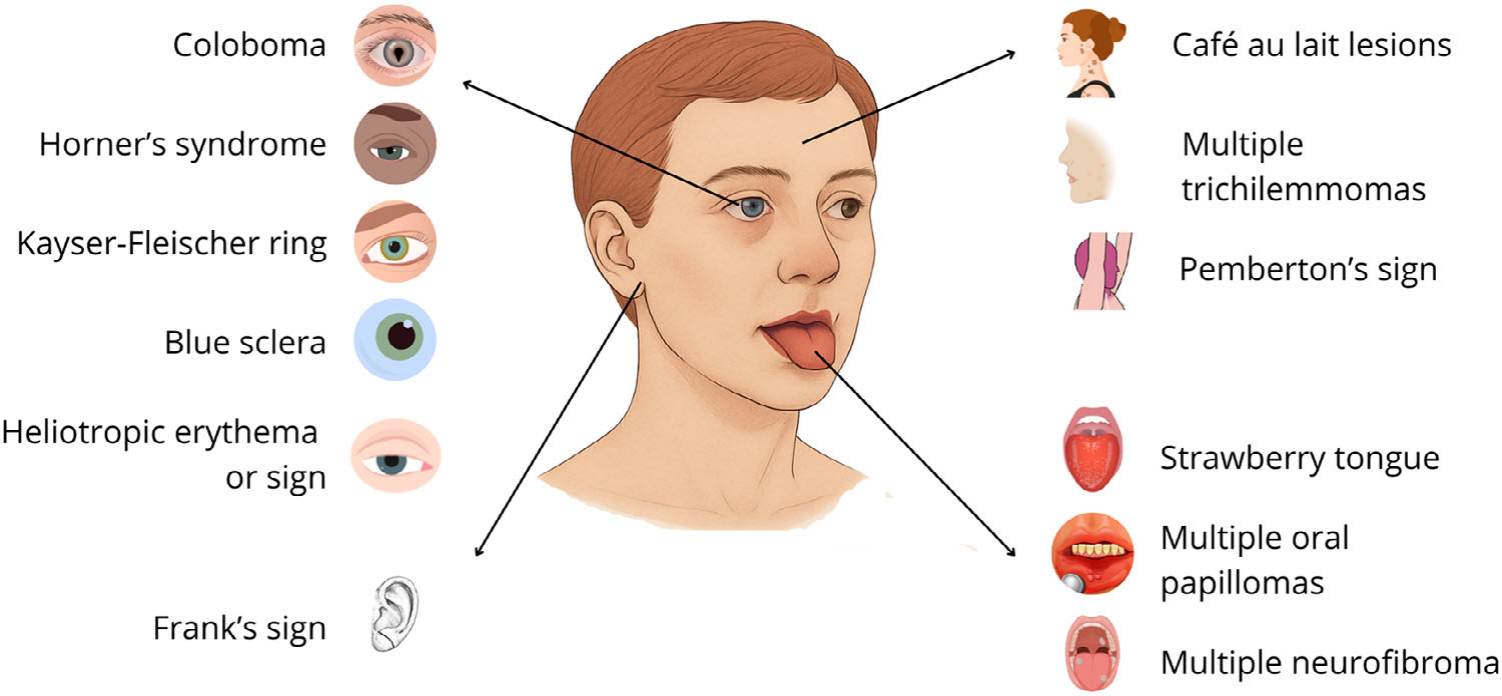
Key clinical signs identified in the head and neck region.

This qualitative synthesis provides an overview of clinical signs in the head and neck region associated with systemic diseases. Key findings include Horner's syndrome, frequently linked to neurological and oncological conditions; café‐au‐lait macules, commonly associated with neurofibromatosis and other genetic syndromes; strawberry tongue, a classic feature of Kawasaki disease; and Frank's sign, which has been correlated with cardiovascular and endocrine disorders. Additionally, rare conditions such as CLAPO syndrome and congenital infiltrating lipomatosis were identified, characterized by vascular and soft tissue abnormalities. Clinical signs were summarized in Table 1, 2, 3, 4, 5 and Figure 3, and the distribution of reported clinical signs according to the World Bank country income classification in Supporting Information 5.

## 4 Discussion

Our synthesis reveals distinct anatomical hotspots for systemic disease markers. Ocular signs, such as Horner's syndrome, often indicate neurological pathology, whereas intraoral findings like the “strawberry tongue” sign tend to suggest infectious or inflammatory etiologies. Cutaneous signs, such as café au lait macules, are key findings in genetic diagnoses, while auricular signs, such as Frank's sign, have been linked to cardiovascular and endocrine pathologies. Despite their diagnostic utility, the heterogeneity in study designs underscores the need for standardized clinical descriptions and reporting.

The clinical signs identified in this review reinforce the diagnostic relevance of findings in the head and neck region, highlighting their potential to indicate underlying systemic diseases. Several manifestations, including Frank's sign, port‐wine stains, Kayser‐Fleischer rings, and strawberry tongue, serve as important diagnostic indicators. These signs can function as *red flags*, encouraging clinicians to pursue targeted diagnostic pathways, such as laboratory testing, genetic screening, or advanced imaging, based solely on physical examination.

However, a key challenge lies in the specificity and sensitivity of these signs. While some signs are highly suggestive of particular diseases, others are nonspecific. For example, oral ulcers may be present in Behçet's disease [47], recurrent aphthous stomatitis, immunodeficiency states, rheumatologic disorders, and skin diseases [69, 70]. Similarly, malar rash, although characteristic of systemic lupus erythematosus, may overlap with rosacea or dermatomyositis, necessitating further investigation and tests for differentiation [71, 72]. These examples underscore the importance of contextual interpretation, in which duration, associated symptoms, patient history, and coexisting clinical signs must be integrated into the diagnostic process.

The eyes, as extensions of the central nervous system, provide early and precise diagnostic insights, particularly in the context of neurological and metabolic diseases. For instance, Kayser‐Fleischer rings, caused by copper deposition in Descemet's membrane of the cornea, are strongly associated with Wilson's disease and may precede hepatic or neurological symptoms. These rings are present in approximately 95% of neurologically symptomatic patients and in over 50% of asymptomatic individuals [73, 74]. Similarly, neuro‐ophthalmic syndromes such as the One‐and‐a‐half syndrome, characterized by conjugate gaze palsy with ipsilateral facial nerve involvement, can be early indicators of demyelinating conditions like multiple sclerosis [50]. The literature suggests cerebrovascular disease is the most common cause (about 63%), followed by demyelination [75]. Horner's syndrome, though associated with various diseases, consistently indicates disruption of the sympathetic pathway to one side of the face [28, 48, 49, 56, 59]. These ocular signs thus serve both as localizing and etiological diagnostic tools.

The oral cavity serves as an important diagnostic landscape for haematological, infectious, autoimmune, and genetic disorders. Recurrent painful oral ulcers are characteristic of Behçet's disease and, when accompanied by genital ulcers and ocular inflammation, support a definitive diagnosis [47, 76, 77]. However, similar oral ulcers can occur in other diseases, requiring careful evaluation of duration, patient history, and systemic symptoms [69, 70]. Strawberry tongue, characterized by a red, swollen, and papillated appearance, is another notable sign, commonly observed in Kawasaki disease [22, 40, 52, 57]. These examples underscore the diagnostic importance of the oral cavity in systemic disease evaluation.

Auricular findings, though less commonly emphasized, are emerging as clinically significant. Frank's sign has been investigated as a potential non‐invasive marker for cardiovascular disease, particularly in younger patients without traditional cardiovascular risk factors [8]. Additionally, Milian's ear sign, characterized by erythema of the ears, supports distinguishing erysipelas from other superficial infections, such as cellulitis [78].

Facial and cutaneous manifestations also play a bridging role between dermatology and systemic disease. Malar rash is classically seen in systemic lupus erythematosus and correlates with disease activity and photosensitivity. However, its resemblance to rosacea or dermatomyositis may require further investigation and additional tests for differentiation [71, 72]. Café au lait macules, depending on their morphology, can suggest distinct syndromic associations: lesions with smooth borders are linked to Neurofibromatosis‐1, while irregular‐bordered macules are more commonly associated with McCune‐Albright syndrome [62]. Such visible manifestations not only contribute to diagnostic suspicion but also reflect chronic disease burden.

An important contribution of this review was the identification of rare but highly specific signs, often observed in syndromic or genetic disorders. These include lupus pernio, megalocornea, perioral freckling, and moon facies. These signs reinforce the role of the physical examination in early syndrome recognition, especially in pediatric patients or in adults with undiagnosed conditions.

Geographic and socioeconomic factors may influence both the prevalence and recognition of head and neck clinical signs. Variability in healthcare access, diagnostic resources, and clinician training between high‐income and low‐income countries may affect early detection of systemic diseases [79]. In the present review, most publications originated from high‐income countries, which likely reflects the greater research output from these regions rather than true geographic differences in the occurrence of clinical signs. In addition, several studies described multiple clinical signs within a single report, which may artificially increase the frequency of signs when aggregated. Additionally, certain cutaneous manifestations—such as erythema, malar rash, or subtle pigmentation changes—may be more difficult to recognize in individuals with darker skin phototypes, potentially contributing to delayed diagnosis [80, 81].

Many of the identified signs are not pathognomonic and may overlap across multiple conditions. For example, oral ulcers may occur in Behçet's disease, recurrent aphthous stomatitis, systemic lupus erythematosus, and hematologic disorders [82]. Similarly, facial flushing may be associated with endocrine, infectious, or autoimmune etiologies [83]. Therefore, these signs should be interpreted within a comprehensive clinical context, including patient history, associated systemic manifestations, and complementary investigations [84, 85].

However, this scoping review has limitations that must be acknowledged. Despite a comprehensive search strategy, it is challenging to include every possible medical descriptor in such a broad field. Consequently, some studies may not have been captured if their descriptors, such as those describing “moon facies” (Cushing's syndrome), “palatal perforations” (cocaine use), or “Frank's sign” (cardiovascular disease), were not explicitly included in the search. Most included studies originated from high‐income countries, which likely reflects differences in research output rather than true geographic variation in the occurrence of clinical signs. Additionally, several studies reported multiple signs within a single publication, which may artificially increase frequency counts. Incomplete reporting of demographic data limited the ability to perform comparative analyses, highlighting a relevant area for future research. This inherent challenge is a recognized limitation of scoping reviews and should be considered when interpreting the results. The majority of the included studies were case reports and case series, limiting generalizability due to small sample sizes and potential publication bias. Considerable heterogeneity in the reporting and description of clinical signs was observed, and interobserver reliability or reproducibility was not evaluated. Moreover, this review focused on qualitative synthesis without performing meta‐analysis or quantitative assessment of diagnostic value.

In summary, this scoping review identified a diverse range of clinical signs located in the head and neck region that are associated with systemic diseases. Intraoral signs were the most frequently reported, comprising 15 distinct findings, followed by cutaneous (10), ocular (9), and auricular signs (3). An additional six signs that did not fall within these anatomical categories were grouped separately. Notable findings included pathognomonic or highly suggestive signs such as strawberry tongue (Kawasaki disease), café‐au‐lait macules (neurofibromatosis), Kayser‐Fleischer rings (Wilson's disease), and Frank's sign (cardiovascular disorders). The predominance of rare or isolated reports highlights the need for greater clinical awareness and further research to validate these associations and explore their diagnostic value in more diverse populations.

## 5 Conclusion

This scoping review systematically mapped key clinical signs in the head and neck region that can be observed during physical examination, many of which are associated with systemic diseases. A total of 43 distinct signs were identified and categorized anatomically. Intraoral signs were the most frequently reported, followed by cutaneous, ocular, and auricular findings. Notable examples include strawberry tongue (Kawasaki disease), café‐au‐lait macules (neurofibromatosis), malar rash (systemic lupus erythematosus), Kayser‐Fleischer rings (Wilson's disease), moon facies (Cushing's syndrome), septal perforation (Granulomatosis with polyangiitis and cocaine abuse), and Frank's sign (cardiovascular and endocrine disorders). These signs offer accessible, non‐invasive, and cost‐effective diagnostic clues that can guide further investigation and management, they serve as potential indicators of a wide range of systemic diseases, including genetic, infectious, autoimmune, metabolic, and oncologic conditions.

Clinicians should maintain a high index of suspicion when encountering head and neck signs. Creating clinical checklists or integrating visual sign recognition into electronic health records could facilitate earlier detection of systemic diseases. Moreover, interdisciplinary collaboration can enhance the diagnostic yield of these physical findings.

## Funding

This research did not receive any specific grant from funding agencies in the public, commercial, or non‐profit sectors.

## Conflicts of Interest

The authors have no relevant financial or non‐financial interests to disclose.

## Supporting information




**Supplementary Material 1**: Search Strategy of all Databases and Grey Literature.

**Supplementary Material 2**: Summary of the Descriptive Features of the 54 Screening Studies Included in the Scoping Review.

**Supplementary Material 3**: Excluded Studies and Exclusion Criteria From Databases.

**Supplementary Material 4**: Excluded Studies and Exclusion Criteria From Grey Literature.

**Supplementary Material 5**: Distribution of Reported Clinical Signs According to the World Bank Country Income Classification.
