# Supplementary material for: Head and Neck Clinical Signs Associated With Diseases: A Scoping Review
Source: Spec Care Dentist. 2026 May 14;46:e70185. doi: 10.1111/scd.70185 (PMC13176508; doi:10.1111/scd.70185)
Supplement: Supplementary file 1 — Supplementary Material 1: Search Strategy of all Databases and Grey Literature. [file SCD-46-0-s004.docx]

**Supplementary material S1 – Search strategy of all databases and grey literature**

| **DATABASE** | **SEARCH STRATEGY** | **RESULTS** |
| --- | --- | --- |
| MEDLINE/PubMed | ("head and neck"[Title/Abstract]) AND ("sign"[Title/Abstract] OR "signs"[Title/Abstract] OR "clinical examination"[Title/Abstract] OR "physical examination"[Title/Abstract] OR "skin manifestations"[Title/Abstract] OR "skin rash"[Title/Abstract] OR "phenomenon"[Title/Abstract] OR "odors"[Title/Abstract] OR "smell"[Title/Abstract]) AND ("disease"[Title/Abstract] OR "syndrome"[Title/Abstract]) | 1385 |
| EMBASE | ('disease':ti,ab OR 'syndrome':ti,ab) AND ('sign':ti,ab OR 'signs':ti,ab OR 'clinical examination':ti,ab OR 'physical examination':ti,ab OR 'skin manifestation':ti,ab OR 'skin rash':ti,ab OR 'phenomenon':ti,ab OR 'odor':ti,ab OR 'smell':ti,ab) AND ('head and neck':ti,ab) | 2403 |
| LILACS | ("disease" OR "doença" OR "enfermedad" OR "síndrome" OR "syndrome") AND ("sign" OR "sinal" OR "signo" OR "clinical examination" OR "exame clínico" OR "examen clínico" OR "physical examination" OR "exame físico" OR "examen físico" OR "skin manifestations" OR "manifestações cutâneas" OR "manifestaciones cutáneas" OR "skin rash" OR "erupção cutânea" OR "erupciones cutáneas" OR "odors" OR "odores" OR "olores" OR "smell" OR "cheiro" OR "phenomenon" OR "fenômeno" OR "fenómeno") AND ("head and neck" OR "cabeça e pescoço" OR "cabeza y cuello") | 97 |
| SCOPUS | TITLE-ABS ("disease" OR "syndrome") AND TITLE-ABS ("sign" OR "signs" OR "clinical examination" OR "physical examination" OR "skin manifestations" OR "skin rash" OR "odors" OR "smell" OR "phenomenon") AND TITLE-ABS ("head and neck") | 1855 |
| WEB OF SCIENCE | TS=("disease" OR "syndrome") AND TS=("sign" OR "signs" OR "clinical examination" OR "physical examination" OR "skin manifestations" OR "skin rash" OR "odors" OR "smell" OR "phenomenon") AND TS=("head and neck") | 1312 |
|  |  | TOTAL = 7052 |
| ProQuest | TIAB("head and neck" OR "facial") AND TIAB("sign" OR "skin manifestations" OR "skin rash" OR "odors" OR "smell" OR "phenomenon") AND TIAB("disease" OR "syndrome") | 672 |
| Google Scholar | ("disease" OR "doença" OR "enfermedad" OR "síndrome" OR "syndrome") AND ("sign" OR "sinal" OR "signo" OR "clinical examination" OR "exame clínico" OR "examen clínico" OR "physical examination" OR "exame físico" OR "examen físico" OR "skin manifestations" OR "manifestações cutâneas" OR "manifestaciones cutáneas" OR "skin rash" OR "erupção cutânea" OR "erupciones cutáneas" OR "odors" OR "odores" OR "olores" OR "smell" OR "phenomenon" OR "fenômeno" OR "fenómeno") AND ("head and neck" OR "cabeça e pescoço" OR "cabeza y cuello") | 250 |
|  |  | TOTAL = 922 |
