# Supplementary material for: Head and Neck Clinical Signs Associated With Diseases: A Scoping Review
Source: Spec Care Dentist. 2026 May 14;46:e70185. doi: 10.1111/scd.70185 (PMC13176508; doi:10.1111/scd.70185)
Supplement: Supplementary file 2 — Supplementary Material 2: Summary of the Descriptive Features of the 54 Screening Studies Included in the Scoping Review. [file SCD-46-0-s002.docx]

**Supplementary material S2 – Summary of the descriptive features of the 54 screening studies included in the scoping review**

| **Author (year) country** | **Study design (sample size/patients with sign)** | **Clinical sign** | **Main characteristics** | **Associated with (disease)** |
| --- | --- | --- | --- | --- |
| Lim et al. (2023), Malaysia | Case report | One-and-a-half syndrome | Combination of symptoms: 1) conjugate horizontal gaze palsy (one-and-a-half syndrome; inability to move both eyes horizontally in one direction); 2) ipsilateral internuclear ophthalmoplegia inability of the eye on the same side of the lesion to adduct move towards the nose), and 3) ipsilateral lower motor neuron-like facial palsy (weakness of the facial muscles on the same side as the lesion). | Multiple sclerosis |
| Colombi et al. (2017), Italy | Cross-sectional (62/52) | Blue sclera | Sclera takes on a bluish color, due to a thinning of the sclera, allowing the color of the underlying tissue (choroid) to be visible. | Ehlers-Danlos syndrome |
| Chettiankandi, Khan & Khan (2022), United Arab Emirates | Case report | Coloboma | Lack of tissue during fetal development, resulting in visible defects in the eye structures like the iris, eyelids, or optic nerve. | Joubert syndrome |
| Marres et al. (1996), England | Cross-sectional (59/41) | Coloboma | Lack of tissue during fetal development, resulting in visible defects in the eye structures like the iris, eyelids, or optic nerve. | Treacher Collins syndrome |
| Niceta et al. (2020), Italy | Case report and review | Coloboma | Lack of tissue during fetal development, resulting in visible defects in the eye structures like the iris, eyelids, or optic nerve. | Joubert syndrome |
| Amorin & Vieira (2012), Portugal | Review | Heliotropic erythema | Purplish rash of the upper eyelids, with symmetrical distribution, and may be accompanied by edema. | Dermatomyosis |
| Ueda-Hayakawa et al. (2018), Japan | Case-series (7/3) | Heliotropic sign | Purplish rash of the upper eyelids, with symmetrical distribution, and may be accompanied by edema. | Dermatomyosis |
| Alam et al. (2016), England | Case report | Horner's syndrome | Triad of miosis, partial ptosis and loss of hemifacial sweating. | Metastatic squamous cell carcinoma in the tonsil |
| Alvi et al. (2017), USA | Cohort | Horner's syndrome | Triad of miosis, partial ptosis and loss of hemifacial sweating. | Neuroblastoma, ganglioneuroblastoma or esthesioneuroblastoma |
| Kalantzis et al. (2014), UK | Case report | Horner's syndrome | Triad of miosis, partial ptosis and loss of hemifacial sweating. | Internal carotid artery dissection |
| Kim (2013), Korea | Case report | Horner's syndrome | Triad of miosis, partial ptosis and loss of hemifacial sweating. | Stenosis of the distal part of the right vertebral artery |
| Kovacic et al, (2007), Croatia | Case report | Horner's syndrome | Triad of miosis, partial ptosis and loss of hemifacial sweating. | Metastatic breast cancer |
| Das & Ray (2006), India | Review | Kayser-Fleischer ring | Golden-brown ring that appears around the edge of the cornea, that reflects copper deposition on the brain. | Wilson's disease |
| Sternlieb & Scheinberg (1968), USA | Cohort | Kayser-Fleischer ring | Golden-brown ring that appears around the edge of the cornea, that reflects copper deposition on the brain. | Wilson's disease |
| Ritelli et al. (2020), Italy | Cross-sectional (75/31) | Light blue sclera | Sclera takes on a bluish color, due to a thinning of the sclera, allowing the color of the underlying tissue (choroid) to be visible. | Ehlers-Danlos syndrome |
| Maumenne (1981), USA | Cross-sectional (160/NA) | Megalocornea | Abnormally large cornea (>13 mm) | Marfan syndrome |
| Rahden et al. (2014), Germany | Case-series (6/5) | Sclerocornea | The cornea is opaque and resembles the sclera. | HCCS mutations with MLS syndrome |
| Vanier (2010), France | Review | Supranuclear gaze palsy | Difficulty or inability to perform voluntary eye movements, while reflex eye movements remain normal. | Niemann-Pic disease type C |
| Stratakis (2016), USA | Review | Acantose nigricans | Skin condition characterized by dark, thick, velvety patches, usually in the folds of the body, such as the neck | Chushing's syndrome |
| Kanaka-Gantenbein et al. (2016), Greece | Review | Café au lait lesions | Uniformly hyperpigmented macules (Neurofibromatosis-1) and irregular bordes, also called "Coast of Maine" borders (McCune-Albright syndrome). | Neurofibromatosis-1 and McCune-Albright syndrome |
| Singh et al. (2019), Malaysia | Review | Café au lait macules | Hyperpigmented macules. | McCune–Albright Syndrome |
| Rovner et al. (2019), USA | Case-series (15) | Harlequin syndrome | Asymmetric sweating and flushing on the neck and face. | Disruption of vasomotor and sudomotor sympathetic activity after thoracic epidurals |
| Aydoğan et al. (2013), Turkey | Cohort (27/4) | Lupus pernio | Chronic, reddish to violet, hardened plaques that can be found mainly on the nose, cheeks, ears. | Sarcoidosis |
| Shu et al. (2012), Japan | Case reports (3/3) | Malar rash | Rash that appears on the face, covering the cheeks and the tip of the nose, and has butterfly appearance due to its shape | Lupus erythematous |
| Bhanot, Harrell & Levin (2023), USA | Case report | Multiple facial trichilemmomas | Flesh-colored papules on the face. | Cowden syndrome |
| Nosé (2016), USA | Review | Multiple facial trichilemmomas | Flesh-colored papules on the face. | Cowden syndrome |
| Stevens & Nielsen (1981), USA | Case report | Nasal tip necrosis | Nasal tip necrosis. | Hansen's disease (lepromatous leprosy) |
| Giulea et al. (2016), Romenia | Case report | Pemberton's sign | Facial erythema and edema when arms elevated above the head. | Superior vena cava syndrome |
| Keshvani, Yek & Johnson (2018), USA | Case report | Pemberton's sign | Facial erythema and edema when arms elevated above the head. | Superior vena cava obstruction |
| Abdolrahimzadeh et al. (2015), Italy | Review | Port wine sign | Well-defined macular lesion initially pink, with a smooth surface that, unlike hemangiomas, partially blanches with pressure. The lesion develops proportionally with the child and usually gets darker in color. The skin over the port wine sign can present nodularity or hypertrophy. | Sturge-Weber Syndrome and Klippel-Trenaunay Syndrome |
| Ghosh et al. (2017), India | Cross-sectional (8/6) | Raspberry-like papilomas | Soft, pedunculated growths with numerous finger-like projections, often resembling a raspberry, typically appear at junctions between skin and mucous membranes, such as the lips, perioral region, and around the eyes. | Goltz syndrome |
| González-Hermosa et al. (2019), Spain | Case report | Capillary malformation of the lower lip | Erythematous-violaceous macule of the lower lip | CLAPO syndrome (Capillary vascular malformation of the lower lip, Lymphatic mal- formations of the head and neck, Asymmetry and Partial or generalized Overgrowth) |
| Cosola et al. (2021), Italy | Case-series (8) | Cocaine-induced midline destructive lesions | Ranges from intranasal crusting, foul exudate, epistaxis, nasal scabs, saddle nose deformities, nasocutaneous fistulas, necrotizing ulcerative lesions, and septal perforation. In more severe cases, the destruction extends to the middle and superior turbinates, the lateral wall of the nose, and the hard palate. | Cocaine addiction |
| Gilligan et al. (2021), Argentina | Cross-sectional (58/8) | False cheilitis | Unilateral, slightly elevated pattern and present as a fissured papule of the commissure. | Secondary syphilis |
| D’Ambrosio, Langlais & Young (1988), USA | Cross-sectional (38/27) | Multiple neurofibroma | Benign tumors, which grow in peripheral nerves. | Neurofibromatois type 1 |
| Singh et al. (2019), Malaysia | Review | Multiple neurofibroma | Benign tumors, which grow in peripheral nerves. | Neurofibromatois type 1 or Von Recklinghausen’s neurofibromatosis |
| Singh et al. (2019), Malaysia | Review | Multiple osteomas | Numerous benign bone tumors. | Gardner's syndrome |
| Singh et al. (2019), Malaysia | Review | Multiple papillomatous nodules | Wart-like bumps on the tongue, gums, back of the throat, and tonsils. | Cowden syndrome |
| Nosé (2016), USA | Review | Oral papillomas | Wart-like bumps on the tongue, gums, back of the throat, and tonsils. | Cowden syndrome |
| Knopp, Baran & Casey (2023), USA | Case report | Palatal defect | Defect on hard and/or soft palate, causing buconasal fistula | Granulomatosis with Polyangiitis |
| Singh et al. (2019), Malaysia | Review | Perioral frecklin | Brown to blue–gray macules primarily affect the vermilion zone, the labial and buccal mucosa, and the tongue. | Peutz–Jeghers syndrome |
| Webb, Moots & Swift (2008), UK | Review | Recurrent oral ulceration | Multiple ulcers, of variable size, occur extensively on the buccal membrane, tongue, palate and in the oropharynx, classically painful, surrounded by erythema and the larger ones heal with scarring. | Behçet’s disease |
| Oh, Cho & Choi (2023), Republic of Korea | Review | Strawberry tongue | Red, swollen tongue with prominent bumps | Kawasaki disease |
| Seicshnaydre & Frable (1993), USA | Cohort (42/17) | Strawberry tongue | Red, swollen tongue with prominent bumps | Kawasaki disease |
| Su et al. (2022), China | Case report and review | Strawberry tongue | Red, swollen tongue with prominent bumps | Kawasaki disease |
| Yoskovitch et al. (2000), Canada | Review | Strawberry tongue | Red, swollen tongue with prominent bumps | Kawasaki disease |
| Poretti et al. (2012), Switzerland | Cohort (16/13, 4, 5, 3) | Tongue hamartoma, multiple frenula, cleft lip/palate, upper lip notch | Tongue hamartoma (abnormal arrangement of normal tissues native to the tongue, sometimes forming a nodule or polyp); multiple frenula (variations or double frenulum); cleft lip/palate (defects where the tissues in the lip and/or palate don't fully fuse together during early pregnancy, resulting in an opening or gap); upper lip notch (minor or incomplete cleft lip). | Oral-Facial-Digital Syndrome Type VI |
| Habibzadeh et al. (2021), Iran | Case report | Unilateral cyanosis of the tongue | Discoloration of the left half of the tongue. | Vasculitis or occlusion of lingual artery |
| Berger et al. (2021), Netherlands | Case report | Erythematous lesion of the retroauricular skin | Protruding, mildly bulging and progressive erythematous lesion of the retroauricular skin and the auricula, painful when palpated. | Sweet's syndrome (acute febrile neutrophilic dermatosis) |
| Abrahim (2021), Canada | Case report | Frank sign | Diagonal earlobe creases. | Polycystic ovary syndrome |
| Friedlander, López-López & Velasco-Ortega (2012), Spain | Review | Frank sign | Diagonal earlobe creases. | Atherosclerosis |
| Lin et al. (2018), USA | Case report | Frank sign | Diagonal earlobe creases. | Cardiovascular disease |
| Chakraborty, Bhanja & Sil (2021), India | Case report | Milian ear sign | Well-demarcated tender indurated, erythematous, and edematous swelling of both ears and face. | Erysipela |
| Cohen & Kurzrock (1987), USA | Review | Erythematous lesion of the skin | Elevated, tender plaques and nodules in the head and neck area. | Sweet's syndrome (acute febrile neutrophilic dermatosis) |
| Xu et al. (2024), China | Case series (20/18) | Congenital infiltrating lipomatosis of the face | Thickened buccal subcutaneous and palatal submucosa fat | Congenital infiltrating lipomatosis |
| Yanamoto et al. (2021), Japan | Case report | Moon face | Swollen, round, or puffy face. | Cushing's syndrome |
| Morales-Ângulo et al. (2012), Spain | Case-series (25/1) | Septal perforation | Hole in the cartilage or bone that separates the two nostrils. | Granulomatosis with polyangiitis |
| Suzuki et al. (2024), Japan | Review | Tullio's phenomenon | Sound-induced dizziness, vertigo, or nystagmus (rapid eye movements). | Superior canal dehiscence syndrome |
| Jan et al. (2006), Canada | Case report | Unilateral facial swelling and vesicles | Facial swelling centred in the right submandibular areaextending to the cheek and parotid regions with multiple erythematous, crusted vesicular eruptions, extending to the ipsilateral tragus, helix and pinna of the ear. | Ramsey Hunt syndrome |
