# Supplementary material for: Head and Neck Clinical Signs Associated With Diseases: A Scoping Review
Source: Spec Care Dentist. 2026 May 14;46:e70185. doi: 10.1111/scd.70185 (PMC13176508; doi:10.1111/scd.70185)
Supplement: Supplementary file 3 — Supplementary Material 3: Excluded Studies and Exclusion Criteria From Databases. [file SCD-46-0-s003.docx]

**Supplementary Material 3**. Excluded studies and exclusion criteria from databases.

| **Reference** | **Author, year** | **Reason for exclusion*** |
| --- | --- | --- |
|  | (Abdolrahimzadeh et al., 2016) | 3 |
|  | (Abudinen-Vasquez & Marin, 2020) | 2 |
|  | (Agarwal et al., 2013) | 3 |
|  | (Ipek et al., 2015) | 5 |
|  | (Ahmed et al., 2018) | 2 |
|  | (Ahmetgjekaj et al., 2021) | 2 |
|  | (Aktan, 2012) | 2 |
|  | (Alfonso & Centelles, 2016) | 2 |
|  | (Alkali et al., 2009) | 5 |
|  | (Allegrini et al., 2016) | 2 |
|  | (AlOtaibi et al., 2025) | 2 |
|  | (Arcangeli & Brunelli, 2015) | 5 |
|  | (Arshad et al., 2022) | 2 |
|  | (Ashimori et al., 2003) | 2 |
|  | (Askari et al., 2024) | 6 |
|  | (Asmus et al., 2009) | 2 |
|  | (Baiocco et al., 1984) | 2 |
|  | (Bakhous et al., 2015) | 5 |
|  | (Bakhshaee et al., 2014) | 2 |
|  | (Bandla et al., 2022) | 5 |
|  | (Bardan & Hata, 2007) | 5 |
|  | (Barrett, 2012) | 2 |
|  | (Berisavac et al., 2009) | 5 |
|  | (Berth-Jones, 1996) | 2 |
|  | (Bhidayasiri & Tarsy, 2012) | 5 |
|  | (Binenbaum et al., 2014) | 5 |
|  | (Bonometti et al., 2021) | 5 |
|  | (Borrue-Fernandez & Gomez-Raposo, 2011) | 5 |
|  | (Boukovalas et al., 2019) | 2 |
|  | (Bumsted, 1980) | 5 |
|  | (Calderon-Castrat et al., 2017) | 5 |
|  | (Callen, 1985) | 2 |
|  | (Calzadilla & Zuleta, 2015) | 5 |
|  | (Capodiferro et al., 2021) | 5 |
|  | (H. C. Chen et al., 2007) | 2 |
|  | (Chen et al., 2023) | 2 |
|  | (Chong, 2015) | 5 |
|  | (Christen et al., 1993) | 2 |
|  | (Chung et al., 2022) | 2 |
|  | (Coleman et al., 2020) | 2 |
|  | (Contrucci & Martin, 2015) | 2 |
|  | (Cooper et al., 2014) | 5 |
|  | (Cutright et al., 1977) | 2 |
|  | (Dede et al., 2011) | 5 |
|  | (Delgado Martínez et al., 1989) | 6 |
|  | (Dickstein et al., 2018) | 5 |
|  | (Dogra et al., 2005) | 2 |
|  | (Dorsch, 2014) | 2 |
|  | (Dunphy et al., 2011) | 5 |
|  | (Çello et al., 2013) | 5 |
|  | (Elshafei, 2012) | 5 |
|  | (Eroz et al., 2017) | 2 |
|  | (Fieux et al., 2020) | 5 |
|  | (Fogo et al., 2013) | 5 |
|  | (Forbes et al., 2009) | 5 |
|  | (Gadalla & Kulkarni, 2021) | 5 |
|  | (Gandia et al., 1993) | 2 |
|  | (Gilio et al., 2018) | 5 |
|  | (GÃ¼leÃ§ et al., 2007) | 6 |
|  | (Gomes et al., 2007) | 2 |
|  | (Gordon & Wain, 2009) | 5 |
|  | (Gouda et al., 2022) | 2 |
|  | (Habib & Raza, 2012) | 2 |
|  | (Haebich et al., 2019) | 5 |
|  | (Hafi et al., 2019) | 2 |
|  | (Handa & Dogra, 2003) | 2 |
|  | (Hannah-Shmouni & Sirrs, 2015) | 5 |
|  | (Harris & Weisman, 2007) | 5 |
|  | (Hassan et al., 2017) | 5 |
|  | (Hilal, 1994) | 6 |
|  | (Htwe, 2020) | 5 |
|  | (Iijima et al., 2024) | 2 |
|  | (Isaacson et al., 2010) | 2 |
|  | (Jacobs et al., 1979) | 6 |
|  | (Jiang et al., 2017) | 2 |
|  | (Karam & Hahr, 2019) | 5 |
|  | (Kasem Ali Sliman et al., 2023) | 3 |
|  | (Kaushik et al., 2004) | 2 |
|  | (Khatri et al., 2012) | 5 |
|  | (M. K. Kim & Alvi, 1999) | 2 |
|  | (Y. S. Kim & Lee, 2009) | 6 |
|  | (Ko et al., 2015) | 2 |
|  | (Koch et al., 1978) | 6 |
|  | (Kozicky et al., 2018) | 5 |
|  | (Krause et al., 2001) | 2 |
|  | (Kumar et al., 2021) | 5 |
|  | (Kurtz et al., 1990) | 5 |
|  | (Lai et al., 2023) | 5 |
|  | (Lee, 2018) | 5 |
|  | (W. M. Leung et al., 2005) | 2 |
|  | (A. K. C. Leung et al., 2021) | 6 |
|  | (Lindsay et al., 2003) | 2 |
|  | (LITTLE, 1946) | 2 |
|  | (Liu et al., 2024) | 6 |
|  | (Louka et al., 2023) | 5 |
|  | (Maceri, 1986) | 6 |
|  | (Mahtta et al., 2018) | 5 |
|  | (Malavika et al., 2023) | 5 |
|  | (Maniglia et al., 1996) | 6 |
|  | (Martin et al., 2020) | 5 |
|  | (Martinez et al., 2014) | 5 |
|  | (Masele et al., 2020) | 3 |
|  | (Matsuo et al., 1998) | 6 |
|  | (McCusker et al., 2024) | 5 |
|  | (McNeilly & Wilkerson, 2022) | 2 |
|  | (Mehta et al., 2018) | 5 |
|  | (Mengen et al., 2017) | 5 |
|  | (Moazzez & Alvi, 1998) | 2 |
|  | (Mohammed & Al-Gadi, 2003) | 6 |
|  | (Mohindra et al., 2015) | 5 |
|  | (Mon & Nakamura, 2000) | 6 |
|  | (Moroco & McGinn, 2018) | 5 |
|  | (Mozumder et al., 2022) | 5 |
|  | (Muecke & Amedee, 1994) | 6 |
|  | (Mumtaz et al., 2018) | 5 |
|  | (Murakawa et al., 1996) | 2 |
|  | (Nair et al., 2014) | 5 |
|  | (Nuta & Puianu, 2019) | 5 |
|  | (Pan et al., 2017) | 5 |
|  | (Park et al., 2012) | 2 |
|  | (Parris et al., 1987) | 2 |
|  | (Pellitteri, 2007) | 5 |
|  | (Picardo et al., n.d.) | 2 |
|  | (Pinto et al., 2020) | 5 |
|  | (Pniak et al., 2006) | 6 |
|  | (Portela et al., 2021) | 5 |
|  | (Prakash et al., 2023) | 5 |
|  | (Pritchett & Zacharek, 2015) | 6 |
|  | (Priya et al., 2024) | 2 |
|  | (Lin et al., 2022) | 2 |
|  | (Qureshi et al., 2021) | 5 |
|  | (Raj et al., 2012) | 5 |
|  | (Ramesh & Raju, 2015) | 2 |
|  | (Razak et al., 2018) | 2 |
|  | (Rayasam et al., 2023) | 5 |
|  | (Razack et al., 1997) | 2 |
|  | (Reche et al., 2011) | 5 |
|  | (Richmon et al., 2009) | 6 |
|  | (Roberti & Goffart, 2015) | 5 |
|  | (Roland et al., 2023) | 2 |
|  | (Rosenberg et al., 1984) | 2 |
|  | (Roland et al., 2023; Rosignoli et al., 1992) | 6 |
|  | (Sadko et al., 2022) | 2 |
|  | (Sanganabhatla, 2023) | 5 |
|  | (Savage & Niewisch, 1993) | 6 |
|  | (Saxon et al., 1982) | 2 |
|  | (Scally et al., 2010) | 5 |
|  | (Schröder et al., 2015) | 6 |
|  | (Seitz et al., 2021) | 5 |
|  | (Sepúlveda et al., 2011) | 2 |
|  | (Shahabuddin et al., 2023) | 5 |
|  | (Shimizu & Yagi, 2018) | 2 |
|  | (Ésik et al., 2003) | 2 |
|  | (B. Silva et al., 2023) | 5 |
|  | (Y. H. Silva et al., 2024) | 5 |
|  | (Singh et al., 2004) | 2 |
|  | (Siriwardana et al., 2019) | 2 |
|  | (Soares Dos Reis et al., 2019) | 5 |
|  | (Soga et al., 2017) | 6 |
|  | (Spitze et al., 2014) | 5 |
|  | (Stafford et al., 1980) | 2 |
|  | (Standefers Jr & Mattox, 1986) | 6 |
|  | (Stupak et al., 2003) | 2 |
|  | (Sulman et al., 2021) | 5 |
|  | (Suzuki et al., 2017) | 5 |
|  | (Szymczyk et al., 1992) | 6 |
|  | (Takahashi et al., 1982) | 2 |
|  | (Takayama et al., 2017) | 2 |
|  | (Talebzadeh & Talebzadeh, 2023) | 2 |
|  | (Taveirne & Lucker, 2007) | 6 |
|  | (Thurkintavide et al., 2020) | 5 |
|  | (Uh et al., 2023) | 6 |
|  | (Uslu et al., 2012) | 5 |
|  | (Verriello et al., 2023) | 5 |
|  | (Vithana & Rajakaruna, 2023) | 2 |
|  | (Vlastarakos et al., 2024) | 3 |
|  | (Wang et al., 2016) | 5 |
|  | (Whizar-Lugo et al., 2006) | 2 |
|  | (Williams et al., 2013) | 5 |
|  | (Yamagiwa et al., 1981) | 2 |
|  | (Yamauchi et al., 1994) | 6 |
|  | (Yang et al., 2009) | 2 |
|  | (Yeh et al., 2009) | 2 |
|  | (Yen et al., 1991) | 6 |
|  | (Yu et al., 2013) | 6 |
|  | (Zhao et al., 2015) | 5 |
|  | (Meral & Yanardag, 2014) | 5 |
|  | (N/A, 2013) | 5 |
|  | (Adham et al., 2012) | 2 |
|  | (Connell & Park, 2018) | 2 |
|  | (Dash & Kimmelman, 1988) | 2 |
|  | (De Stefani et al., 2022) | 2 |
|  | (Moscatello et al., 1991) | 2 |
|  | (Pai et al., 2018) | 2 |
|  | (Searls et al., 2012) | 2 |
|  | (Abdeldaoui et al., 2013) | 2 |
|  | (Deganello et al., 2011) | 2 |
|  | (Portelinha et al., 2015) | 2 |
|  | (Sangeetha Priya et al., 2020) | 2 |
|  | (Stavridopoulos et al., 2023) | 2 |
|  | (Yaghoobi et al., 2010) | 5 |
|  | (Yoskovitch et al., 1998) | 2 |
|  | (Krause et al., 2001) | 2 |
|  | (Krisztián et al., 2018) | 2 |
|  | (Spalatin et al., 1973) | 2 |

***Reasons for exclusion:**

1. Studies not involving human participants;

2. Clinical signs observed other than head and neck region or not specific of the disease;

3. Studies that did not involve physical examination or physical examination performed with auxiliary equipment;

4. Studies conducted outside clinical or medical examination settings, such as community health surveys;

5. Books, conference abstracts, opinion articles, technique articles, posters, guidelines, and reviews that did not specifically address clinical signs in the head and neck region as a primary focus;

6. Full-text access unavailable, despite efforts to contact the corresponding authors.

References

Abdeldaoui, A., Oker, N., Duet, M., Cunin, G., & Tran Ba Huy, P. (2013). First Bite Syndrome: a little known complication of upper cervical surgery. *Eur Ann Otorhinolaryngol Head Neck Dis*, *130*(3), 123–129. https://doi.org/10.1016/j.anorl.2012.08.001

Abdolrahimzadeh, S., Piraino, D. C., Plateroti, R., Scuderi, G., & Recupero, S. M. (2016). Ocular Alterations in a Rare Case of Segmental Neurofibromatosis Type 1 with a Non-Classified Mutational Variant of the NF-1 Gene. *Ophthalmic Genetics*, *37*(2), 214–216. https://doi.org/10.3109/13816810.2015.1020560

Abudinen-Vasquez, S., & Marin, M. N. (2020). Management of pediatric head and neck infections in the emergency department. *Pediatr Emerg Med Pract*, *17*(11), 1–24.

Adham, M., Kurniawan, A. N., Muhtadi, A. I., Roezin, A., Hermani, B., Gondhowiardjo, S., Bing Tan, I., & Middeldorp, J. M. (2012). Nasopharyngeal carcinoma in indonesia: Epidemiology, incidence, signs, and symptoms at presentation. *Chinese Journal of Cancer*, *31*(4), 185–196. https://doi.org/10.5732/cjc.011.10328

Agarwal, S., Gupta, S., Ojha, A., & Sinha, R. (2013). Childhood vitiligo: Clinicoepidemiologic profile of 268 children from the Kumaun region of Uttarakhand, India. *Pediatric Dermatology*, *30*(3), 348–353. https://doi.org/10.1111/pde.12032

Ahmed, M. E., Abdelfattah, H. M., El-Hamd, M. A., & Amr, W. H. (2018). Leser-trélat syndrome associated with cancer larynx: A case report. *Egyptian Journal of Ear, Nose, Throat and Allied Sciences*, *19*(1), 21–23. https://doi.org/10.21608/ejentas.2018.10295

Ahmetgjekaj, I., Rahman, M., Hyseni, F., Guy, A., Madani, K., Saliaj, K., Guy, A., Vokshi, V., Kola, I., & Musa, J. (2021). A case report of Joubert syndrome with renal involvement and seizures in a neonate. *Radiology Case Reports*, *16*(5), 1075–1079. https://doi.org/10.1016/j.radcr.2021.02.031

Aktan, S. (2012). Cervical and intracranial artery dissections. *Turk Beyin Damar Hastaliklar Dergisi*, *18*(2), 25–30. https://doi.org/10.5505/tbdhd.2012.65375

Alfonso, L. S., & Centelles, I. A. (2016). Treacher Collins syndrome in a cuban family. Case presentation. *Revista Habanera de Ciencias Medicas*, *15*(3), 408–417. https://www.scopus.com/inward/record.uri?eid=2-s2.0-85025699361&partnerID=40&md5=5941e1422f71537328a4185f742724f5

Alkali, A., King, C., & Leonard, N. (2009). Bowel-associated dermatosis-arthritis syndrome: A case report. *Journal of the American Academy of Dermatology*, *60*(3), AB59. https://doi.org/10.1016/j.jaad.2008.11.272

Allegrini, D., Autelitano, A., Nocerino, E., Fogagnolo, P., De Cillà, S., & Rossetti, L. (2016). Grisel’s syndrome, a rare cause of anomalous head posture in children: A case report. *BMC Ophthalmology*, *16*(1). https://doi.org/10.1186/s12886-016-0197-1

AlOtaibi, N. M., Alanzan, A. N., Aloshaywi, A. K., Alotaibi, N. H., Alanazi, A. F., Alrajhi, F. M., & Meaadi, J. (2025). First Bite Syndrome: A Rare Post-Surgical Complication Following a Carotid Body Tumor Excision. *Cureus*. https://doi.org/10.7759/cureus.76827

Arcangeli, F., & Brunelli, D. (2015). Vascular birthmarks: Vascular malformations and haemangiomas. In *European Handbook of Dermatological Treatments, Third Edition* (pp. 1009–1021). https://doi.org/10.1007/978-3-662-45139-7_103

Arshad, W., Mahmood Kamal, M., Rafique, Z., Rahat, M., & Mumtaz, H. (2022). Case of maxillary actinomycotic osteomyelitis, a rare post COVID complication-case report. *Annals of Medicine and Surgery*, *80*. https://doi.org/10.1016/j.amsu.2022.104242

Ashimori, N., Hayashi, Y., & Hoshino, T. (2003). Two Cases of Amyloidosis of the External Auditory Canals. *Practica Oto-Rhino-Laryngologica*, *96*(12), 1049–1054. https://doi.org/10.5631/jibirin.96.1049

Askari, E., Zhao, N., Polizu, C., & Treidler, S. (2024). Lyme Disease with Isolated Sixth Cranial Nerve Palsy. *Neurology*, *102*(17). https://doi.org/10.1212/WNL.0000000000206030

Asmus, F., von Coelln, R., Boertlein, A., Gasser, T., & Mueller, J. (2009). Reverse sensory geste in cervical dystonia. *Mov Disord*, *24*(2), 297–300. https://doi.org/10.1002/mds.22406

Baiocco, F. A., Gamoletti, R., Negri, A., & Rognoni, S. (1984). Blue rubber bleb nevus syndrome: A case with predominantly ENT localization. *Journal of Laryngology and Otology*, *98*(3), 317–319. https://doi.org/10.1017/S0022215100146638

Bakhous, A., Ghaffar, U., Singh, R., Elnagar, E., & Bhatti, R. (2015). Renal tubular neutrophilic casts in patient with sweet syndrome. *American Journal of Kidney Diseases*, *65*(4), A21. https://www.embase.com/search/results?subaction=viewrecord&id=L71875018&from=export

Bakhshaee, M., Sarvghad, M. R., Khazaeni, K., Movahed, R., & Hoseinpour, A. M. (2014). HIV: An Epidemiologic study on Head and Neck Involvement in 50 Patients. In *Iranian Journal of Otorhinolaryngology* (Vol. 26, Issue 2).

Bandla, M., Boyapati, A., & Lee, I. (2022). A Case of Eruptive Cherry Angiomas Associated with Immunoglobulin type gamma 4-related disease (IgG4-RD). *Australasian Journal of Dermatology*, *63*, 62. https://doi.org/10.1111/ajd.13_13832

Bardan, A., & Hata, T. (2007). Dermatologic signs of systemic disease. In *Head and Neck Manifestations of Systemic Disease* (pp. 543–564). https://www.scopus.com/inward/record.uri?eid=2-s2.0-85057664914&partnerID=40&md5=369b4ea210cbc4d5deabb8eac94eeeac

Barrett, A. W. (2012). Wegener’s granulomatosis of the major salivary glands. *J Oral Pathol Med*, *41*(10), 721–727. https://doi.org/10.1111/j.1600-0714.2012.01141.x

Berisavac, I., Radojicic, A., Savic, O., Jovanovic, D., Bogosavljevic, V., & Beslac-Bumbasirevic, L. (2009). Clinical presentation of headaches in patients with cervicocerebral arterial dissections. *European Journal of Neurology*, *16*(3).

Berth-Jones, J. (1996). Six area, six sign atopic dermatitis (SASSAD) severity score: a simple system for monitoring disease activity in atopic dermatitis. *Br J Dermatol*, *135*, 25–30. https://doi.org/10.1111/j.1365-2133.1996.tb00706.x

Bhidayasiri, R., & Tarsy, D. (2012). Anterocollis in Parkinsonism. In *Curr. Clin. Neurol.* (pp. 134–135). https://doi.org/10.1007/978-1-60327-426-5_63

Binenbaum, G., Levin, A. V, & Rubin, S. E. (2014). Retinal Hemorrhages are present: How certain can you be it is AHT? *Journal of AAPOS*, *18*(4), e39–e40. https://doi.org/10.1016/j.jaapos.2014.07.128

Bonometti, A., Passoni, E., & Berti, E. (2021). Benign cephalic histiocytosis: A forgotten self-healing condition of childhood. *Virchows Archiv*, *479*, S200–S201. https://doi.org/10.1007/s00428-021-03157-8

Borrue-Fernandez, C., & Gomez-Raposo, C. (2011). Harlequin syndrome as first manifestation of tumoral spinal cord compression. *European Journal of Neurology*, *18*, 605. https://doi.org/10.1111/j.1468-1331.2011.03552.x

Boukovalas, S., Mays, A. C., & Selber, J. C. (2019). Botulinum Toxin Injection for Lower Face and Oral Cavity Raynaud Phenomenon After Mandibulectomy, Free Fibula Reconstruction, and Radiation Therapy. *Ann Plast Surg*, *82*(1), 53–54. https://doi.org/10.1097/sap.0000000000001622

Bumsted, R. M. (1980). Thyroid disease: a guide for the head and neck surgeon. *Ann Otol Rhinol Laryngol Suppl*, *89*(4), 1–16. https://doi.org/10.1177/00034894800894s301

Calderon-Castrat, X., Canueto, J., Alonso-San Pablo, M. T., Roman-Curto, C., & Fernandez-Lopez, E. (2017). Intraoral involvement in sebaceous nevus syndrome. *Pediatric Dermatology*, *34*, S21. https://doi.org/10.1111/pde.13268

Callen, J. P. (1985). Systemic lupus erythematosus in patients with chronic cutaneous (discoid) lupus erythematosus. Clinical and laboratory findings in seventeen patients. *J Am Acad Dermatol*, *12*(2), 278–288. https://doi.org/10.1016/s0190-9622(85)80036-0

Calzadilla, A. S., & Zuleta, J. (2015). More than skin deep. *Journal of General Internal Medicine*, *30*, S427–S428. https://www.embase.com/search/results?subaction=viewrecord&id=L71878336&from=export

Capodiferro, S., Limongelli, L., & Favia, G. (2021). Oral and Maxillo-Facial Manifestations of Systemic Diseases: An Overview. *Medicina (Kaunas)*, *57*(3). https://doi.org/10.3390/medicina57030271

Çello, O., Dogan, Y. P., Okur, S. Ç., Burnaz, O., & Çaglar, N. S. (2013). A neurofibromatosis case with myopathy. *Turkiye Fiziksel Tip ve Rehabilitasyon Dergisi*, *59*, 395. https://doi.org/10.4274/tftr.24.59.1

Chen, H. C., Jen, Y. M., Wang, C. H., Lee, J. C., & Lin, Y. S. (2007). Etiology of vocal cord paralysis. *ORL J Otorhinolaryngol Relat Spec*, *69*(3), 167–171. https://doi.org/10.1159/000099226

Chen, T. H., Hsu, P. S., Chang, K. L., & Lin, H. S. (2023). Delayed Unilateral Eagle Syndrome with Fractured Styloid Process. *Acta Neurologica Taiwanica*, *32*(1), 25–28. https://www.embase.com/search/results?subaction=viewrecord&id=L2023318018&from=export

Chong, V. (2015). Orbital tumours. *Cancer Imaging*, *15*. https://www.embase.com/search/results?subaction=viewrecord&id=L615293398&from=export

Christen, H. J., Hanefeld, F., Eiffert, H., & Thommessen, R. (1993). Epidemiology and clinical manifestations of lyme borreliosis in childhood - a prospective multicenter study with special regard to neuroborreliosis. *ACTA PAEDIATRICA*, *82*, 1–76.

Chung, S. R., Kim, G. J., Choi, Y. J., Cho, K. J., Suh, C. H., Kim, S. C., Baek, J. H., Lee, J. H., Yang, M. K., & Sa, H. S. (2022). Clinical and Radiological Features of Diffuse Lacrimal Gland Enlargement: Comparisons among Various Etiologies in 91 Biopsy-Confirmed Patients. *Korean Journal of Radiology*, *23*(10), 976–985. https://doi.org/10.3348/kjr.2022.0233

Coleman, I., Ruiz, G., Brahmbhatt, S., & Ackerman, L. (2020). Acute generalized exanthematous pustulosis and Stevens-Johnson syndrome overlap due to hydroxychloroquine: a case report. *Journal of Medical Case Reports*, *14*, 1–4. https://doi.org/https://doi.org/10.1186/s13256-020-02504-8

Connell, J. T., & Park, J. H. (2018). Acute peritonsillar swelling: a unique presentation for Kawasaki disease in adolescence. *BMJ Case Rep*, *2018*. https://doi.org/10.1136/bcr-2018-224441

Contrucci, R. B., & Martin, D. B. (2015). Sweet syndrome: A case report and review of the literature. *Ear Nose Throat J*, *94*(7), 282–284. https://doi.org/10.1177/014556131509400712

Cooper, R. H., Smalligan, R. D., Stoughton, C., Richardson, J. M., Duke, D., Geissler, A. L., Rao, A. K., Bennett, S. D., Harvey, R., Syeda, S., & Nixon-Lewis, B. (2014). When eyes droop and things get blurry - Think botulism. *Journal of General Internal Medicine*, *29*, S468. https://www.embase.com/search/results?subaction=viewrecord&id=L71495865&from=export

Cutright, D. E., Carter, H. G., Daniels, J. L., & Huget, E. F. (1977). Clinical examination of the head and neck. *J Am Dent Assoc*, *94*(5), 915–917. https://doi.org/10.14219/jada.archive.1977.0062

Dash, G. I., & Kimmelman, C. P. (1988). Head and neck manifestations of sarcoidosis. *Laryngoscope*, *98*(1), 50–53. https://doi.org/10.1288/00005537-198801000-00011

De Stefani, A., Dassie, F., Wennberg, A., Preo, G., Muneratto, A., Fabris, R., Maffei, P., Gracco, A., & Bruno, G. (2022). Oral Manifestations and Maxillo-Facial Features in the Acromegalic Patient: A Literature Review. *J Clin Med*, *11*(4). https://doi.org/10.3390/jcm11041092

Dede, I., Karasimav, O., GÃ¶ktepe, A. S., & Tan, A. K. (2011). Klippel-feil syndrome: Case report. *Turkiye Fiziksel Tip ve Rehabilitasyon Dergisi*, *57*, 333. https://www.embase.com/search/results?subaction=viewrecord&id=L71016921&from=export

Deganello, A., Meccariello, G., Busoni, M., Franchi, A., & Gallo, O. (2011). First bite syndrome as presenting symptom of parapharyngeal adenoid cystic carcinoma. *J Laryngol Otol*, *125*(4), 428–431. https://doi.org/10.1017/s002221511000294x

Delgado Martínez, J. R., Inglada Galiana, L., Sánchez Hernández, J. A., Cabrera Galván, J. J., Hernández Hernández, B., & Amerigo García, M. J. (1989). Sweet syndrome: presentation of an atypical case and review of the literature. *Revista clÃnica espaÃ±ola*, *185*(5), 246–249. https://www.embase.com/search/results?subaction=viewrecord&id=L20744330&from=export

Dickstein, L., Anand, P., Mathur, R., Haight, T., & Zhang, J. (2018). Reversible cerebral vasoconstriction syndrome presenting as third cranial nerve palsy. *Neurology*, *90*(15). https://www.embase.com/search/results?subaction=viewrecord&id=L622308610&from=export

Dogra, S., Parsad, D., Handa, S., & Kanwar, A. J. (2005). Late onset vitiligo: a study of 182 patients. *Int J Dermatol*, *44*(3), 193–196. https://doi.org/10.1111/j.1365-4632.2004.01948.x

Dorsch, J. N. (2014). Neurologic syndromes of the head and neck. *Prim Care*, *41*(1), 133–149. https://doi.org/10.1016/j.pop.2013.10.012

Dunphy, L., Devine, J., & McMahon, J. (2011). A retrospective review of parotid gland surgery carried out in a Regional Maxillofacial Unit. *British Journal of Oral and Maxillofacial Surgery*, *49*, S52. https://doi.org/10.1016/j.bjoms.2011.03.082

Elshafei, M. (2012). Superior vena cava syndrome - Changing etiology in the Third Millennium “‘downhill’” esophageal varices bleeding due to superior vena cava syndrome in Behcet’s disease. *International Journal of Rheumatic Diseases*, *15*, 156. https://doi.org/10.1111/j.1756-185X.2012.01809.x

Eroz, R., Dogan, M., Bolu, S., & Yuce, H. (2017). A seven years old girl with klippel-feil syndrome, bilateral sprengel deformity, congenital unilateral renal agenesis and a heterozygous mutation M680I(G>C) in the MEFV gene. *Konuralp Tip Dergisi*, *9*(2), 90–93. https://doi.org/10.18521/ktd.300827

Ésik, O., Csere, T., Stefanits, K., Lengyel, Z., Sáfrány, G., Vönöczky, K., Lengyel, E., Nemeskéri, C., Repa, I., & Trón, L. (2003). A review on radiogenic Lhermitte’s sign. *PATHOLOGY & ONCOLOGY RESEARCH*, *9*(2), 115–120.

Fieux, M., Franco-Vidal, V., Devic, P., Bricaire, F., Charpiot, A., Darrouzet, V., Denoix, L., Gatignol, P., Guevara, N., Montava, M., Roch, J. A., TankÃ©rÃ©, F., Tronche, S., Veillon, F., Vergez, S., Vincent, C., Lamas, G., & Tringali, S. (2020). French Society of ENT (SFORL) guidelines. Management of acute Bell’s palsy. *European Annals of Otorhinolaryngology, Head and Neck Diseases*, *137*(6), 483–488. https://doi.org/10.1016/j.anorl.2020.06.004

Fogo, A., Hunter, H., McLornan, D., & Walsh, S. (2013). A widespread granulomatous eruption heralding the onset of myelodysplastic syndrome. *British Journal of Dermatology*, *169*, 98. https://doi.org/10.1111/bjd.12369

Forbes, B. J., Levin, A. V, & Rubin, S. E. (2009). Retinal hemorrhages are present-so what does it mean? *Journal of AAPOS*, *13*(1), e33–e34. https://doi.org/10.1016/j.jaapos.2008.12.153

GÃ¼leÃ§, F., Alper, Y., Ã‡elebisoy, N., & AkyÃ¼rekli, Ã. (2007). Isolated hypoglossal nerve palsy due to internal carotid artery dissection. *Journal of Neurological Sciences*, *24*(2), 178–181. https://www.embase.com/search/results?subaction=viewrecord&id=L47099153&from=export

Gadalla, W., & Kulkarni, R. (2021). Toxic epidermal necrolysis and hydroxychloroquine. *Intensive Care Medicine Experimental*, *9*. https://doi.org/10.1186/s40635-021-00415-6

Gandia, D., Wibault, P., Guillot, T., Bensmaine, A., Armand, J. P., Marandas, P., Luboinski, B., & Cvitkovic, E. (1993). Simultaneous chemoradiotherapy as salvage treatment in locoregional recurrences of squamous head and neck cancer. *Head Neck*, *15*(1), 8–15. https://doi.org/10.1002/hed.2880150103

Gilio, M., Tramontano, G., Cutro, M. S., Carbone, T., Picerno, V., Bottoni, U., Padula, A., & D’Angelo, S. (2018). The use of belimumab in recalcitrant cutaneous lupus: A case report. *Lupus Science and Medicine*, *5*, A103. https://doi.org/10.1136/lupus-2018-abstract.180

Gomes, A. C. A., Pita Neto, I. C., Melo, D. G., & Dias, E. (2007). Osteorradionecrose resultando em uma fratura patológica de mandíbula: relato de caso clínico. *Revista Odonto Ciência*, *22*(57).

Gordon, K. D., & Wain, E. M. (2009). Sorafenib-induced erythema multiforme. *British Journal of Dermatology*, *161*, 49. https://doi.org/10.1111/j.1365-2133.2009.09126.x

Gouda, G., Pyne, J., & Dicker, T. (2022). Pigmented Macules on the Head and Neck: A Systematic Review of Dermoscopy Features. *Dermatol Pract Concept*, *12*(4), e2022194. https://doi.org/10.5826/dpc.1204a194

Habib, A., & Raza, N. (2012). Clinical pattern of vitiligo. *J Coll Physicians Surg Pak*, *22*(1), 61–62.

Haebich, G., Pilz, D., & Li, V. (2019). A rare case of mutation-positive cardiofaciocutaneous syndrome with associated multiple epidermoid cysts. *British Journal of Dermatology*, *181*, 82. https://doi.org/10.1111/bjd.17890

Hafi, N. A. B., Bachaspatimayum, R., Soraisham, R., & Muhammed, N. C. P. (2019). Halo Nevi in children: A separate entity or a sign of vitiligo. *INDIAN JOURNAL OF PAEDIATRIC DERMATOLOGY*, *20*(3), 227–230. https://doi.org/10.4103/ijpd.IJPD_88_18

Handa, S., & Dogra, S. (2003). Epidemiology of childhood vitiligo: a study of 625 patients from north India. *Pediatr Dermatol*, *20*(3), 207–210. https://doi.org/10.1046/j.1525-1470.2003.20304.x

Hannah-Shmouni, F., & Sirrs, S. M. (2015). Unlocking pandora’s box: Late-onset hypoparathyroidism and mitochondrial disease. *Endocrine Reviews*, *36*. https://www.embase.com/search/results?subaction=viewrecord&id=L613817694&from=export

Harris, J. P., & Weisman, M. H. (2007). Head and neck manifestations of systemic disease. In *Head and Neck Manifestations of Systemic Disease*. https://www.scopus.com/inward/record.uri?eid=2-s2.0-85057676156&partnerID=40&md5=38c549f1701595b512b9c75b24656562

Hassan, D., Walker, A., & Kim, D. (2017). Asymmetrical tonsils in a 51-year-old ex-smoker: An unexpected diagnosis. *Journal of Laryngology and Otology*, *131*(9), 7. https://doi.org/10.1017/S0022215117001578

Hilal, E. Y. (1994). Diagnosis of head and neck cancer. *J Med Liban*, *42*(4), 212–215.

Htwe, S. H. (2020). A case of adult-onset Kawasaki disease. *British Journal of Dermatology*, *182*(4), e126. https://www.embase.com/search/results?subaction=viewrecord&id=L633049679&from=export

Iijima, H., Sakai, A., Ebisumoto, K., Yamauchi, M., Maki, D., Teramura, T., Saito, K., Yamazaki, A., Inagi, T., Yamamoto, A., Ashida, H., Sato, Y., Sato, S., & Okami, K. (2024). A retrospective analysis of syphilis cases with a focus on otolaryngology at a university hospital. *Auris Nasus Larynx*, *51*(6), 1016–1024. https://doi.org/10.1016/j.anl.2024.09.009

Ipek, M. S., Okur, N., & Akdeniz, O. (2015). Klippel-Feil syndrome and interruption of aortic arch. *Child’s Nervous System*, *31*(10), 1976. https://doi.org/10.1007/s00381-015-2811-6

Isaacson, B., Mirabal, C., Kutz Jr, J. W., Lee, K. H., & Roland, P. S. (2010). Pediatric otogenic intracranial abscesses. *Otolaryngology - Head and Neck Surgery*, *142*(3), 434–437. https://doi.org/10.1016/j.otohns.2009.11.030

Jacobs, J. R., Waters, R. C., & Toomey, J. M. (1979). Head and neck manifestations of SLE. *Am Fam Physician*, *20*(6), 97–99.

Jiang, Y., Hua, Q., Ren, J., Zeng, F., Sheng, J., Liao, H., Zhang, Z., & Guan, H. (2017). Eosinophilic hyperplastic lymphogranuloma: Clinical diagnosis and treatment experience of 41 cases. *American Journal of Otolaryngology - Head and Neck Medicine and Surgery*, *38*(5), 626–629. https://doi.org/10.1016/j.amjoto.2017.07.007

Karam, S., & Hahr, A. (2019). Hypoparathyroidism. In *Metabolic Bone Diseases: A Case-Based Approach* (pp. 55–66). https://doi.org/10.1007/978-3-030-03694-2

Kasem Ali Sliman, R., van Montfrans, J. M., Nassrallah, N., & Hamad Saied, M. (2023). Retropharyngeal abscess-like as an atypical presentation of Kawasaki disease: a case report and literature review. In *Pediatric Rheumatology* (Vol. 21, Issue 1). BioMed Central Ltd. https://doi.org/10.1186/s12969-023-00812-z

Kaushik, V., Malik, T. H., Bishop, P. W., & Jones, P. H. (2004). Histiocytic necrotising lymphadenitis (Kikuchi’s disease): A rare cause of cervical lmyphadenopathy. *Surgeon*, *2*(3), 179–182. https://doi.org/10.1016/S1479-666X(04)80084-2

Khatri, K., Khatri, V., Bichile, T., & Friedman, H. (2012). Horner syndrome-not to be sneezed at! *Journal of General Internal Medicine*, *27*, S442. https://www.embase.com/search/results?subaction=viewrecord&id=L71297278&from=export

Kim, M. K., & Alvi, A. (1999). Common head and neck manifestations of AIDS. *AIDS Patient Care STDS*, *13*(11), 641–644. https://doi.org/10.1089/apc.1999.13.641

Kim, Y. S., & Lee, C. W. (2009). Clinical features of the skin lesions of patients with chronic cutaneous lupus erythematosus and examination of the factors that are relevant to its transformation to systemic lupus erythematosus. *Korean Journal of Dermatology*, *47*(3), 268–277. https://www.scopus.com/inward/record.uri?eid=2-s2.0-68949220870&partnerID=40&md5=43a5153f3f3beb98e6200865581805b0

Ko, H. C., Powers, A. R., Sheu, R. D., Kerns, S. L., Rosenstein, B. S., Krieger, S. C., Mourad, W. F., Hu, K. S., Gupta, V., & Bakst, R. L. (2015). Lhermitte’s Sign following VMAT-Based Head and Neck Radiation-Insights into Mechanism. *PLOS ONE*, *10*(10). https://doi.org/10.1371/journal.pone.0139448

Koch, U., Pau, H. W., & Klingmuller, G. (1978). Lepromatous leprosy in the head and neck region. *Therapiewoche*, *28*(20), 4122–4127. https://www.embase.com/search/results?subaction=viewrecord&id=L8349653&from=export

Kozicky, O., Yamada, Y., & Harrington, M. (2018). Know thy selfie, know thy diagnosis: A picture perfect presentation of horner syndrome. *Journal of General Internal Medicine*, *33*(2), 553. https://www.embase.com/search/results?subaction=viewrecord&id=L622330393&from=export

Krause, M. H., Ulrich, J., & Gollnick, H. (2001). [Systematic sebaceous nevus with multiple secondary tumors as the chief symptom of Schimmelpenning-Feuerstein-Mims syndrome]. *Hautarzt*, *52*(4), 339–343. https://doi.org/10.1007/s001050051319

Krisztián, G., Ifeoluwa, A., István, L., Tünde, B., & Tamás, K. (2018). Carotid body tumor and its treatment *A case report*. *ORVOSI HETILAP*, *159*(36), 1487–1492. https://doi.org/10.1556/650.2018.31078

Kumar, V., Iqbal, A., Nawaz, A., Kumar, R., Sandesh, R., Kanukuntla, A. K., Kata, P., Veeraballi, S., & Shaikh, S. (2021). The need for vigilance: A rare association of pancreatic atrophy with hermansky-pudlak syndrome. *American Journal of Gastroenterology*, *116*, S727. https://doi.org/10.14309/01.ajg.0000780004.90727.07

Kurtz, K. J., Walker, H. K., Hall, W. D., & Hurst, J. W. (1990). Bruits and Hums of the Head and Neck. In *Clinical Methods: The History, Physical, and Laboratory Examinations*. Butterworths    Copyright © 1990, Butterworth Publishers, a division of Reed Publishing.

Lai, K., Lee, K., & Chung, C. (2023). Fatal Pulmonary Arteriovenous Malformation Rupture. *American Journal of Respiratory and Critical Care Medicine*, *207*(1). https://doi.org/10.1164/ajrccm-conference.2023.A57

Lee, S. (2018). Harlequin syndrome in a subject with multinodular goiter and an elevated tryptase levels. *Journal of the American Academy of Dermatology*, *79*(3), AB150. https://doi.org/10.1016/j.jaad.2018.05.619

Leung, A. K. C., Lam, J. M., Leong, K. F., & Hon, K. L. (2021). Vitiligo: An Updated Narrative Review. *CURRENT PEDIATRIC REVIEWS*, *17*(2), 76–91. https://doi.org/10.2174/1573396316666201210125858

Leung, W. M., Tsang, N. M., Chang, F. T., & Lo, C. J. (2005). Lhermitte’s sign among nasopharyngeal cancer patients after radiotherapy. *Head Neck*, *27*(3), 187–194. https://doi.org/10.1002/hed.20140

Lin, Q., Zhang, B., Zheng, W., Li, M., Zhao, Y., Zeng, X., Zhang, F., Wang, L., & Li, L. (2022). Clusters of clinical and immunologic features in patients with bullous systemic lupus erythematosus: experience from a single-center cohort study in China. *Orphanet Journal of Rare Diseases*, *17*, 1–10. https://doi.org/https://doi.org/10.1186/s13023-022-02445-z

Lindsay, F. W., Mullin, D., & Keefe, M. A. (2003). Subacute hypoglossal nerve paresis with internal carotid artery dissection. *Laryngoscope*, *113*(9), 1530–1533. https://doi.org/10.1097/00005537-200309000-00022

LITTLE, S. C. (1946). ELECTRICAL PARESTHESIAS IN THE EXTREMITIES FOLLOWING INJURY TO THE CENTRAL NERVOUS SYSTEM. *ARCHIVES OF NEUROLOGY AND PSYCHIATRY*, *56*(4), 417–427.

Liu, R., Fang, Y., Li, Z., & Yang, F. (2024). Advancements in ophthalmological research on Waardenburg syndrome. *Chinese Journal of Ocular Fundus Diseases*, *40*(7), 560–563. https://doi.org/10.3760/cma.j.cn511434-20231107-00445

Louka, M., Ntasi, E., Petropoulou, T., & Valari, M. (2023). An infant with PHACE syndrome and lower gastrointestinal bleeding. *Pediatric Dermatology*, *40*, 82–83. https://doi.org/10.1111/pde.15301

Maceri, D. R. (1986). Head and neck manifestations of endocrine disease. *Otolaryngol Clin North Am*, *19*(1), 171–180.

Mahtta, D., Ross, S., Goede, D., & Bavry, A. (2018). Transient post-prandial amaurosis fugax as a rare presentation of carotid steal syndrome. *Journal of the American College of Cardiology*, *71*(11). https://doi.org/10.1016/S0735-1097(18)32720-7

Malavika, V., Kavitha, L., & Ranganathan, K. (2023). Autoimmune Diseases Affecting the Orofacial Region - An Overview. *ORAL & MAXILLOFACIAL PATHOLOGY JOURNAL*, *14*(1), 85–95.

Maniglia, J. V., Padovani Júnior, J. A., Medeiros, A. P., & Fernandes, A. M. (1996). Proptose: diagnóstico diferencial. *Folha méd*, *113*, 11–15. https://pesquisa.bvsalud.org/portal/resource/pt/lil-176624

Martin, E. G., Kathleen, T., Grewal, S., John, K. N., Mehta, A., & Durrani, Z. U. (2020). Why is he still dizzy? *Journal of General Internal Medicine*, *35*, S623. https://doi.org/10.1007/s11606-020-05890-3

Martinez, V., Ramos, A., Maldonado, C., Gomez, E., & Jurado, F. (2014). Monozygotic twins concordant for disseminated discoid lupus. *Journal of the American Academy of Dermatology*, *70*(5), AB59. https://doi.org/10.1016/j.jaad.2014.01.244

Masele, A., Sohal, K. S., Kalyanyama, B. M., Owibingire, S. S., & Simon, E. N. M. (2020). Head and neck lesions among HIV/AIDS patients on highly active antiretroviral therapy attending the Muhimbili National Hospital in Dar es Salaam, Tanzania. *Frontiers of Oral and Maxillofacial Medicine*, *2*. https://doi.org/10.21037/fomm.2019.12.01

Matsuo, K., Makino, M., Kuriyama, N., Ueda, Y., & Nakajima, K. (1998). A case of juvenile muscular atrophy of the upper limb with intraspinal cavity formation. *Clinical Neurology*, *38*(7), 649–652. https://www.embase.com/search/results?subaction=viewrecord&id=L28551417&from=export

McCusker, S., Hamilton, M., & Beattie, P. (2024). The phenotypic spectrum of MBTPS2 mutations: two illustrative cases. *British Journal of Dermatology*, *192*, i184–i185. https://doi.org/10.1093/bjd/ljae090.390

McNeilly, B. P., & Wilkerson, R. G. (2022). Clinical Communications: Adult. *JOURNAL OF EMERGENCY MEDICINE*, *63*(1), E31–E33. https://doi.org/10.1016/j.jemermed.2022.01.031

Mehta, R. V, Cobb, J., & Mehta, R. (2018). Acquired angioedema: A unique complication in a patient with lupus nephritis. *Journal of the American Society of Nephrology*, *29*, 1126. https://www.embase.com/search/results?subaction=viewrecord&id=L633733076&from=export

Mengen, E., Kotan, L. D., Topaloglu, A. K., & Yuksel, B. (2017). A novel frameshift mutation in ESCO2 gene cause roberts syndrome: Case presentation. *Hormone Research in Paediatrics*, *88*, 381. https://doi.org/10.1159/000481424

Meral, A. O., & Yanardag, A. G. (2014). A case of petechiael rash on the face. *JDDG - Journal of the German Society of Dermatology*, *12*, 48. https://doi.org/10.1111/j.1610-0387.2014.12387

Moazzez, A. H., & Alvi, A. (1998). Head and neck manifestations of AIDS in adults. *Am Fam Physician*, *57*(8), 1813–1822.

Mohammed, A. A., & Al-Gadi, M. A. (2003). Neonatal Staphylococcal scalded skin syndrome complicating ileal atresia. *Saudi Med J*, *24*(5), 538–541.

Mohindra, A., Venkatasami, M., & Green, J. (2015). First bite syndrome secondary to open TMJ surgery. *International Journal of Oral and Maxillofacial Surgery*, *44*, e264. https://doi.org/10.1016/j.ijom.2015.08.245

Mon, Y., & Nakamura, N. (2000). Velopharyngeal palsy : A case report. *Clinical Neurology*, *40*(4), 364–366. https://www.embase.com/search/results?subaction=viewrecord&id=L30693808&from=export

Moroco, A. E., & McGinn, J. D. (2018). Head and Neck Manifestations of Systemic Disease. *Medical Clinics of North America*, *102*(6), 1095–1107. https://doi.org/10.1016/j.mcna.2018.06.009

Moscatello, A. L., Worden, D. L., Nadelman, R. B., Wormser, G., & Lucente, F. (1991). Otolaryngologic aspects of Lyme disease. *Laryngoscope*, *101*(6), 592–595. https://doi.org/10.1288/00005537-199106000-00004

Mozumder, A. S., Gaub, M. B., Simon, R. C., Fisher, J. L., & Kaddouh, F. (2022). Moyamoya Syndrome in the Setting of Sturge Weber: A Case Report. *Neurology*, *98*(18). https://www.embase.com/search/results?subaction=viewrecord&id=L638417012&from=export

Muecke, M., & Amedee, R. G. (1994). Head and neck manifestations of neurofibromatosis. *The Journal of the Louisiana State Medical Society : Official Organ of the Louisiana State Medical Society*, *146*(5), 183–186. https://www.scopus.com/inward/record.uri?eid=2-s2.0-0028438648&partnerID=40&md5=c57e1ce77019816b89137e936c3c626f

Mumtaz, M. A., Jhand, A., & Gbadamosi-Akindele, M. (2018). Incidental horner’s syndrome in a patient with diabetic foot ulcer. *Journal of General Internal Medicine*, *33*(2), 542–543. https://www.embase.com/search/results?subaction=viewrecord&id=L622329592&from=export

Murakawa, G. J., Kerschmann, R., & Berger, T. (1996). Cutaneous Cryptococcus infection and AIDS. Report of 12 cases and review of the literature. *Arch Dermatol*, *132*(5), 545–548.

N/A. (2013). Singapore Malaysia Congress of Medicine 2013. *Annals of the Academy of Medicine, Singapore*, *42*, S1. https://www.embase.com/search/results?subaction=viewrecord&id=L75000538&from=export

Nair, D., Moore, S., & Bhatnagar, D. (2014). A mass of mucor. *Journal of General Internal Medicine*, *29*, S277. https://www.embase.com/search/results?subaction=viewrecord&id=L71495320&from=export

Nuta, M. V, & Puianu, M. (2019). Claude Bernard Horner syndrome aft er a thyroid nodule’s ecotherapy. *Romanian Journal of Neurology*, *18*, 48–49. https://doi.org/10.37897/RJN.2019.S.2

Pai, D., Kamath, A. T., Kini, P., Bhagania, M., & Kumar, S. (2018). Concomitant Temporomandibular Joint Ankylosis and Maxillomandibular Fusion in a Child with Klippel- Feil Syndrome: A Case Report. *J Clin Pediatr Dent*, *42*(5), 386–390. https://doi.org/10.17796/1053-4625-42.5.11

Pan, D., Lee, S., Gabra, N. I., & Mathew, J. (2017). Miller fisher syndrome and neuroborreliosis: A clinical conundrum. *Chest*, *152*(4), A362. https://doi.org/10.1016/j.chest.2017.08.388

Park, S. N., Park, K. H., & Kim, D. H. (2012). Palatal myoclonus associated with orofacial buccal dystonia. *Clinical and Experimental Otorhinolaryngology*, *5*(1), 44–48. https://doi.org/10.3342/ceo.2012.5.1.44

Parris, W. C. V, Kirshner, H., & Brin, E. (1987). Herpes zoster ophthalmicus associated with contralateral hemiplegia. *Clinical Journal of Pain*, *3*(4), 219–222. https://doi.org/10.1097/00002508-198712000-00007

Pellitteri, P. K. (2007). Cervical adenitis and pain. In *Head and Neck Manifestations of Systemic Disease* (pp. 527–542). https://www.scopus.com/inward/record.uri?eid=2-s2.0-85057662908&partnerID=40&md5=e77c3d915931ebea4398c838025b7847

Picardo, S., Rodríguez Genta, S., & Rey, E. (n.d.). *FUNDAMENTOS DE ELECCIÓN TERAPÉUTICA: OSTEONECROSIS MAXILAR ASOCIADA A DROGAS ANTIRRESORTIVAS (MRONJ)*.

Pinto, C., Cardoso, L., Martins, R. C., Alves, J. E., & Ramos, C. (2020). Congenital horner syndrome associatedwith ipsilateral internal carotid artery hypoplasia. *Neuroradiology*, *62*(1), 120–121. https://doi.org/10.1007/s00234-019-02317-7

Pniak, T., Mrázková, E., Mrázek, J., Chmurová, R., & Zeleník, K. (2006). Herpes zoster oticus. *Otorinolaryngologie a Foniatrie*, *55*(4), 241–245. https://www.scopus.com/inward/record.uri?eid=2-s2.0-33846067189&partnerID=40&md5=98454faa2a4dc75ffec1607743ac4c83

Portela, M., Lorga, T., Baptista, M., Bruxelas, C., & Portelinha, J. (2021). Wyburn Mason Syndrome presenting with unilateral retinal racemose hemangioma - a case report with multimodal imaging. *Ophthalmologica*, *244*. https://www.embase.com/search/results?subaction=viewrecord&id=L637275376&from=export

Portelinha, J., Passarinho, M. P., & Costa, J. M. (2015). Neuro-ophthalmological approach to facial nerve palsy. *Saudi J Ophthalmol*, *29*(1), 39–47. https://doi.org/10.1016/j.sjopt.2014.09.009

Prakash, M., Harrison, S., Dewar, G., Kim, M., Davis, P., Bunton, R., & Parry, D. (2023). Recurrent Pneumothoraxes From Rare Birt-Hogg-Dube Syndrome. *Heart Lung and Circulation*, *32*, S51–S52. https://doi.org/10.1016/j.hlc.2023.04.144

Pritchett, C. V, & Zacharek, M. A. (2015). Raeder syndrome: Paratrigeminal oculosympathetic syndrome presenting as a manifestation of chronic sinusitis. *Ear Nose Throat J*, *94*(12), E22-5.

Priya, T. A., Sangeetha, S., & Singh, R. P. (2024). Clinicopathological Study Of Discoid Lupus Erythematosus. *Research Journal of Pharmaceutical, Biological and Chemical Sciences*, *15*(1), 1–7. https://doi.org/10.33887/rjpbcs/2024.15.1.1

Qureshi, R., Fatima, S., & Usman, M. (2021). Spectrum of cutaneous sebaceous neoplasms at a tertiary care hospital in Pakistan. *Histopathology*, *79*, 12. https://doi.org/10.1111/his.14480

Raj, S., Katz, E., & Romagnoli, M. (2012). Aseptic meningitis associated with intravenous immunoglobulin therapy in a case of miller-fisher syndrome. *Journal of General Internal Medicine*, *27*, S400–S401. https://www.embase.com/search/results?subaction=viewrecord&id=L71297167&from=export

Ramesh, S., & Raju, S. (2015). Suprasellar arachnoid cyst presenting with bobble-head doll syndrome: Report of three cases. In *J Pediatr Neurosci* (Vol. 10, pp. 18–21). https://doi.org/10.4103/1817-1745.154321

Rayasam, S. S., Yan, F., & Agan, A. D. (2023). Head and Neck Manifestations of Fibromyalgia and Chronic Fatigue Syndrome. In *Functional Illness of the Head and Neck* (pp. 155–172). https://doi.org/10.1007/978-3-031-12998-8_16

Razack, M. S., Lore Jr, J. M., Lippes, H. A., Schaefer, D. P., & Rassael, H. (1997). Total thyroidectomy for Graves’ disease. *Head and Neck*, *19*(5), 378–383. https://doi.org/10.1002/(sici)1097-0347(199708)19:5<378::aid-hed3>3.0.co;2-x

Razak, A., Goh, B. S., Rajaran, J. R., & Nazimi, A. J. (2018). Numb chin syndrome: an ominous sign of mandibular metastasis. *BMJ Case Reports*, *2018*. https://doi.org/https://doi.org/10.1136/bcr-2017-223586

Reche, J. A., Domingo, B., Rivera, M., Stoica, B., FernÃ¡ndez, H., Corredera, E., & Toledano, N. (2011). Intermittent Horner syndrome in redundant carotid bulb. *Neuro-Ophthalmology*, *35*, S81. https://doi.org/10.3109/01658107.2011.582006

Richmon, J. D., Wang-Rodriguez, J., & Thekdi, A. A. (2009). Ehlers-Danlos syndrome presenting as dysphonia and manifesting as tongue hypermobility: Report of 2 cases. *Ear Nose Throat J*, *88*(2), E8-12.

Roberti, A., & Goffart, Y. (2015). Dyskeratosis congenita: A case report. *B-ENT*, *11*. https://www.embase.com/search/results?subaction=viewrecord&id=L625428648&from=export

Roland, L. T., Humphreys, I. M., Le, C. H., Babik, J. M., Bailey, C. E., Ediriwickrema, L. S., Fung, M., Lieberman, J. A., Magliocca, K. R., Nam, H. H., Teo, N. W., Thomas, P. C., Winegar, B. A., Birkenbeuel, J. L., David, A. P., Goshtasbi, K., Johnson, P. G., Martin, E. C., Nguyen, T. V., … Kuan, E. C. (2023). Diagnosis, Prognosticators, and Management of Acute Invasive Fungal Rhinosinusitis: Multidisciplinary Consensus Statement and Evidence-Based Review with Recommendations. *International Forum of Allergy and Rhinology*, *13*(9), 1615–1714. https://doi.org/10.1002/alr.23132

Rosenberg, R. A., Schneider, K. L., & Cohen, N. I. (1984). Head and neck presentations of acquired immunodeficiency syndrome. *Laryngoscope*, *94*(5), 642–646. https://doi.org/10.1288/00005537-198405000-00013

Rosignoli, M., Pezzuto, R. W., Galli, J., & D’Alatri, L. (1992). Midline granuloma and Wegener’s granulomatosis. *Acta otorhinolaryngologica Italica : organo ufficiale della SocietÃ  italiana di otorinolaringologia e chirurgia cervico-facciale*, *12*, 1–46. https://www.embase.com/search/results?subaction=viewrecord&id=L23833899&from=export

Sadko, K. G., Opalska-TuszyÅ„ska, A. D., SÅ‚awiÅ„ska, M., Wilkowska, A., Nowicki, R. J., Czuwara, J., & BaraÅ„ska-Rybak, W. (2022). Argyria in a patient with a delusional disorder. *Przeglad Dermatologiczny*, *109*(2). https://doi.org/10.5114/dr.2022.117986

Sanganabhatla, H. (2023). Hypoglossal Nerve Palsy - A Segmental Approach. *Annals of Indian Academy of Neurology*, *26*, S172–S173. https://www.embase.com/search/results?subaction=viewrecord&id=L642457386&from=export

Sangeetha Priya, P., Babu, A., Masthan, K. M. K., & Anitha, N. (2020). Syndromes of head and neck â€“ a quick review. *Indian Journal of Forensic Medicine and Toxicology*, *14*(4), 1412–1418. https://doi.org/10.37506/ijfmt.v14i4.11733

Savage, S. A., & Niewisch, M. R. (1993). *Dyskeratosis Congenita and Related Telomere Biology Disorders* (N. I. of H. NCBI Bookshelf. A service of the National Library of Medicine, Ed.).

Saxon, M., Snyder, H. A., & Washington Jr, J. A. (1982). Atypical Brown-Sequard syndrome following gunshot wound to the face. *Journal of Oral and Maxillofacial Surgery*, *40*(5), 299–302. https://doi.org/10.1016/0278-2391(82)90223-3

Scally, K., Ong, E., & Staines, K. S. (2010). Recurrent multiple oral warts treated successfully with systemic cidofovir. *Oral Diseases*, *16*(6), 569. https://doi.org/10.1111/j.1601-0825.2010.01743.x

Schröder, U., Schwendenwein, I., Stanclova, G., & Berger, S. (2015). Horner’s syndrome associated with neoplasia - Two case reports. *Pferdeheilkunde*, *31*(3), 228–234. https://doi.org/10.21836/PEM20150304

Searls, D. E., Pazdera, L., Korbel, E., Vysata, O., & Caplan, L. R. (2012). Symptoms and signs of posterior circulation ischemia in the new England medical center posterior circulation registry. *Arch Neurol*, *69*(3), 346–351. https://doi.org/10.1001/archneurol.2011.2083

Seitz, M. P., Minhas, S., Khouzam, A., Khouzam, N., & Harper, Y. (2021). The Face Is the Mirror of the Soul. The Cardiovascular Physical Exam Is Not Yet Dead! *CURRENT PROBLEMS IN CARDIOLOGY*, *46*(3). https://doi.org/10.1016/j.cpcardiol.2020.100644

Sepúlveda, I. A., Spencer, L. L., Mucientes, F. H., & Casanueva, F. L. (2011). Ganglioneuroma Cervical: Reporte de un Caso Cervical Ganglioneuroma: A Case Report. In *Int. J. Odontostomat* (Vol. 5, Issue 1).

Shahabuddin, H., Kiran, Z., Baloch, A. A., & Hassan, S. M. (2023). Abstract #1581409: Fahr’s Syndrome Secondary to Hypoparathyroidism in an Elderly Manâ€”A Case Report and Literature Review. *Endocrine Practice*, *29*(12), S173–S174. https://doi.org/10.1016/j.eprac.2023.10.095

Shimizu, Y., & Yagi, M. (2018). Pulsatile tinnitus and carotid artery dissection. *Auris Nasus Larynx*, *45*(1), 175–177. https://doi.org/10.1016/j.anl.2016.12.004

Silva, B., Mendes, T. F., Quaresma, M., Farinha, D., & De Sousa, N. A. (2023). EXUBERANT CUTANEOUS MANIFESTATION OF SYSTEMIC LUPUS ERYTHEMATOSUS (SLE) IN ELDERLY - A RARE FIRST MANIFESTATION. *European Journal of Case Reports in Internal Medicine*, *10*, 558. https://doi.org/10.12890/2023_V10Sup1

Silva, Y. H., Lafi, I., Krishnaiah, B., & Javed, F. (2024). Vertical Gaze Paralysis with Collier’s Sign and Contralesional Pseudo-abducens Paresis from Unilateral Dorsomedial Thalamic Stroke. *Neurology*, *102*(17). https://doi.org/10.1212/WNL.0000000000205421

Singh, R. R., Thomas, A. A., Barry, M. C., & Bouchier-Hayes, D. J. (2004). Traumatic pseudoaneurysm of the internal carotid artery presenting with oculosympathetic palsy. *Ir J Med Sci*, *173*(3), 162–163. https://doi.org/10.1007/bf03167933

Siriwardana, Y., Deepachandi, B., Gunasekara, C., Warnasooriya, W., & Karunaweera, N. D. (2019). *Leishmania donovani* Induced Cutaneous Leishmaniasis: An Insight into Atypical Clinical Variants in Sri Lanka. *JOURNAL OF TROPICAL MEDICINE*, *2019*. https://doi.org/10.1155/2019/4538597

Soares Dos Reis, R., Rocha, A. L., & Castro, P. (2019). Numb chin syndrome caused by a solitary fibrous tumour. *European Journal of Neurology*, *26*, 917. https://doi.org/10.1111/ene.14019

Soga, F., Wada, T., Inoue, Y., Hada, F., Fujita, A., & Horinaka, A. (2017). A case of malignant sympathetic paraganglioma presenting with Horner syndrome. *Practica Oto-Rhino-Laryngologica*, *110*(1), 25–30. https://doi.org/10.5631/jibirin.110.25

Spalatin, J., Hanson, R. P., & Jones, T. D. (1973). Edema of the eyelid and face of chickens exposed in the viscerotropic type of Newcastle disease virus. *Avian Diseases*, *17*(3), 623–628. https://doi.org/10.2307/1589163

Spitze, A., Chao, D., Al-Zubidi, N., Yalamanchili, S., & Lee, A. G. (2014). Anisocoria — abnormally small pupil (horner syndrome). In *Questions and Answers in Neuro-Ophthalmology: A Case-Based Approach* (pp. 1–12). https://doi.org/10.1142/9789814578783_0001

Stafford, R., Sonis, S., Lockhart, P., & Sonis, A. (1980). Oral pathoses as diagnostic indicators in leukemia. *Oral Surg Oral Med Oral Pathol*, *50*(2), 134–139. https://doi.org/10.1016/0030-4220(80)90200-5

Standefers Jr, J. A., & Mattox, D. E. (1986). Head and neck manifestations of collagen vascular diseases. *Otolaryngologic Clinics of North America*, *19*(1), 181–210. https://www.embase.com/search/results?subaction=viewrecord&id=L16182711&from=export

Stavridopoulos, M. T., Nikitopoulos, S. N., Oikonomou, A. A., & Tsiouma, G. K. (2023). A Case of Idiopathic First Bite Syndrome, Possibly Linked to Type I Diabetes Mellitus. *Cureus*, *15*(3), e36710. https://doi.org/10.7759/cureus.36710

Stupak, H. D., Scheuller, M. C., Schindler, D. N., & Ellison, D. E. (2003). Tularemia of the head and neck: a possible sign of bioterrorism. *Ear Nose Throat J*, *82*(4), 263–265.

Sulman, A., Kaplan, J., & Hatahet, M. (2021). VISIONS ARE SELDOM ALL THEY SEEM: UNMASKED ADIE’S PUPIL IN PATIENTS UNDER SEDATION. *Chest*, *160*(4), A862. https://doi.org/10.1016/j.chest.2021.07.808

Suzuki, M., Makri, A., Gupta, G., Araque, K., Kleess, L., Raygada, M., Lodish, M. B., Stratakis, C. A., & Pacak, K. (2017). A family with carney stratakis syndrome with succinate dehydrogenase complex subunit d mutation. *Endocrine Reviews*, *38*(3). https://www.embase.com/search/results?subaction=viewrecord&id=L617152054&from=export

Szymczyk, K., MisioÅ‚ek, M., Kubik, P., & ZgÅ‚obisz-Kozik, H. (1992). Neurofibromatosis of the head and neck. *WiadomoÅ›ci lekarskie (Warsaw, Poland : 1960)*, *45*(5), 218–219. https://www.embase.com/search/results?subaction=viewrecord&id=L23816187&from=export

Takahashi, T., Murase, T., & Isayama, Y. (1982). Rhabdomyosarcoma presenting as Garcin’s syndrome. *Surgical Neurology*, *17*(4), 269–272. https://doi.org/10.1016/0090-3019(82)90119-7

Takayama, R., Ueno, T., & Saeki, H. (2017). Immunoglobulin G4-related disease and its skin manifestations. *The Journal of Dermatology*, *44*(3), 288–296. https://doi.org/https://doi.org/10.1111/1346-8138.13723

Talebzadeh, A. T., & Talebzadeh, N. (2023). Facial Presentation of Crohn’s Disease: Report of a Case. *Cureus*, *15*(3), e36024. https://doi.org/10.7759/cureus.36024

Taveirne, M., & Lucker, G. (2007). Neonatal lupus erythematosus. *Nederlands Tijdschrift voor Dermatologie en Venereologie*, *17*(2), 66–69. https://www.embase.com/search/results?subaction=viewrecord&id=L46332937&from=export

Thurkintavide, M. R., Vinay, K., Aggarwal, D., Das Radotra, B., Kumaran, M. S., & Parsad, D. (2020). Peri-orbital acquired dermal macular hyperpigmentation: A distinctive clinical entity in young adults-casecontrol study. *Journal of the Dermatology Nurses’ Association*, *12*(2). https://www.embase.com/search/results?subaction=viewrecord&id=L634430795&from=export

Uh, J. A., Lee, S. K., Lee, U. H., & Kim, M. S. (2023). Analysis of the Clinical Features of Late Onset Vitiligo. *Korean Journal of Dermatology*, *61*(10), 595–601. https://www.scopus.com/inward/record.uri?eid=2-s2.0-85187275322&partnerID=40&md5=bf16164ccd7f359f466d19aed6d88677

Uslu, M., Sendur, N., Savk, E., Karaman, G., Tosun, A., & Tataroglu, C. (2012). Phacomatosis pigmento keratotica with musculoskeleta l abnormality: A case report. *European Journal of Pediatric Dermatology*, *22*(1), 78. https://www.embase.com/search/results?subaction=viewrecord&id=L70795521&from=export

Verriello, L., Dalla Torre, C., Driussi, M., Pauletto, G., Imazio, M., & Briani, C. (2023). BULBAR SYNDROME AS ATYPICAL PRESENTATION OF TRANSTHYRETIN FAMILIAL AMYLOID NEUROPATHY ASSOCIATED WITH FIRST BITE SYNDROME: POSSIBLE CAUSAL RELATIONSHIP. *Journal of the Peripheral Nervous System*, *28*, S50–S51. https://doi.org/10.1111/jns.12550

Vithana, S. M. P., & Rajakaruna, R. C. B. (2023). A Rare Case of Double Pyramidal Lobe of the Thyroid Gland. *Ear Nose Throat J*, 1455613231152086. https://doi.org/10.1177/01455613231152086

Vlastarakos, P. V, Menelaou, C., & Thrasyvoulou, G. (2024). Horner’s syndrome as an unusual complication of retropharyngeal schwannoma excision. *HIPPOKRATIA*, *28*(1), 41–42.

Wang, S. J., Liu, J. C., & Califano, J. (2016). Vexing oral cavity lesions. *Otolaryngology - Head and Neck Surgery (United States)*, *155*, P17. https://doi.org/10.1177/0194599816654888c

Whizar-Lugo, V. M., Calvo-Soto, P., Anzorena-Vallarino, F., & Preciado-Ramírez, S. (2006). Ramsay-Hunt syndrome. *Anestesia En Mexico*, *18*(3), 170–173. https://www.scopus.com/inward/record.uri?eid=2-s2.0-77950454180&partnerID=40&md5=a112fcf74b2b70404e657b32a2f16673

Williams, J., O’Connell, K., Kinsella, J., Curran, A., Crotty, T., & McGuigan, C. (2013). Octreotide positive carotid paragangliomas causing progressive cranial neuropathies. *Neurology*, *80*(1). https://www.embase.com/search/results?subaction=viewrecord&id=L71131343&from=export

Yaghoobi, R., Feily, A., Behrooz, B., Yaghoobi, E., & Mokhtarzadeh, S. (2010). Palpebral involvement as a presenting and sole manifestation of discoid lupus erythematosus. *ScientificWorldJournal*, *10*, 2130–2131. https://doi.org/10.1100/tsw.2010.209

Yamagiwa, M., Miyoshi, Y., Sakakura, Y., Nishioka, H., Inagaki, M., Kanamaru, I., Ohyama, M., & Morikawa, K. (1981). A Clinical Study of Facial Palsy. *Practica Otologica*, *74*, 928–937. https://doi.org/10.5631/jibirin.74.5special_928

Yamauchi, Y., Kobayashi, T., Nagaro, T., Yamamoto, H., Kimura, S., & Arai, T. (1994). A case of hemifacial hyperhidrosis on the opposite side of the Pancoast tumor. *Japanese Journal of Anesthesiology*, *43*(6), 924–926. https://www.scopus.com/inward/record.uri?eid=2-s2.0-0028244272&partnerID=40&md5=8fd39355310f3b0450e055bac8cbab56

Yang, M. C., Hsu, Y. H., Liu, D. W., & Chou, Y. F. (2009). AIDS-related Kaposi’s Sarcoma of the Nasopharynx. *Tzu Chi Medical Journal*, *21*(4), 342–344. https://doi.org/10.1016/S1016-3190(09)60068-9

Yeh, H. F., Seak, C. J., Chiu, T. F., & Chang, Y. C. (2009). Traumatic vertebral artery dissection and Wallenberg syndrome after a motorcycle collision. *American Journal of Emergency Medicine*, *27*(1), 131.e1-131.e3. https://doi.org/10.1016/j.ajem.2008.04.025

Yen, P. T., Lee, F. P., & Huang, T. S. (1991). von Recklinghausen’s disease with involvement of right parapharyngeal space: report of a case. *J Formos Med Assoc*, *90*(10), 1018–1021.

Yoskovitch, A., Tewfik, T. L., Brouillette, R. T., Schloss, M. D., & Der Kaloustian, V. M. (1998). Acute airway obstruction in Hunter syndrome. *Int J Pediatr Otorhinolaryngol*, *44*(3), 273–278. https://doi.org/10.1016/s0165-5876(98)00063-9

Yu, D. X., Pi, S. J., & Zhang, W. S. (2013). [Clinical manifestation of Kaposi sarcoma in otorhinolaryngology head and neck surgery]. *Zhonghua Er Bi Yan Hou Tou Jing Wai Ke Za Zhi*, *48*(3), 241–243.

Zhao, C. Y., Chiang, N. Y. Z., & Murrell, D. F. (2015). Neonatal autoimmune blistering diseases-a systematic review of literature. *Australasian Journal of Dermatology*, *56*, 9. https://doi.org/10.1111/ajd.12337
