# Supplementary material for: Head and Neck Clinical Signs Associated With Diseases: A Scoping Review
Source: Spec Care Dentist. 2026 May 14;46:e70185. doi: 10.1111/scd.70185 (PMC13176508; doi:10.1111/scd.70185)
Supplement: Supplementary file 4 — Supplementary Material 4: Excluded Studies and Exclusion Criteria From Grey Literature. [file SCD-46-0-s001.docx]

**Supplementary Material 4**. Excluded studies and exclusion criteria from grey literature

| **Reference** | **Author, year** | **Reason for exclusion*** |
| --- | --- | --- |
|  | (Abdullah et al., 1974) | 2 |
|  | (Abdulrahman & Al Samawi, 2021) | 2 |
|  | (Agarwal et al., 2014) | 2 |
|  | (Aggarwal, 2018) | 2 |
|  | (Akhtar et al., 2019) | 2 |
|  | (Alghamdi, 2008) | 2 |
|  | (Ali Khan et al., 2024) | 2 |
|  | (Anonymous, 2010) | 2 |
|  | (Arikan et al., 2012) | 5 |
|  | (Armstrong & Okun, 2020) | 2 |
|  | (Azrielant et al., 2021) | 2 |
|  | (Baatz et al., 2022) | 2 |
|  | (Bachmeyer, 2012) | 5 |
|  | (Banat et al., 2021) | 2 |
|  | (Bento & dos Santos, 2015) | 2 |
|  | (Bird et al., 2001) | 2 |
|  | (Brancati et al., 2010) | 6 |
|  | (Brigo et al., 2022) | 2 |
|  | (Brodell & Bukavina, 2014) | 2 |
|  | (Bruel et al., 2018) | 2 |
|  | (Bui et al., 2020) | 2 |
|  | (Cardona & Efron, 1996) | 2 |
|  | (Carvalho et al., 2021) | 2 |
|  | (Castilho et al., 2023) | 5 |
|  | (Chai et al., 2024) | 3 |
|  | (Chan et al., 2020) | 5 |
|  | (Chi et al., 2010) | 2 |
|  | (Chou, 2020) | 6 |
|  | (Colarusso et al., 2010) | 2 |
|  | (Coleman et al., 2020) | 2 |
|  | (Cousins & Harikrishnan, 2017) | 5 |
|  | (Cox-Brinkman et al., 2007) | 2 |
|  | (Crow et al., 1998) | 2 |
|  | (da Cunha & Alvarez, 2018) | 2 |
|  | (Davatchi et al., 2014) | 5 |
|  | (Davies, 1996) | 6 |
|  | (Dennison et al., 2019) | 2 |
|  | (Domínguez-Casas et al., 2024) | 2 |
|  | (Dupé et al., 2022) | 2 |
|  | (Eckhardt et al., 2014) | 6 |
|  | (El-Reshaid et al., 2021) | 2 |
|  | (Figlus et al., 2014) | 2 |
|  | (Fischer, 2007) | 5 |
|  | (Fisher, 2001) | 6 |
|  | (Forsyth et al., 2022) | 3 |
|  | (Foucar et al., 1990) | 6 |
|  | (Fusco & Giustina, 2008) | 2 |
|  | (Gaddey, 2017) | 2 |
|  | (Garcia-Doval et al., 2000) | 2 |
|  | (George et al., 2021) | 2 |
|  | (Gerfaud-Valentin et al., 2014) | 2 |
|  | (Ghiam et al., 2024) | 2 |
|  | (Gibson & Wray, 1998) | 2 |
|  | (Gonzalez et al., 2014) | 5 |
|  | (Gracey & Balladares, 2023) | 2 |
|  | (Gupta et al., 2014) | 2 |
|  | (Haehner et al., 2007) | 2 |
|  | (Hale et al., 2006) | 2 |
|  | (Hamaguchi et al., 2013) | 2 |
|  | (Hanane et al., 2024) | 5 |
|  | (He et al., 2022) | 2 |
|  | (Hertz et al., 2017) | 2 |
|  | (Hodgson, 2001) | 5 |
|  | (Hsin-Chien et al., 2007) | 3 |
|  | (Hupert, 1984) | 2 |
|  | (Hyams et al., 1991) | 6 |
|  | (Inazawa-Terada et al., 2022) | 3 |
|  | (Iscan et al., 2005) | 2 |
|  | (Ito et al., 2013) | 6 |
|  | (J. Hu et al., 2019) | 6 |
|  | (Jacome, 1991) | 2 |
|  | (Jacome, 2001) | 2 |
|  | (Jang et al., 2023) | 5 |
|  | (Jatania & K, 2012) | 2 |
|  | (Jesuthasan et al., 2022) | 2 |
|  | (Joshi et al., 2018) | 3 |
|  | (Jung-Rern et al., 2003) | 2 |
|  | (Kajal et al., 2013) | 2 |
|  | (Kamoun et al., 2014) | 5 |
|  | (Karadan et al., 2018) | 2 |
|  | (Kasravi et al., 2010) | 2 |
|  | (Kim et al., 2015) | 2 |
|  | (Kiranmai, 2005) | 2 |
|  | (Kishi et al., 2020) | 2 |
|  | (Kister et al., 2010) | 3 |
|  | (Knibbs, 2021) | 5 |
|  | (Kravos & Boltezar, 2010) | 2 |
|  | (Krishnan et al., 2010) | 2 |
|  | (Kshirsagar & Ingale, 2014) | 2 |
|  | (Kubik et al., 2012) | 2 |
|  | (Kubik et al., 2012)) | 5 |
|  | (Lawley, 2008) | 5 |
|  | (Lei et al., 2023) | 3 |
|  | (Leon et al., 2020) | 2 |
|  | (LeWit, 2017) | 3 |
|  | (Lin et al., 2022) | 2 |
|  | (L(Lin et al., 2022)nska, 2(Lis-Swiety & Brzezinska, 2020)Click or tap here to enter text. | 2 |
|  | (Lopez et al., 2014)12) | 2 |
|  | (Mansouri et al., 2020) | 2 |
|  | (Marqusee et al., 2000) | 2 |
|  | (Marshall, 2004) | 2 |
|  | (Martinez-Molina et al., 2024) | 2 |
|  | (Mass, 2012) | 2 |
|  | (Mavrikakis, 2011) | 6 |
|  | (Melish & Glasgow, 1970) | 1 |
|  | (Melmon & Rosen, 1964) | 2 |
|  | (Mercier et al., 2019) | 2 |
|  | (Mermerkaya et al., 2014) | 2 |
|  | (Miest et al., 2017) | 2 |
|  | (Migliaccio et al., 2004) | 2 |
|  | (Moon et al., 2022) | 5 |
|  | (Morrison et al., 1997) | 2 |
|  | (Mourits et al., 1989) | 2 |
|  | (Mulazimoglu et al., 2017) | 2 |
|  | (Mulla et al., 2012) | 5 |
|  | (Nance & Berry, 1992) | 2 |
|  | (Nerurkar & Sheth, 2017) | 3 |
|  | (Nishizawa et al., 2021) | 2 |
|  | (Nystrom et al., 2009) | 2 |
|  | (Odaka et al., 2003) | 2 |
|  | (Ohashi et al., 2020) | 2 |
|  | (Ohko et al., 2020) | 2 |
|  | (Ohlson, 2018) | 3 |
|  | (Ouattara et al., 2018) | 2 |
|  | (Ozekmekçi et al., 2007) | 2 |
|  | (Pagella et al., 2014) | 2 |
|  | (Pamnani et al., 2022) | 2 |
|  | (Panchangam et al., 2021) | 2 |
|  | (Panchaprateep et al., 2020) | 2 |
|  | (Parajuli & Shrestha, 2022) | 2 |
|  | (Pasquini et al., 2019) | 2 |
|  | (Patra et al., 2013) | 2 |
|  | (Patterson et al., 2012) | 2 |
|  | (Pavone et al., 1999) | 2 |
|  | (Pearce, 2005) | 3 |
|  | (Pedraza et al., 2015) | 2 |
|  | (Persily & Collins, 2019) | 2 |
|  | (Pinto-Almeida et al., 2013) | 2 |
|  | (Pirola et al., 2022) | 2 |
|  | (Polat et al., 2018) | 2 |
|  | (Ponsen et al., 2004) | 2 |
|  | (Pratiksha Shankarlal, 2022) | 5 |
|  | (Prieur et al., 1987) | 2 |
|  | (Putoux et al., 2012) | 2 |
|  | (Pyykko et al., 2012) | 2 |
|  | (Rafailidis et al., 2007) | 2 |
|  | (Rana & Wadia, 1985) | 2 |
|  | (Renee H. Grau, 2007) | 2 |
|  | (Rijnenberg et al., 2024) | 5 |
|  | (Rossi et al., 2016) | 2 |
|  | (Saito et al., 2022) | 2 |
|  | (Saniasiaya, 2022) | 2 |
|  | (Schott & Rossor, 2016) | 2 |
|  | (Schweiger et al., 2021) | 2 |
|  | (Sen et al., 2014) | 2 |
|  | (Sévin et al., 2007) | 2 |
|  | (Sidell et al., 2006) | 5 |
|  | (Silva Riveiro et al., 2018) | 5 |
|  | (Sivesind et al., 2022) | 2 |
|  | (Smith et al., 2002) | 2 |
|  | (Solomon & Muenke, 2012) | 2 |
|  | (Sone et al., 2024) | 2 |
|  | (Strong et al., 2023) | 2 |
|  | (Syrine et al., 2023) | 5 |
|  | (Takayama et al., 2017) | 2 |
|  | (Tanimura & Terakawa, 2023) | 2 |
|  | (Unknown, 1998) | 5 |
|  | (Unknown, 2001) | 5 |
|  | (Urushitani et al., 1995) | 2 |
|  | (Valenzuela et al., 2022) | 2 |
|  | (Viswanath & Aner, 2018) | 2 |
|  | (Wai Foong et al., 2018) | 5 |
|  | (Wang et al., 2023) | 2 |
|  | (Wheeler et al., 1995) | 3 |
|  | (Whybra et al., 2001) | 2 |
|  | (Xiang et al., 2014) | 5 |
|  | (Xiang et al., 2014) | 2 |
|  | (Y. Hu et al., 2024) | 2 |
|  | (Young Il, 2014) | 2 |
|  | (Yuki et al., 2004) | 5 |
|  | (Zadro et al., 2008) | 2 |
|  | (Zaidan et al., 2013) | 5 |
|  | (Zielińska & Zielińska, 2019) | 2 |
|  | (Zivkovic et al., 2022) | 2 |
|  | (Zoubolis, 1999) | 6 |

***Reasons for exclusion:**

1. Studies not involving human participants;

2. Clinical signs observed other than head and neck region or not specific of the disease;

3. Studies that did not involve physical examination or physical examination performed with auxiliary equipment;

4. Studies conducted outside clinical or medical examination settings, such as community health surveys;

5. Books, conference abstracts, opinion articles, technique articles, posters, guidelines, and reviews that did not specifically address clinical signs in the head and neck region as a primary focus;

6. Full-text access unavailable, despite efforts to contact the corresponding authors.

**References**

Abdullah, A. F., Ditto, E. W., Byrd, E. B., & Williams, R. (1974). Extreme-lateral lumbar disc herniations: clinical syndrome and special problems of diagnosis. *Journal of Neurosurgery*, *41*(2), 229–234.

Abdulrahman, F. A., & Al Samawi, M. S. (2021). A case of post COVIDâ€19 multisystem inflammatory syndrome and Bell’s palsy in a young adult. *Clinical Case Reports*, *9*(9). https://doi.org/https://doi.org/10.1002/ccr3.4801

Agarwal, S., Ojha, A., & Gupta, S. (2014). Profile of vitiligo in Kumaun region of Uttarakhand, India. *Indian Journal of Dermatology*, *59*(2), 209. https://doi.org/https://doi.org/10.4103/0019-5154.127706

Aggarwal, V. R. (2018). The Role of Oral Health in Complex Emergencies and Disaster Rehabilitation Medicine. *Disaster Medicine and Public Health Preparedness*, *12*(6), 772–777. https://doi.org/https://doi.org/10.1017/dmp.2018.2

Akhtar, A., Hassan Syed, A., Falah, N. U., Khan, M., & Sheikh, F. N. (2019). Joubert Syndrome: A Rare Radiological Case. *Cureus*, *11*(12). https://doi.org/https://doi.org/10.7759/cureus.6410

Alghamdi, Y. (2008). Skin tags as a presenting sign of basal cell nevus syndrome in three sisters of the same family. *Annals of Saudi Medicine*, *28*(2), 132–134. https://doi.org/https://doi.org/10.4103/0256-4947.51751

Ali Khan, Q., Levin-Carrion, Y., Khan, R., Khan, A. Z., Saddiq, S., Vaishnavi, G., Nadella, A., Kooner, A., Ayiz, J., & Farrukh, A. M. (2024). Hashimotoâ€™s Thyroiditis in Noonan Syndrome: A Case Report. *Cureus*, *16*(1). https://doi.org/https://doi.org/10.7759/cureus.51592

Anonymous. (2010). PULSE CLINICAL: Non-itchy facial rash. *Pulse*, 29. https://www.proquest.com/trade-journals/pulse-clinical-non-itchy-facial-rash/docview/233382355/se-2?accountid=8113

Arikan, F. I., Ã–zkan, F., Ag ras, P. I., Zengin, T., catakli, T., & Bilge, Y. D. (2012). 513 HeerfordtÂ´S Syndrom in an Adolescent Boy. *Archives of Disease in Childhood, Suppl. 2*, *97*. https://doi.org/https://doi.org/10.1136/archdischild-2012-302724.0513

Armstrong, M. J., & Okun, M. S. (2020). Diagnosis and treatment of Parkinson disease: a review. *Jama*, *323*(6), 548–560.

Azrielant, S., Ellenbogen, E., Peled, A., Zemser-Werner, V., Samuelov, L., Sprecher, E., & Pavlovsky, M. (2021). Diffuse Facial Hyperpigmentation as a Presenting Sign of Lupus Erythematosus: Three Cases and Review of the Literature. *Case Reports in Dermatology*, *13*(2), 263–270. https://www.proquest.com/scholarly-journals/diffuse-facial-hyperpigmentation-as-presenting/docview/2570443829/se-2?accountid=8113

Baatz, M., Holley, H. L., Ahlert, J., & Rubin, M. J. (2022). Staphylococcal Scalded Skin Syndrome in a Ten-Month-Old Male. *Cureus*, *14*(7). https://doi.org/https://doi.org/10.7759/cureus.26975

Bachmeyer, C. (2012). Acquired facial atrophy: A neglected clinical sign of POEMS syndrome. *American Journal of Hematology*, *87*(1), 131. https://doi.org/https://doi.org/10.1002/ajh.22204

Banat, O., Albataineh, T., & Hashem, H. (2021). Numb chin syndrome as a presentation for acute myeloid leukemia relapse post–hematopoietic cell transplant: Case report and review of literature. *Pediatric Transplantation*, *25*(5). https://doi.org/10.1111/petr.13841

Bento, M. E. de M. M. B., & dos Santos, J. A. M. M. (2015). *Tratamento mÃ©dico-dentÃ¡rio do paciente portador de esclerose mÃ^o^ltipla* (Issue 31000894). ["https://www.proquest.com/dissertations-theses/tratamento-mÃ©dico-dentÃ¡rio-do-paciente-portador/docview/2905730979/se-2?accountid=8113", “http://hdl.handle.net/10400.26/11719”]

Bird, P. A., Oakley, S. P., Shnier, R., & Kirkham, B. W. (2001). Prospective evaluation of magnetic resonance imaging and physical examination findings in patients with greater trochanteric pain syndrome. *Arthritis & Rheumatism: Official Journal of the American College of Rheumatology*, *44*(9), 2138–2145.

Brancati, F., Dallapiccola, B., & Valente, E. M. (2010). Joubert Syndrome and related disorders. *Orphanet Journal of Rare Diseases*, *5*, 1–10.

Brigo, F., Lorusso, L., Carta, S., Gianeto, C., Sperone, A., Ginesci, R., Orioli, A., Benna, P., & Labate, C. R. (2022). Camillo Negro (1861â€“1927) and the â€œbulbo-palpebral hyperkinetic phenomenonâ€ in peripheral facial palsy. *Neurological Sciences*, *43*(3), 2149–2152. https://doi.org/https://doi.org/10.1007/s10072-021-05436-3

Brodell, R. T., & Bukavina, L. (2014). An Acute Vesiculobullous Rash on the Face. *American Family Physician*, *89*(6), 473–474. https://www.proquest.com/scholarly-journals/acute-vesiculobullous-rash-on-face/docview/2454405374/se-2?accountid=8113

Bruel, A. L., Levy, J., Elenga, N., Defo, A., Favre, A., Lucron, H., Capri, Y., Perrin, L., Passemard, S., Vial, Y., Tabet, A. C., Faivre, L., Thauvin-Robinet, C., & Verloes, A. (2018). INTU-related oral-facial-digital syndrome type VI: A confirmatory report. *Clinical Genetics*, *93*(6), 1205–1209. https://doi.org/10.1111/cge.13238

Bui, T. P. H., Nguyen, N. T., Ngo, V. D., Nguyen, H.-N., Ly, T. T. H., Do, H. D., & Huynh, M.-T. (2020). Novel compound heterozygous TMEM67 variants in a Vietnamese family with Joubert syndrome: a case report. *BMC Medical Genetics*, *21*, 1. https://doi.org/https://doi.org/10.1186/s12881-020-0962-0

Cardona, G., & Efron, N. (1996). *Ocular and Facial Thermography in Herpes Zoster Ophthalmicus and Postherpetic Neuralgia* (Issue 29951775). https://www.proquest.com/dissertations-theses/ocular-facial-thermography-herpes-zoster/docview/2718965763/se-2?accountid=8113

Carvalho, H. I. M., da Costa Ferreira Torres, T., & da Cunha Velho, G. M. C. (2021). *RosÃ¡cea Helena Isabel Moreira Carvalho* (Issue 30974263). https://www.proquest.com/dissertations-theses/rosÃ¡cea-helena-isabel-moreira-carvalho/docview/3059347958/se-2?accountid=8113

Castilho, N. L., Resende, K. K. M., Santos, J. A. dos, Machado, R. A., Coletta, R. D., Guerra, E. N. S., Acevedo, A. C., & Martelli-Junior, H. (2023). Oligodontia in the Clinical Spectrum of Syndromes: A Systematic Review. In *Dentistry Journal* (Vol. 11, Issue 12). Multidisciplinary Digital Publishing Institute (MDPI). https://doi.org/10.3390/dj11120279

Chai, X., Qian, X., Wang, H., Zhou, Z., Tang, L., Zhang, J., & Longfei, Y. (2024). Comparative analysis of free SMAS fold flap and ADM in facial depression after parotidectomy. *BMC Surgery*, *24*, 1–8. https://doi.org/https://doi.org/10.1186/s12893-024-02670-2

Chan, J. L., Ebadi, H., & Sarna, J. R. (2020). Guillain-Barré Syndrome with Facial Diplegia Related to SARS-CoV-2 Infection. In *Canadian Journal of Neurological Sciences* (Vol. 47, Issue 6, pp. 852–854). Cambridge University Press. https://doi.org/10.1017/cjn.2020.106

Chen, H.-C., Jen, Y.-M., Wang, C.-H., Lee, J.-C., & Lin, Y.-S. (2007). Etiology of Vocal Cord Paralysis. *ORL : Journal for Oto - Rhino - Laryngology and Its Related Specialties*, *69*(3), 167–171. https://www.proquest.com/scholarly-journals/etiology-vocal-cord-paralysis/docview/225069962/se-2?accountid=8113

Chi, A. C., Neville, B. W., Krayer, J. W., & Gonsalves, W. C. (2010). Oral manifestations of systemic disease. *American Family Physician*, *82*(11), 1381–1388.

Chou, K. L. (2020). Clinical manifestations of Parkinson disease. *UpToDate, Fev*.

Colarusso, G., Gambineri, E., Lapi, E., Casini, T., Tucci, F., Lippi, F., & Azzari, C. (2010). Evans syndrome and antibody deficiency: an atypical presentation of chromosome 22q11.2 deletion syndrome. *Pediatric Reports*, *2*(2). https://doi.org/https://doi.org/10.4081/pr.2010.e13

Coleman, I., Ruiz, G., Brahmbhatt, S., & Ackerman, L. (2020). Acute generalized exanthematous pustulosis and Stevens-Johnson syndrome overlap due to hydroxychloroquine: a case report. *Journal of Medical Case Reports*, *14*, 1–4. https://doi.org/https://doi.org/10.1186/s13256-020-02504-8

Cousins, O., & Harikrishnan, S. (2017). PO121 Continuous facial myokymia as the presenting symptom of multiple sclerosis. *Journal of Neurology, Neurosurgery and Psychiatry, Suppl. Supplement 1*, *88*. https://doi.org/https://doi.org/10.1136/jnnp-2017-ABN.151

Cox-Brinkman, J., Vedder, A., Hollak, C., Richfield, L., Mehta, A., Orteu, K., Wijburg, F., & Hammond, P. (2007). Three-dimensional face shape in Fabry disease. *European Journal of Human Genetics : EJHG*, *15*(5), 535–542. https://doi.org/https://doi.org/10.1038/sj.ejhg.5201798

Crow, Y. J., Zuberi, S. M., McWilliam, R., Tolmie, J. L., Hollman, A., Pohl, K., & Stephenson, J. B. (1998). “Cataplexy” and muscle ultrasound abnormalities in Coffin-Lowry syndrome. *Journal of Medical Genetics*, *35*(2), 94. https://doi.org/https://doi.org/10.1136/jmg.35.2.94

da Cunha, S. C. N., & Alvarez, F. (2018). *Paralisia Facial PerifÃ©rica: DiagnÃ^3^stico e Tratamento* (Issue 28934150). https://www.proquest.com/dissertations-theses/paralisia-facial-perifÃ©rica-diagnÃ^3^stico-e/docview/2628778784/se-2?accountid=8113

Davatchi, F., Assaad‐Khalil, S., Calamia, K. T., Crook, J. E., Sadeghi‐Abdollahi, B., Schirmer, M., Tzellos, T., Zouboulis, C. C., & Akhlagi, M. (2014). The International Criteria for Behçet’s Disease (ICBD): a collaborative study of 27 countries on the sensitivity and specificity of the new criteria. *Journal of the European Academy of Dermatology and Venereology*, *28*(3), 338–347.

Davies, T. F. (1996). The pathogenesis of Graves’ disease. *Werner and Ingbar’s the Thyroid: A Fundamental and Clinical Text. Edited by Braverman LE, Utiger RD. Philadelphia: Lippincott-Raven*, 525–536.

Dennison, R., Novak, C., Rebman, A., Venkatesan, A., & Aucott, J. (2019). Lyme Disease with Erythema Migrans and Seventh Nerve Palsy in an African-American Man. *Cureus*, *11*(12). https://doi.org/https://doi.org/10.7759/cureus.6509

Domínguez-Casas, L. C., Lasa-Teja, C., Ferraz-Amaro, I., Castañeda, S., & Blanco, R. (2024). Increased Risk of Herpes Zoster in Rheumatoid Arthritis Not Only Due to JAK Inhibitors—Study of 392 Patients from Single University Center. *Journal of Clinical Medicine*, *13*(11). https://doi.org/10.3390/jcm13113121

Dupé, C., Lefeuvre, C., Solé, G., Behin, A., Pottier, C., Duval, F., Carlier, R. Y., Prigent, H., Lacau St Guily, J., Arrassi, A., Taouagh, N., Hamroun, D., Nicolas, G., & Laforêt, P. (2022). Macroglossia: A potentially severe complication of late-onset Pompe disease. *European Journal of Neurology*, *29*(7), 2121–2128. https://doi.org/10.1111/ene.15330

Eckhardt, R. B., Henneberg, M., Weller, A. S., & HsÃ¼, K. J. (2014). Rare events in earth history include the LB1 human skeleton from Flores, Indonesia, as a developmental singularity, not a unique taxon. *Proceedings of the National Academy of Sciences of the United States of America*, *111*(33), 11961. https://www.proquest.com/scholarly-journals/rare-events-earth-history-include-lb1-human/docview/1556006549/se-2?accountid=8113

El-Reshaid, K., Al-Bader, S., & Hossameldin Tawfik, S. (2021). A Self-Limited Facial Rash in a Lupus Patient: The Case of Primary Facial Raynaudâ€™s Phenomenon. *Case Reports in Dermatology*, *13*(2), 366–371. https://www.proquest.com/scholarly-journals/self-limited-facial-rash-lupus-patient-case/docview/2570443391/se-2?accountid=8113

Figlus, M., PaweÅ‚czyk, M., Kacperska, M. J., Janiak, S., & JastrzÄ™bski, K. (2014). Recurrent facial nerve paralysis â€“ case report. *Current Neurology*, *14*(2), 134-134â€“139. https://doi.org/https://doi.org/10.15557/AN.2014.0015

Fischer, D. A. (2007). Seasonal Palatal Pruritus is a Risk Factor for Oral Allergy Syndrome. *Journal of Allergy and Clinical Immunology, Suppl. S*, *119*(1). https://doi.org/https://doi.org/10.1016/j.jaci.2006.12.128

Fisher, K. (2001). Wasting and lipodystrophy in patients infected with HIV: a practical approach in clinical practice. *The AIDS Reader*, *11*(3), 132–133, 137–140, 147. https://www.proquest.com/trade-journals/wasting-lipodystrophy-patients-infected-with-hiv/docview/194673082/se-2?accountid=8113

Forsyth, R. L., Parisi, M. A., Altintas, B., Malicdan, M. C., Vilboux, T., Knoll, J., Brooks, B. P., Zein, W. M., Gahl, W. A., Toro, C., & Gunay-Aygun, M. (2022). Systematic analysis of physical examination characteristics of 94 individuals with Joubert syndrome: Keys to suspecting the diagnosis. *American Journal of Medical Genetics, Part C: Seminars in Medical Genetics*, *190*(1), 121–130. https://doi.org/10.1002/ajmg.c.31966

Foucar, E., Rosai, J., & Dorfman, R. (1990). Sinus histiocytosis with massive lymphadenopathy (Rosai-Dorfman disease): review of the entity. *Seminars in Diagnostic Pathology*, *7*(1), 19–73.

Fusco, C., & Giustina, E. Della. (2008). Relapsing peripheral facial palsy: An unusual early sign of Friedreich ataxia: JPN. *Journal of Pediatric Neurology*, *6*(2), 151–153. https://www.proquest.com/scholarly-journals/relapsing-peripheral-facial-palsy-unusual-early/docview/217673100/se-2?accountid=8113

Gaddey, H. L. (2017). Oral manifestations of systemic disease. *General Dentistry*, *65*(6), 23–29.

Garcia-Doval, I., LeCleach, L., Bocquet, H., Otero, X.-L., & Roujeau, J.-C. (2000). Toxic epidermal necrolysis and Stevens-Johnson syndrome: does early withdrawal of causative drugs decrease the risk of death? *Archives of Dermatology*, *136*(3), 323–327.

George, R. S., Sadler, R. M., & Clarke, D. B. (2021). An Unusual Todd’s Phenomenon: Post-Ictal Prosopagnosia. In *Canadian Journal of Neurological Sciences* (Vol. 48, Issue 5, pp. 730–731). Cambridge University Press. https://doi.org/10.1017/cjn.2020.264

Gerfaud-Valentin, M., Jamilloux, Y., Iwaz, J., & Sève, P. (2014). Adult-onset Still’s disease. *Autoimmunity Reviews*, *13*(7), 708–722.

Ghiam, S., Sojitra, B., Reiff, C., Sears, C. M., & Karlin, J. N. (2024). Perceived Conjunctival Foreign Material Egress in Morgellons Disease: A Case Study. *Case Reports in Psychiatry*, *2024*. https://doi.org/10.1155/2024/9952722

Gibson, J., & Wray, D. (1998). *Orofacial Granulomatosis: Clinical and Immunological Studies* (Issue 11007711). https://www.proquest.com/dissertations-theses/orofacial-granulomatosis-clinical-immunological/docview/2126658870/se-2?accountid=8113

Gonzalez, L., Gonzalez, J. R., Santos, E., Gonzalez, M., Valle, L., & De la Fuente, J. (2014). THU0388 Demyelinating Lesions in BehcEt’s Diseas as A Form of Parenchymal Involvement. *Annals of the Rheumatic Diseases, Suppl. 2*, *73*, 316. https://doi.org/https://doi.org/10.1136/annrheumdis-2014-eular.4671

Gracey, C., & Balladares, R. (2023). Symptoms of Myasthenia Gravis Obscured by Old Age and Unilateral Presentation. *Cureus*, *15*(9). https://doi.org/https://doi.org/10.7759/cureus.44737

Gupta, M., Gupta, O., & Vohra, V. (2014). Bilateral familial vertical Duane Syndrome with synergistic convergence, aberrant trigeminal innervation, and facial hypoplasia. *Oman Journal of Ophthalmology*, *7*(3), 135–137. https://doi.org/https://doi.org/10.4103/0974-620X.142596

Haehner, A., Hummel, T., Hummel, C., Sommer, U., Junghanns, S., & Reichmann, H. (2007). Olfactory loss may be a first sign of idiopathic Parkinson’s disease. *Movement Disorders*, *22*(6), 839–842.

Hale, E. D., Treharne, G. J., Norton, Y., Lyons, A. C., Douglas, K. M. J., Erb, N., & Kitas, G. D. (2006). “Concealing the evidence”: the importance of appearance concerns for patients with systemic lupus erythematosus. *Lupus*, *15*(8), 532–540. https://doi.org/https://doi.org/10.1191/0961203306lu2310xx

Hamaguchi, Y., Fujimoto, M., Matsushita, T., Kaji, K., Komura, K., Hasegawa, M., Kodera, M., Muroi, E., Fujikawa, K., & Seishima, M. (2013). Common and distinct clinical features in adult patients with anti-aminoacyl-tRNA synthetase antibodies: heterogeneity within the syndrome. *PloS One*, *8*(4), e60442.

Hanane, I., Assia, E. L. H., Alaoui, H., Daghouj, G., El Maaloum, L., Allali, B., & El Kettani, A. (2024). Syndromic congenital glaucoma clinical features and therapeutic results. *Acta Ophthalmologica*, *102*. https://doi.org/https://doi.org/10.1111/aos.16237

He, X., Wang, B., Jia, X., Li, Y., Yan, H., Mu, Q., & Chen, S. (2022). Persistent Facial Flushing in a Patient with Telangiectasia Macularis Eruptiva Perstans: An Unusual but Should Emphasized Clinical Finding. *Clinical, Cosmetic and Investigational Dermatology*, *15*, 1309–1312. https://doi.org/https://doi.org/10.2147/CCID.S371921

Hertz, R., Espinosa, J., Lucerna, A., & Stranges, D. (2017). Multiple Sclerosis Presenting with Facial Twitching (Myokymia and Hemifacial Spasms). *Case Reports in Neurological Medicine*, *2017*, 3. https://doi.org/https://doi.org/10.1155/2017/7180560

Hodgson, T. A. (2001). Diagnosing common tongue lesions. *Practitioner*, 340. https://www.proquest.com/trade-journals/diagnosing-common-tongue-lesions/docview/232977365/se-2?accountid=8113

Hu, J., Rui-qing, X. U., Xiang-long, L. Ã., Yun-qin, W., & Gao, Y. (2019). Clinical analysis of Wallenberg’s syndrome. *Chinese Journal of Contemporary Neurology & Neurosurgery*, *19*(1), 41–46. https://doi.org/https://doi.org/10.3969/j.issn.1672-6731.2019.01.010

Hu, Y., Zhong, M., & Hu, M. (2024). Varicellaâ€‘zoster virusâ€‘associated meningitis followed peripheral facial palsy: A case report. *Experimental and Therapeutic Medicine*, *28*(4). https://doi.org/https://doi.org/10.3892/etm.2024.12669

Hupert, C. (1984). Contralateral Facial Blush and Ipsilateral Facial Pallor Following Interscalene Brachial Block. *Regional Anesthesia*, *9*(4), 203–206. https://doi.org/https://doi.org/10.1136/rapm-00115550-198409040-00010

Hyams, J. S., Ferry, G. D., Mandel, F. S., Gryboski, J. D., Kibort, P. M., Kirschner, B. S., Griffiths, A. M., Katz, A. J., Grand, R. J., & Boyle, J. T. (1991). Development and validation of a pediatric Crohn’s disease activity index. *Journal of Pediatric Gastroenterology and Nutrition*, *12*(4), 439–439.

Inazawa-Terada, M., Namiki, T., Omigawa, C., Fujimoto, T., Munetsugu, T., Ugajin, T., Shimomura, Y., Ohshima, Y., Yoshida, K., Niizeki, H., Hayashi, R., Nakano, H., & Yokozeki, H. (2022). An epidemiological survey of anhidrotic/hypohidrotic ectodermal dysplasia in Japan: High prevalence of allergic diseases. *Journal of Dermatology*, *49*(4), 422–431. https://doi.org/10.1111/1346-8138.16278

Iscan, A., Kurul, S., & Dirik, E. (2005). Clinical findings of tuberous sclerosis in Turkish children1: JPN. *Journal of Pediatric Neurology*, *3*(2), 95–102. https://www.proquest.com/scholarly-journals/clinical-findings-tuberous-sclerosis-turkish/docview/217695282/se-2?accountid=8113

Ito, K., Imafuku, S., Hamaguchi, Y., Fujimoto, M., & Nakayama, J. (2013). Case report of anti-transcription intermediary factor-1-[gamma]/[alpha] antibody-positive dermatomyositis associated with gastric cancer and immunoglobulin G4-positive pulmonary inflammatory pseudotumor. *The Journal of Dermatology*, *40*(7), 567–569. https://www.proquest.com/scholarly-journals/case-report-anti-transcription-intermediary/docview/1372849528/se-2?accountid=8113

Jacome, D. E. (1991). Language-Apparent Reflex Epilepsy. *Clinical Eletroencephalography*, *22*(3).

Jacome, D. E. (2001). Dracula’s Teeth Syndrome. *Headache*, *41*(9), 892–894. https://www.proquest.com/scholarly-journals/draculas-teeth-syndrome/docview/218647549/se-2?accountid=8113

Jang, J. H., Hae Chang, J., Kim, K. Y., Jue, M. S., Kim, J. E., & Ko, J. Y. (2023). LP-058â€ The involvement of scalp and nails in cutaneous lupus erythematosus patients: a retrospective study. *Lupus Science & Medicine*, *10*, A109–A110. https://doi.org/https://doi.org/10.1136/lupus-2023-KCR.167

Jatania, H., & K, G. (2012). *Spectrum of Ocular Changes After Zygomatico-Maxillary Complex and Orbital Fractures* (Issue 30563978). https://www.proquest.com/dissertations-theses/spectrum-ocular-changes-after-zygomatico/docview/2848394315/se-2?accountid=8113

Jesuthasan, J., Srivickneswaran, G., & Selladurai, P. (2022). An unusual presentation of hemiparesis with complex ophthalmoplegia: A â€˜nineâ€™ syndrome. *SAGE Open Medical Case Reports*, *10*. https://doi.org/https://doi.org/10.1177/2050313X221135599

Joshi, A., Ghosh, S., Gunnery, S., Tickle-Degnen, L., Sclaroff, S., & Betke, M. (2018). *Context-Sensitive Prediction of Facial Expressivity Using Multimodal Hierarchical Bayesian Neural Networks* (pp. 278–285). The Institute of Electrical and Electronics Engineers, Inc. (IEEE). https://www.proquest.com/conference-papers-proceedings/context-sensitive-prediction-facial-expressivity/docview/2051137143/se-2?accountid=8113

Jung-Rern, J., Hao-Chien, W., Chang, Y.-C., & Yang, P.-C. (2003). Postural stridor. *The Lancet*, *362*(9385), 704. https://doi.org/https://doi.org/10.1016/S0140-6736(03)14233-X

Kajal, B., Harvey, J., & Alowami, S. (2013). Melkerrson-Rosenthal Syndrome, a rare case report of chronic eyelid swelling. *Diagnostic Pathology*, *8*, 188. https://doi.org/https://doi.org/10.1186/1746-1596-8-188

Kamoun, F., Hsairi, M., Sfaihi, L., Ameur, S. Ben, Hmida, N., Rgaieg, R., Chabchoub, I., Kamoun, T. H., Gargouri, A., & Hachicha, M. (2014). PO-0542â€ Congenital Rubella Still Exists In Tunisia. *Archives of Disease in Childhood*, *99*. https://doi.org/https://doi.org/10.1136/archdischild-2014-307384.1185

Karadan, U., Manappallil, R. G., Janardhanan, A., & Supreeth, R. N. (2018). Trigeminal trophic syndrome following anterior inferior cerebellar artery infarction. *BMJ Case Reports*, *2018*. https://doi.org/https://doi.org/10.1136/bcr-2018-225278

Kasravi, N. M. D., Leung, A. M. D., Silver, I. M. D. Ms., & Burneo, J. G. M. D. M. (2010). Dissection of the internal carotid artery causing Horner syndrome and palsy of cranial nerve XII: CMAJ. *Canadian Medical Association. Journal*, *182*(9), E373-7. https://www.proquest.com/scholarly-journals/dissection-internal-carotid-artery-causing-horner/docview/504023333/se-2?accountid=8113

Kim, J. K., Kim, B.-J., Shin, H. Y., Shin, K. J., Nam, T.-S., Seok, J. I., Suh, B. C., Oh, J., Kim, Y. J., & Bae, J. S. (2015). Does delayed facial involvement implicate a pattern of “descending reversible paralysis” in Fisher syndrome? *Clinical Neurology and Neurosurgery, Suppl. C*, *135*, 1–5. https://doi.org/https://doi.org/10.1016/j.clineuro.2015.04.025

Kiranmai, I. (2005). *Study of Lupus Nephritis: Clinical Course as Related to Morphological Forms and Outcome to Therapy* (Issue 27556505). https://www.proquest.com/dissertations-theses/study-lupus-nephritis-clinical-course-as-related/docview/2322393319/se-2?accountid=8113

Kishi, T., Tani, Y., Okiyama, N., Mizuochi, K., Ichimura, Y., Harigai, M., Nagata, S., & Miyamae, T. (2020). *Anti-SAE Autoantibody-Positive Japanese Patient with Juvenile Dermatomyositis complicated with Interstitial Lung Disease - A Case Report*. https://doi.org/https://doi.org/10.21203/rs.3.rs-83639/v1

Kister, I., Caminero, A. B., Monteith, T. S., Soliman, A., Bacon, T. E., Bacon, J. H., Kalina, J. T., Inglese, M., Herbert, J., & Lipton, R. B. (2010). Migraine is comorbid with multiple sclerosis and associated with a more symptomatic MS course: Official Journal of the Italian Society for the Study of Headaches. *The Journal of Headache and Pain*, *11*(5), 417–425. https://doi.org/https://doi.org/10.1007/s10194-010-0237-9

Knibbs, J. (2021). Parkinsonâ€™s disease: Facial masking an early warning sign - four body parts affected. *Express (Online)*. https://www.proquest.com/newspapers/parkinson-s-disease-facial-masking-early-warning/docview/2544328970/se-2?accountid=8113

Kravos, A., & Boltezar, I. H. (2010). Reinke’s edema of the vocal cords. *Zdravniski Vestnik*, *79*(12). https://www.proquest.com/scholarly-journals/reinkes-edema-vocal-cords/docview/1312446788/se-2?accountid=8113

Krishnan, K. G., Schackert, G., & Seifert, V. (2010). Outcomes of microneurovascular facial reanimation using masseteric innervation in patients with long-standing facial palsy resulting from cured brainstem lesions. *Neurosurgery*, *67*(3), 663–674. https://doi.org/10.1227/01.NEU.0000375531.77489.79

Kshirsagar, D. P., & Ingale, A. M. (2014). Lyme disease: Emerging and Re-emerging Metazoonoses of Gglobal Importance. *Journal of Animal Research*, *4*(1), 39–51. https://www.proquest.com/scholarly-journals/lyme-disease-emerging-re-metazoonoses-gglobal/docview/1550519354/se-2?accountid=8113

Kubik, M., Robles, L., & Kung, D. (2012). Familial Bell’s Palsy: A Case Report and Literature Review. *Case Reports in Neurological Medicine*, *2012*. https://doi.org/https://doi.org/10.1155/2012/674981

Kutbay, N. Ã., Erdemir, Z., YÃ¼rekli, B. S., Karaca, E., Erdogan, M., Ã‡etinkalp, S., Kandiloglu, G., Ã–zgen, A. G., Ã–zkinay, F., & Saygili, L. F. (2015). A Case of Dyskeratosis Congenita Associated with Hypothyroidism and Hypogonadism. *Journal of Clinical Research in Pediatric Endocrinology*, *7*(2). https://www.proquest.com/scholarly-journals/case-dyskeratosis-congenita-associated-with/docview/1789512799/se-2?accountid=8113

Lawley, T. J. (2008). *Atlas of skin Manifestations of internal disease*. The McGraw-Hill Companies.

Lei, C., Qu, M., Sun, H., Huang, J., Huang, J., Song, X., Zhai, G., & Zhou, H. (2023). Facial expression of patients with Graves’ orbitopathy. *Journal of Endocrinological Investigation*, *46*(10), 2055–2066. https://doi.org/10.1007/s40618-023-02054-y

Leon, E., Diaz, J., Castilla-Vallmanya, L., Grinberg, D., Balcells, S., & Urreizti, R. (2020). Extending the phenotypic spectrum of Bohring-Opitz syndrome: Mild case confirmed by functional studies. *American Journal of Medical Genetics, Part A*, *182*(1), 201–204. https://doi.org/10.1002/ajmg.a.61397

LeWit, P. (2017). Portrayal of progressive supranuclear palsy in the 16th century. *The Lancet - Neurology*, *16*, 956–957. https://doi.org/10.1148/radiol.2017161595

Lin, Q., Zhang, B., Zheng, W., Li, M., Zhao, Y., Zeng, X., Zhang, F., Wang, L., & Li, L. (2022). Clusters of clinical and immunologic features in patients with bullous systemic lupus erythematosus: experience from a single-center cohort study in China. *Orphanet Journal of Rare Diseases*, *17*, 1–10. https://doi.org/https://doi.org/10.1186/s13023-022-02445-z

Lis-Swiety, A., & Brzezinska, L. (2020). Frontal fibrosing alopecia: a disease that remains enigmatic. *Postepy Dermatologii i Alergologii*, *37*(4), 482–489. https://doi.org/https://doi.org/10.5114/ada.2020.98241

Mansouri, P., Masoumeh Rohani, N., Zahra Safaei, N., Shakoei, S., Chalangari, R., Katalin Martits, â€“Chalangari, & Rahbar, M. R. (2020). Facial Papules Are Early Sign of Frontal Fibrosing Alopecia: A Cross-Sectional Study. *Acta Medica Iranica*, *58*(6), 285–290. https://doi.org/https://doi.org/10.18502/acta.v58i6.4056

Marqusee, E., Benson, C. B., Frates, M. C., Doubilet, P. M., Larsen, P. R., Cibas, E. S., & Mandel, S. J. (2000). Usefulness of ultrasonography in the management of nodular thyroid disease. *Annals of Internal Medicine*, *133*(9), 696–700.

Marshall, S. E. (2004). Behçet’s disease. *Best Practice & Research Clinical Rheumatology*, *18*(3), 291–311.

Martinez-Molina, M., Carmona-Rocha, E., Gil-Lianes, J., Yubero, D., Casas-Alba, D., Baselga, E., & Ivars, M. (2024). Trichoscopic findings in neonatal alopecia in oro-facial-digital syndrome type 1. *Pediatric Dermatology*, *41*(6), 1199–1202. https://doi.org/10.1111/pde.15686

Mass, E. (2012). A review of the oro-dento-facial characteristics of hereditary sensory and autonomic neuropathy type III (familial dysautonomia). *Special Care in Dentistry*, *32*(1), 15–20. https://doi.org/https://doi.org/10.1111/j.1754-4505.2011.00225.x

Mavrikakis, I. (2011). History of Disease, Facial Nerve Grading Systems & Clinical Evaluation. *Acta Ophthalmologica*, *89*. https://doi.org/https://doi.org/10.1111/j.1755-3768.2011.1312.x

Melish, M. E., & Glasgow, L. A. (1970). The staphylococcal scalded-skin syndrome: development of an experimental model. *New England Journal of Medicine*, *282*(20), 1114–1119.

Melmon, K. L., & Rosen, S. W. (1964). Lindau’s disease: review of the literature and study of a large kindred. *The American Journal of Medicine*, *36*(4), 595–617.

Mercier, T., Deslypere, G., & Nackaerts, K. (2019). Ramsay Hunt syndrome: a rare complication of herpes zoster infection in a lung cancer patient. *Acta Clinica Belgica: International Journal of Clinical and Laboratory Medicine*, *74*(5), 355–358. https://doi.org/10.1080/17843286.2018.1517076

Mermerkaya, M., SÃ¼er, E., Ã–ztÃ¼rk, E., GÃ¼lpinar, Ã., GÃ¶kÃ§e, M. I., YalÃ§inda, F. N., SoygÃ¼r, T., & Burgu, B. (2014). Nocturnal lagophthalmos in children with urofacial syndrome (Ochoa): A novel sign. *European Journal of Pediatrics*, *173*(5), 661–665. https://doi.org/https://doi.org/10.1007/s00431-013-2172-7

Miest, R. Y. M. D., Bruce, A. J. Mbc., Comfere, N. I. M. D., Hadjicharalambous, E. M. D., Endly, D. D. O., Lohse, C. M. M. S., & Rogers, R. S. I. I. I. M. D. (2017). A Diagnostic Approach to Recurrent Orofacial Swelling: A Retrospective Study of 104 Patients. *Mayo Clinic Proceedings*, *92*(7), 1053–1060. https://doi.org/https://doi.org/10.1016/j.mayocp.2017.03.015

Migliaccio, A. A., Halmagyi, G. M., McGarvie, L. A., & Cremer, P. D. (2004). Cerebellar ataxia with bilateral vestibulopathy: description of a syndrome and its characteristic clinical sign. *Brain*, *127*(2), 280–293.

Moon, S., Israeli, A., & Daze, R. (2022). Rapidly Progressive Erythroderma. *American Family Physician*, *105*(1), 75–76. https://www.proquest.com/scholarly-journals/rapidly-progressive-erythroderma/docview/2631931443/se-2?accountid=8113

Morrison, D. A., Bibby, K., & Woodruff, G. (1997). The “harlequin” sign and congenital Horner’s syndrome. In *Neurosurgery, and Psychiatry* (Vol. 62).

Mourits, M. P. H., Koornneef, L., Wiersinga, W. M., Prummel, M. F., Berghout, A., & Van Der Gaag, R. (1989). Clinical criteria for the assessment of disease activity in Graves’ ophthalmopathy: a novel approach. *British Journal of Ophthalmology*, *73*(8), 639–644.

Mulazimoglu, S., Flury, R., Kapila, S., & Linder, T. (2017). Effects of a sensory branch to the posterior external ear canal: coughing, pain, Ramsay Hunt’s syndrome and Hitselberger’s sign. *The Journal of Laryngology and Otology*, *131*(4), 329–333. https://doi.org/https://doi.org/10.1017/S0022215117000160

Mulla, O., Prowse, S., Sanders, T., & Nix, P. (2012). Epistaxis. *BMJ : British Medical Journal (Online)*, *344*. https://doi.org/https://doi.org/10.1136/bmj.e1097

Nance, M. A., & Berry, S. A. (1992). Cockayne syndrome: review of 140 cases. *American Journal of Medical Genetics*, *42*(1), 68–84.

Nerurkar, A. C., & Sheth, M. (2017). Facial, Oromotor and Speech disturbances in individuals with Parkinsonâ€™s disease. *International Journal of Therapies and Rehabilitation Research*, *6*(2), 217–222. https://doi.org/https://doi.org/10.5455/ijtrr.000000268

Nishizawa, T., Ishikawa, K., Matsuo, T., Higuchi, N., Ishiguro, K., & Mori, N. (2021). Atypical Ramsay Hunt syndrome (zoster sine herpete) with otitis media. *Journal of General and Family Medicine*, *22*(6), 344–346. https://doi.org/10.1002/jgf2.433

Nystrom, A. M., Ekvall, S., Allanson, J., Edeby, C., Elinder, M., Holmstrom, G., Bondeson, M. L., & Annerén, G. (2009). Noonan syndrome and neurofibromatosis type I in a family with a novel mutation in NF1. *Clinical Genetics*, *76*(6), 524–534. https://doi.org/https://doi.org/10.1111/j.1399-0004.2009.01233.x

Odaka, M., Yuki, N., Yamada, M., Koga, M., Takemi, T., Hirata, K., & Kuwabara, S. (2003). Bickerstaff’s brainstem encephalitis: clinical features of 62 cases and a subgroup associated with Guillain-Barre syndrome. *Brain*, *126*(10), 2279–2290. https://www.proquest.com/scholarly-journals/bickerstaffs-brainstem-encephalitis-clinical/docview/195430902/se-2?accountid=8113

Ohashi, N., Nonami, J., Kodaira, M., Yoshida, K., & Sekijima, Y. (2020). Taste disorder in facial onset sensory and motor neuronopathy: a case report. *BMC Neurology*, *20*, 1–5. https://doi.org/https://doi.org/10.1186/s12883-020-01639-x

Ohko, K., Nakajima, K., Nakajima, H., Hiraki, Y., Kubota, K., Fukao, T., Miyatake, S., Matsumoto, N., & Sano, S. (2020). Skin and hair abnormalities of Cantu syndrome: A congenital hypertrichosis due to a genetic alteration mimicking the pharmacological effect of minoxidil. *Journal of Dermatology*, *47*(3), 306–310. https://doi.org/10.1111/1346-8138.15216

Ohlson, M. W. O. D. F. (2018). ODs must examine more than just eyes. *Optometry Times*, *10*(12), 10–15. https://www.proquest.com/scholarly-journals/ods-must-examine-more-than-just-eyes/docview/2168093078/se-2?accountid=8113

Ouattara, B., Kouakou, K. R., Koffi, K. M., Harding-Kaba, M. B., Boka, K. L., Daweni, D., & Moudalbaye, N. S. (2018). Rose Cephalic Tetanus About 2 Cases in the Stomatological and Surgical Maxillo-Facial of The Teaching Hospital of Cocody (RCI). *Indian Journal of Stomatology*, *9*(1), 19–21. https://www.proquest.com/scholarly-journals/rose-cephalic-tetanus-about-2-cases/docview/2188845279/se-2?accountid=8113

Ozekmekçi, S., Benbir, G., Ozdogan, F. Y., Ertan, S., & Kiziltan, M. E. (2007). Hemihypomimia, a rare persistent sign in Parkinson’s disease: Follow up of 11 patients. *Journal of Neurology*, *254*(3), 347–350. https://doi.org/https://doi.org/10.1007/s00415-006-0372-z

Pagella, P., JimÃ©nez-rojo, L., & Mitsiadis, T. A. (2014). Roles of innervation in developing and regenerating orofacial tissues. *Cellular and Molecular Life Sciences*, *71*(12), 2241–2251. https://doi.org/https://doi.org/10.1007/s00018-013-1549-0

Pamnani, S., Bakshi, S. S., & Acharya, S. (2022). Toxic Epidermal Necrolysis: A Case Report on a Drug-Induced Phenomenon. *Cureus*, *14*(10). https://doi.org/https://doi.org/10.7759/cureus.30407

Panchangam, R. B., Kota, S. K., & Mayilvaganan, S. (2021). Selfie-driven thyroid disease leads: A study on a unique sign and its utility in clinical practice. *Annals of African Medicine*, *20*(4), 293–296. https://doi.org/10.4103/aam.aam_64_20

Panchaprateep, R., Ruxrungtham, P., Chancheewa, B., & Asawanonda, P. (2020). Clinical characteristics, trichoscopy, histopathology and treatment outcomes of frontal fibrosing alopecia in an Asian population: A retroâ€prospective cohort study. *The Journal of Dermatology*, *47*(11), 1301–1311. https://doi.org/https://doi.org/10.1111/1346-8138.15517

Parajuli, N., & Shrestha, S. (2022). Facial Tuberculoid Leprosy. In *American Journal of Tropical Medicine and Hygiene* (Vol. 106, Issue 5, pp. 1296–1297). American Society of Tropical Medicine and Hygiene. https://doi.org/10.4269/ajtmh.21-0696

Pasquini, L., Tortora, D., Manunza, F., Rossi Espagnet, M. C., Figà-Talamanca, L., Morana, G., Occella, C., Rossi, A., & Severino, M. (2019). Asymmetric cavernous sinus enlargement: a novel finding in Sturge–Weber syndrome. *Neuroradiology*, *61*(5), 595–602. https://doi.org/10.1007/s00234-019-02182-4

Patra, S., Purkait, R., Samanta, T., & Bhadra, R. (2013). Varadi Papp syndrome, an unusual variant of oral-facial-digital syndrome: Report of a rare case. *Annals of Indian Academy of Neurology*, *16*(2), 289–291. https://doi.org/https://doi.org/10.4103/0972-2327.112502

Patterson, M. C., Hendriksz, C. J., Walterfang, M., Sedel, F., Vanier, M. T., Wijburg, F., & Group, N.-C. G. W. (2012). Recommendations for the diagnosis and management of Niemann–Pick disease type C: an update. *Molecular Genetics and Metabolism*, *106*(3), 330–344.

Pavone, P., Garozzo, R., Trifeletti, R. R., & Parano, E. (1999). Marin-Amat syndrome: Case report and review of the literature. *Journal of Child Neurology*, *14*(4), 266–268. https://www.proquest.com/scholarly-journals/marin-amat-syndrome-case-report-review-literature/docview/220416098/se-2?accountid=8113

Pearce, J. M. S. (2005). Romberg and His Sign. *European Neurology*, *53*(4), 210–213. https://www.proquest.com/scholarly-journals/romberg-his-sign/docview/194942503/se-2?accountid=8113

Pedraza, M. I., Barbado, J., Ruiz, M., & Guerrero, Ã. L. (2015). Posterior Reversible Encephalopathy Syndrome as Presenting Form of Very Early Systemic Sclerosis. *Case Reports in Neurological Medicine*, *2015*. https://doi.org/https://doi.org/10.1155/2015/290378

Persily, T., & Collins, P. (2019). *Photo Quiz Papular Lesions on Bilateral Elbows and Knees* (Vol. 99, Issue 1). www.aafp.org/afpAmericanFamilyPhysician47

Pinto-Almeida, T., Caetano, M., Sanches, M., & Selores, M. (2013). Cutaneous manifestations of antiphospholipid syndrome: a review of the clinical features, diagnosis and management. *Acta Reumatológica Portuguesa*, *38*, 10–18.

Pirola, F. J. C., Santos, B. A. M., Sapienza, G. F., Cetrangolo, L. Y., Geranutti, C. H. W. G., & Aguiar, P. H. P. de. (2022). Miller-Fisher syndrome after first dose of Oxford/AstraZeneca coronavirus disease 2019 vaccine: a case report. *Journal of Medical Case Reports*, *16*, 1–4. https://doi.org/https://doi.org/10.1186/s13256-022-03592-4

Polat, A., Dinulescu, M., Fraitag, S., Nimubona, S., Toutain, F., Jouneau, S., Poullot, E., Droitcourt, C., & Dupuy, A. (2018). Skin manifestations among GATA2-deficient patients. *British Journal of Dermatology*, *178*(3), 781–785. https://doi.org/10.1111/bjd.15548

Polat, M., Tug, E., Atasoy, H. I., & Parlak, A. H. (2012). Hemifasiyal Atrofinin Eslik Ettigi Harlequin Sendromu/Harlequin Syndrome With Hemifacial Atrophy: A Case Study. *Turkderm*, *46*(1), 50–52. https://www.proquest.com/scholarly-journals/hemifasiyal-atrofinin-eslik-ettigi-harlequin/docview/1009889797/se-2?accountid=8113

Ponsen, M. M., Stoffers, D., Booij, J., van Eck‐Smit, B. L. F., Wolters, E. C., & Berendse, H. W. (2004). Idiopathic hyposmia as a preclinical sign of Parkinson’s disease. *Annals of Neurology: Official Journal of the American Neurological Association and the Child Neurology Society*, *56*(2), 173–181.

Pratiksha Shankarlal, N. (2022). IDDF2022-ABS-0188â€ Wilsonâ€™s disease in patients with IGA nephropathy: a systematic review of published cases. *Gut*, *71*, A92–A93. https://doi.org/https://doi.org/10.1136/gutjnl-2022-IDDF.117

Prieur, A.-M., Griscelli, C., Lampert, F., Truckenbrodt, H., Guggenheim, M. A., Lovell, D. J., Pelkonnen, P., Chevrant-Breton, J., & Ansell, B. M. (1987). A chronic, infantile, neurological, cutaneous and articular (CINCA) syndrome. A specific entity analysed in 30 patients. *Scandinavian Journal of Rheumatology*, *16*, 57–68.

Putoux, A., Nampoothiri, S., Laurent, N., Cormier-Daire, V., Beales, P. L., Schinzel, A., Bartholdi, D., Alby, C., Thomas, S., Elkhartoufi, N., Ichkou, A., Litzler, J., Munnich, A., Encha-Razavi, F., Kannan, R., Faivre, L., Boddaert, N., Rauch, A., Vekemans, M., & AttiÃ©-Bitach, T. (2012). Novel KIF7 mutations extend the phenotypic spectrum of acrocallosal syndrome. *Journal of Medical Genetics*, *49*(11), 713. https://doi.org/https://doi.org/10.1136/jmedgenet-2012-101016

Pyykko, I., Ziane, S., & Zou, J. (2012). Low-Frequency Sound Pressure and Transtympanic Endoscopy of the Middle Ear in Assessment of “Spontaneous” Perilymphatic Fistula. *ISRN Otolaryngology*. https://www.proquest.com/scholarly-journals/low-frequency-sound-pressure-transtympanic/docview/1038346332/se-2?accountid=8113

Rafailidis, P. I., Kapaskelis, A., & Falagas, M. E. (2007). Periorbital and facial swelling due to dermatomyositis: CMAJ. *Canadian Medical Association. Journal*, *176*(11), 1580–1581. https://www.proquest.com/scholarly-journals/periorbital-facial-swelling-due-dermatomyositis/docview/204850525/se-2?accountid=8113

Rana, P. V, & Wadia, R. S. (1985). The Marin-Amat syndrome: an unusual facial synkinesia. *Journal of Neurology, Neurosurgery and Psychiatry*, *48*(9), 939. https://doi.org/https://doi.org/10.1136/jnnp.48.9.939

Renee H. Grau, M. D. and R. D. S. M. D. (2007). Determining how skin changes relate to systemic manifestations -- Cutaneous cues to diagnosis of lupus. *The Journal of Musculoskeletal Medicine*, *24*(6), 247. https://www.proquest.com/scholarly-journals/determining-how-skin-changes-relate-systemic/docview/195143859/se-2?accountid=8113

Rijnenberg, D., Van der Heijden, E., Leavis, H., & Roon, J. Van. (2024). POS1253â€ LIDOCAINE-INDUCED TEAR PRODUCTION IN SJOGRENâ€™S DISEASE: BROADENING THE HORIZON FOR TREATMENT OF OCULAR DRYNESS? *Annals of the Rheumatic Diseases*, *83*, 869. https://doi.org/https://doi.org/10.1136/annrheumdis-2024-eular.1905

Rossi, M., Wilken, M., Morisset, P., FariÃ±a, S., Cerquetti, D., & Merello, M. (2016). Facial tremors in patients with and without parkinsonism. *Neurological Sciences*, *37*(12), 1999–2002. https://doi.org/https://doi.org/10.1007/s10072-016-2683-x

Saito, S., Endo, Y., Nishio, M., Uchiyama, A., Uehara, A., Toki, S., Yasuda, M., Ishikawa, O., Muro, Y., & Motegi, S. ichiro. (2022). Anti-polymyositis/Scl antibody-positive overlap syndrome of diffuse cutaneous systemic sclerosis, dermatomyositis, systemic lupus erythematosus, and antiphospholipid syndrome. *Journal of Dermatology*, *49*(2), 294–298. https://doi.org/10.1111/1346-8138.16219

Saniasiaya, J. (2022). Mediastinal lymphoma presenting with asymmetrical chest wall. *BMJ Case Reports*, *15*(1). https://doi.org/https://doi.org/10.1136/bcr-2021-246953

Schott, J. M., & Rossor, M. N. (2016). The palmomental reflex: stop scratching around! *Practical Neurology*, *16*(6), 500. https://doi.org/https://doi.org/10.1136/practneurol-2016-001509

Schweiger, B. M., Esakhan, C. L., Frishberg, D., Grand, K., Garg, R., & Sanchez-Lara, P. A. (2021). Pediatric Cushing syndrome: An early sign of an underling cancer predisposition syndrome. *American Journal of Medical Genetics, Part A*, *185*(9), 2824–2828. https://doi.org/10.1002/ajmg.a.62255

Sen, S., Sinhamahapatra, P., Choudhury, S., Gangopadhyay, A., Bala, S., Sircar, G., Chatterjee, G., & Ghosh, A. (2014). Cutaneous manifestations of mixed connective tissue disease: Study from a tertiary care hospital in Eastern India. *Indian Journal of Dermatology*, *59*(1), 35–40. https://doi.org/https://doi.org/10.4103/0019-5154.123491

Sévin, M., Lesca, G., Baumann, N., Millat, G., Lyon-Caen, O., Vanier, M. T., & Sedel, F. (2007). The adult form of Niemann–Pick disease type C. *Brain*, *130*(1), 120–133.

Sidell, D., Tanna, N., & Preciado, D. (2006). Tonsillar inclusion cysts in Gorlin’s syndrome. *Ear, Nose & Throat Journal*, *85*(12), 818. https://www.proquest.com/scholarly-journals/tonsillar-inclusion-cysts-gorlins-syndrome/docview/209404574/se-2?accountid=8113

Silva Riveiro, A., Nieto GonzÃ¡lez, J. C., MartÃnez Barrio, J., LÃ^3^pez-CerÃ^3^n CofiÃ±o, A., Torrens Cid, L. A., Soleto Kharkovskaya, C., Serrano Benavente, B., GonzÃ¡lez BenÃtez, R. D., GonzÃ¡lez FernÃ¡ndez, C. M., LÃ^3^pez-Longo, F. J., & Monteagudo SÃ¡ez, I. (2018). AB0721 Transfer of systemic sclerosis after allogeneic bone marrow transplantation. *Annals of the Rheumatic Diseases, Suppl. Supplement 2*, *77*, 1499. https://doi.org/https://doi.org/10.1136/annrheumdis-2018-eular.7042

Sivesind, T. E., Szeto, M. D., Shahzeb, H., Tugwell, P., & Dellavalle, R. P. (2022). Mortality Outcomes in Dermatology: An Exploration of Core Outcome Sets and Cochrane Skin Systematic Reviews. *JMIR Dermatology*, *5*(1). https://doi.org/https://doi.org/10.2196/34140

Smith, R. P., Schoen, R. T., Rahn, D. W., Sikand, V. K., Nowakowski, J., Parenti, D. L., Holman, M. S., Persing, D. H., & Steere, A. C. (2002). Clinical characteristics and treatment outcome of early Lyme disease in patients with microbiologically confirmed erythema migrans. *Annals of Internal Medicine*, *136*(6), 421–428.

Solomon, B. D., & Muenke, M. (2012). *When to Suspect a Genetic Syndrome*.

Sone, I., Honda, T., Sakuraba, M., Satoh, K., Kuwajima, Y., Baba, S., & Wada, Y. (2024). Goltz Syndrome Combined with Triple X Syndrome, a Case Report. *Cleft Palate Craniofacial Journal*, *61*(3), 534–538. https://doi.org/10.1177/10556656221141236

Strong, A., Qu, H. Q., Cullina, S., McManus, M. L., Zackai, E. H., Glessner, J., Kenny, E. E., & Hakonarson, H. (2023). TOPORS as a novel causal gene for Joubert syndrome. *American Journal of Medical Genetics, Part A*, *191*(8), 2156–2163. https://doi.org/10.1002/ajmg.a.63303

Syrine, Z., Feki, A., Jemaa, S. Ben, Mabrouk, Y., Cyrine, A., Ezzeddine, M., Kallel, M. H., Fourati, H., Akrout, R., & Baklouti, S. (2023). AB1749-PAREâ€ IMPACT OF FACIAL SIGNS OF SYSTEMIC SCLEROSIS ON PATIENTSâ€™ SELF-ESTEEM. *Annals of the Rheumatic Diseases*, *82*, 2110. https://doi.org/https://doi.org/10.1136/annrheumdis-2023-eular.4702

Takayama, R., Ueno, T., & Saeki, H. (2017). Immunoglobulin G4-related disease and its skin manifestations. *The Journal of Dermatology*, *44*(3), 288–296. https://doi.org/https://doi.org/10.1111/1346-8138.13723

Tanimura, J., & Terakawa, I. (2023). Hoagland’s Sign as a Manifestation of Parvovirus B19 Infection. *Cureus*, *15*(8). https://doi.org/https://doi.org/10.7759/cureus.43925

Unknown. (1998). A guide to the management of chronic sinusitis. *Practitioner*, 712. https://www.proquest.com/trade-journals/guide-management-chronic-sinusitis/docview/232979972/se-2?accountid=8113

Unknown. (2001). BUSINESS LINE: India: Sceptical marketing. *Businessline*, 1. https://www.proquest.com/trade-journals/business-line-india-sceptical-marketing/docview/221691852/se-2?accountid=8113

Urushitani, M., Udaka, F., & Kameyama, M. (1995). Miller Fisher-Guillain-BarrÃ© overlap syndrome with enhancing lesions in the spinocerebellar tracts. *Journal of Neurology, Neurosurgery and Psychiatry*, *58*(2), 241. https://doi.org/https://doi.org/10.1136/jnnp.58.2.241

Valenzuela, B., Arevalo, J., Contreras, W., & Martínez, F. (2022). A Spatio-Temporal Hypomimic Deep Descriptor to Discriminate Parkinsonian Patients. *Proceedings of the Annual International Conference of the IEEE Engineering in Medicine and Biology Society, EMBS*, *2022-July*, 4192–4195. https://doi.org/10.1109/EMBC48229.2022.9871753

Viswanath, O., & Aner, M. (2018). Dramatic extremity temperature increase and Horner’s syndrome after stellate ganglion block. *Postgraduate Medical Journal*, *94*(1110), 237. https://doi.org/https://doi.org/10.1136/postgradmedj-2017-135292

Wai Foong, H., Kyndt, C., Faragher, M., & Day, B. (2018). 029â€ Multiple-acyl-coa dehydrogenase deficiency (MADD): a rare but treatable genetic metabolic myopathy. *Journal of Neurology, Neurosurgery and Psychiatry*, *89*(6). https://doi.org/https://doi.org/10.1136/jnnp-2018-ANZAN.28

Wang, Y., Guo, X., Liu, J., & Xing, B. (2023). Skin manifestations and submacroscopical features of acromegaly: A case-control study using dermoscopy and high-frequency ultrasound. *Skin Research and Technology*, *29*(4). https://doi.org/10.1111/srt.13319

Wheeler, T. T., Alberts, M. A. M., Dolan, T. A., & McGorray, S. P. (1995). Dental, visual, auditory and olfactory complications in Paget’s disease of bone. *Journal of the American Geriatrics Society*, *43*(12), 1384. https://www.proquest.com/scholarly-journals/dental-visual-auditory-olfactory-complications/docview/210371197/se-2?accountid=8113

Whybra, C., Kampmann, C. H. R., Willers, I., Davies, J., Winchester, B., Kriegsmann, J., Brühl, K., Gal, A., Bunge, S., & Beck, M. (2001). Anderson–Fabry disease: clinical manifestations of disease in female heterozygotes. *Journal of Inherited Metabolic Disease*, *24*, 715–724.

Xiang, J.-Y., Zhang, Y.-H., Tan, Z.-R., Huang, J., & Zhao, Y.-W. (2014). Guillain-BarrÃ© Syndrome Associated with Japanese Encephalitis Virus Infection in China. *Viral Immunology*, *27*(8), 418–420. https://doi.org/https://doi.org/10.1089/vim.2014.0049

Young Il, R. (2014). Overlapping Guillain-BarrÃ© syndrome and Bickerstaff’s brainstem encephalitis associated with Epstein Barr virus. *Korean Journal of Pediatrics*, *57*(10), 457–460. https://doi.org/https://doi.org/10.3345/kjp.2014.57.10.457

Yuki, N., Odaka, M., Koga, M., Takemi, T., Hirata, K., & Kuwabara, S. (2004). Bickerstaff’s brainstem encephalitis: clinical features of 62 cases and a subgroup associated with Guillain-Barre syndrome. *American Journal of Ophthalmology*, *137*(2), 391. https://doi.org/https://doi.org/10.1016/j.ajo.2003.11.043

Zadro, I., Barun, B., Habek, M., & Brinar, V. V. (2008). Isolated cranial nerve palsies in multiple sclerosis. *Clinical Neurology and Neurosurgery*, *110*(9), 886–888. https://doi.org/https://doi.org/10.1016/j.clineuro.2008.02.009

Zaidan, T. F., Gorial, F. I., & Mohammed Ali, N. S. (2013). AB0659 Oral manifestations, oral health status, and salivary changes in iraqi systemic lupus erythematosus. *Annals of the Rheumatic Diseases, Suppl. 3*, *71*, 676. https://doi.org/https://doi.org/10.1136/annrheumdis-2012-eular.659

Zielińska, M., & Zielińska, R. (2019). Peripheral facial nerve palsy as a manifestation of neuroborreliosis in children. *Paediatrics and Family Medicine*, *15*(2), 131-131â€“133. https://doi.org/https://doi.org/10.15557/PiMR.2019.0022

Zivkovic, V., Lekovic, A., & Nikolic, S. (2022). Hyposphagma, positional asphyxia, and acute intoxication with psychoactive substances. *Journal of Forensic Sciences*, *67*(6), 2492–2496. https://doi.org/https://doi.org/10.1111/1556-4029.15141

Zouboulis CC. Epidemiology of Adamantiades-Behçet's disease. Ann Med Interne (Paris). 1999 Oct;150(6):488-98. PMID: 10615535.
