# Supplementary material for: Head and Neck Clinical Signs Associated With Diseases: A Scoping Review
Source: Spec Care Dentist. 2026 May 14;46:e70185. doi: 10.1111/scd.70185 (PMC13176508; doi:10.1111/scd.70185)
Supplement: Supplementary file 5 — Supplementary Material 5: Distribution of Reported Clinical Signs According to the World Bank Country Income Classification. [file SCD-46-0-s005.docx]

**Supplementary Material 5 –** Distribution of reported clinical signs according to the World Bank country income classification

| **Clinical sign** | **High-income** | **Middle-income** | **Low-income** |
| --- | --- | --- | --- |
| Blue sclera | 2^31,33^ | 0 | 0 |
| Coloboma | 3^30,55,68^ | 0 | 0 |
| One-and-a-half syndrome | 0 | 1^44^ | 0 |
| Heliotropic erythema / sign | 2^36,64^ | 0 | 0 |
| Horner's syndrome | 5^28,50,51,56,59^ | 0 | 0 |
| Kayser-Fleischer ring | 1^23^ | 1^42^ | 0 |
| Megalocornea | 1^21^ | 0 | 0 |
| Sclerocornea | 1^61^ | 0 | 0 |
| Supranuclear gaze palsy | 1^60^ | 0 | 0 |
| Acantose nigricans | 1^25^ | 0 | 0 |
| Café au lait lesions / macules | 1^62^ | 1^45^ | 0 |
| Harlequin syndrome | 1^27^ | 0 | 0 |
| Lupus pernio | 0 | 1^67^ | 0 |
| Malar rash | 1^34^ | 0 | 0 |
| Multiple facial trichilemmomas | 2^15,26^ | 0 | 0 |
| Nasal tip necrosis | 1^24^ | 0 | 0 |
| Pemberton's sign | 2^18,65^ | 0 | 0 |
| Port wine sign | 1^29^ | 0 | 0 |
| Raspberry-like papilomas | 0 | 1^43^ | 0 |
| Capillary malformation of the lower lip | 1^48^ | 0 | 0 |
| Cocaine-induced  midline destructive lesions | 1^32^ | 0 | 0 |
| False cheilitis | 0 | 1^58^ | 0 |
| Multiple neurofibroma | 1^17^ | 1^45^ | 0 |
| Multiple osteomas | 0 | 1^45^ | 0 |
| Multiple papillomatous nodules / oral papillomas | 1^26^ | 1^45^ | 0 |
| Palatal defect | 1^19^ | 0 | 0 |
| Perioral frecklin | 0 | 1^51^ | 0 |
| Recurrent oral ulceration | 1^47^ | 0 | 0 |
| Strawberry tongue | 4^22,40l52,57^ | 0 | 0 |
| Tongue hamartoma, multiple frenula, cleft lip/palate, upper lip notch | 1^66^ | 0 | 0 |
| Unilateral cyanosis of the tongue | 0 | 1^63^ | 0 |
| Erythematous lesion of the retroauricular skin | 1^54^ | 0 | 0 |
| Frank sign | 3^20,38,44^ | 0 | 0 |
| Milian ear sign | 0 | 1^41^ | 0 |
| Erythematous lesion of the skin | 1^16^ | 0 | 0 |
| Congenital infiltrating lipomatosis of the face | 1^53^ | 0 | 0 |
| Moon face | 1^37^ | 0 | 0 |
| Septal perforation | 1^45^ | 0 | 0 |
| Tullio’s phenomenon | 1^35^ | 0 | 0 |
| Unilateral facial swelling and vesicles | 1^39^ | 0 | 0 |
